# CRISPRi-ART enables functional genomics of diverse bacteriophages using RNA-binding dCas13d

**DOI:** 10.1038/s41564-025-01935-7

**Published:** 2025-02-26

**Authors:** Benjamin A. Adler, Muntathar J. Al-Shimary, Jaymin R. Patel, Emily G. Armbruster, David Colognori, Emeric J. Charles, Kate V. Miller, Arushi Lahiri, Michael L. Cui, Agnès Oromí-Bosch, Angela Voelker, Marena Trinidad, Jina Lee, Sebastien Beurnier, Ron Boger, Jason Nomburg, Rodolphe Barrangou, Vivek K. Mutalik, Joseph S. Schoeniger, Joseph A. Pogliano, David F. Savage, Jennifer A. Doudna, Brady F. Cress

**Affiliations:** 1 California Institute for Quantitative Biosciences (QB3), University of California, Berkeley, CA USA; 2 Innovative Genomics Institute, University of California, Berkeley, CA USA; 3 Department of Molecular and Cell Biology, University of California, Berkeley, CA USA; 4 School of Biological Sciences, University of California, San Diego, La Jolla, CA USA; 5 Graduate Group in Biophysics, University of California, Berkeley, CA USA; 6 Gladstone Institute of Data Science and Biotechnology, San Francisco, CA USA; 7 Department of Food, Bioprocessing and Nutrition Sciences, North Carolina State University, Raleigh, NC USA; 8 Environmental Genomics and Systems Biology Division, Lawrence Berkeley National Laboratory, Berkeley, CA USA; 9 Systems Biology Department, Sandia National Laboratories, Livermore, CA USA; 10 Howard Hughes Medical Institute, University of California, Berkeley, CA USA; 11 Department of Chemistry, University of California, Berkeley, CA USA; 12 MBIB Division, Lawrence Berkeley National Laboratory, Berkeley, CA USA

**Keywords:** Phage biology, Genomics, High-throughput screening

## Abstract

Bacteriophages constitute one of the largest reservoirs of genes of unknown function in the biosphere. Even in well-characterized phages, the functions of most genes remain unknown. Experimental approaches to study phage gene fitness and function at genome scale are lacking, partly because phages subvert many modern functional genomics tools. Here we leverage RNA-targeting dCas13d to selectively interfere with protein translation and to measure phage gene fitness at a transcriptome-wide scale. We find CRISPR Interference through Antisense RNA-Targeting (CRISPRi-ART) to be effective across phage phylogeny, from model ssRNA, ssDNA and dsDNA phages to nucleus-forming jumbo phages. Using CRISPRi-ART, we determine a conserved role of diverse rII homologues in subverting phage Lambda RexAB-mediated immunity to superinfection and identify genes critical for phage fitness. CRISPRi-ART establishes a broad-spectrum phage functional genomics platform, revealing more than 90 previously unknown genes important for phage fitness.

## Abstract (web summary)

Leveraging RNA-targeting dCas13d enables selective interference with phage protein translation and facilitates measurement of phage gene fitness at a transcriptome-wide scale.

## Main

Bacteriophages (phages) are the most abundant and genetically diverse entities on Earth, driving a genetic arms race with their bacterial hosts that continually alters microbial life, shaping both human health and the biosphere^1,2^. This phage–host arms race continually generates new protein functions encoded by uncharacterized genes that constitute a large source of genes of unknown function in the biosphere^3,4^. To expedite characterization of the vastly underexplored genetic content of phage genomes, genome-wide experimental approaches are needed.

Genome-scale CRISPRi (clustered regularly interspaced short palindromic repeats interference) methods are a common starting point for probing gene functions in diverse organisms by programmably blocking transcription using a nuclease-deactivated Cas9 or Cas12 (dCas9/dCas12)^5–11^. Recently, a dCas12-based DNA-targeting CRISPRi method was used to laboriously map essential genes for two model temperate phages one gene at a time^12^, but the arrayed assay format is cumbersome to scale. Furthermore, studies on nuclease-active Cas9 and Cas12 systems suggest limitations with DNA-targeting CRISPR systems when extended to lytic phages with distinct lifestyles, genomic content and genome modifications^13–21^. However, phage transcripts appear generally targetable and vulnerable during infection^21^. We posited that the RNA-guided RNA-binding protein dRfxCas13d (HEPN-deactivated *Ruminococcus flavefaciens* Cas13d, dCas13d)^22^ could be applied as a universal tool for targeted inhibition of phage protein expression, including for RNA phages and nucleus-forming phages where DNA-binding tools are completely ineffective^23^.

Here we present CRISPR interference through antisense RNA targeting (CRISPRi-ART) as a robust method for suppressing protein expression. By targeting dCas13d to phage transcript-encoded ribosome-binding sites (RBS), we could achieve targeted gene expression knockdown in diverse phages. Through pooled CRISPRi-ART libraries, we implemented transcriptome-wide CRISPRi-ART screens against diverse coliphages at unprecedented scale. We identified many previously unknown phage genes critical for infection, establishing a platform for high-throughput discovery and prioritizing genes for future study.

## Results

### Targeting dCas13d to bacterial RBSs represses protein expression

**Fig. 1:**
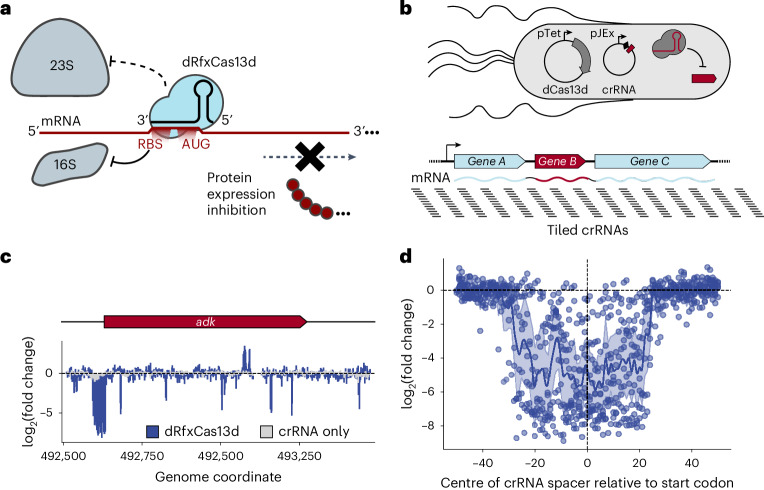
Design rules for dCas13d targeting by single-nucleotide-resolution profiling of *E. coli* essential genes. **a**, Overview of CRISPRi-ART. dRfxCas13d binding near the RBS reduces protein expression through inhibition of translation initiation by the 16S ribosomal subunit. **b**, A CRISPRi-ART (top) crRNA library tiled at single-nucleotide resolution against *E. coli* transcripts encoding essential genes (bottom) is used to identify regions susceptible to translational knockdown. Here, dCas13d expression is driven by the pTet promoter under aTc-inducible control, with its crRNA driven by the pJEx promoter under crystal violet-inducible control. *E. coli* growth is inhibited if dCas13d targets an essential gene’s RBS, while growth is unimpeded if the targeted protein is dispensable. **c**, The measured log_2_(fold-change) of guide abundances targeting a representative transcript encoding an essential gene is plotted. CRISPRi-ART-dependent fitness effects are presented in blue and crRNA-only controls in light grey (*n* = 3 independent replicates). **d**, The observed log_2_(fold-change) of crRNA abundances targeting the RBS region of 9 essential genes is compiled into a single plot, where points represent the centre of the crRNA spacer. Highlighted is the 100 bp surrounding the start codon (see Supplementary Fig. 3 showing a wider targeting range). Average of values at each nucleotide position are plotted across the region, along with a 95% confidence interval. Source data

To determine the principles governing translational repression by dCas13d binding to target messenger RNA (mRNA) sequences (Fig. 1a)^22^, we systematically identified the regions within mRNA transcripts that are most susceptible to dCas13d-mediated translational repression. The PAM-less nature of dCas13d^24^ enabled use of a pooled, single-nucleotide-resolution CRISPR RNA (crRNA) library (Supplementary Fig. 1) under the crystal violet (CV)-inducible pJEx promoter, tiled across 18 *E. coli* transcripts, most of which encode at least one essential gene (Fig. 1b). This 29,473-crRNA library was transformed into cells expressing dCas13d under the aTc-inducible pTet promoter, and 15 cell doublings after induction, samples were Illumina sequenced to quantify changes in crRNA abundance between the initial and final timepoints. This competitive growth assay revealed a major dCas13d-dependent fitness defect for crRNAs binding near (within ~70 nucleotides (nt)) the ribosome-binding site (RBS) located near the start codon of the targeted essential genes (Supplementary Data 6), often producing fitness defects greater than 100-fold (Methods and Supplementary Fig. 2) (Fig. 1c,d and Supplementary Fig. 3). Notably, targeting the RBS region of known non-essential genes did not impair growth (Supplementary Fig. 4). Having identified RBS susceptibility to dCas13d targeting, we next aimed to determine whether CRISPRi-ART could inhibit phage infection through RBS targeting.

### CRISPRi-ART enables targeted disruption of phage infection

**Fig. 2:**
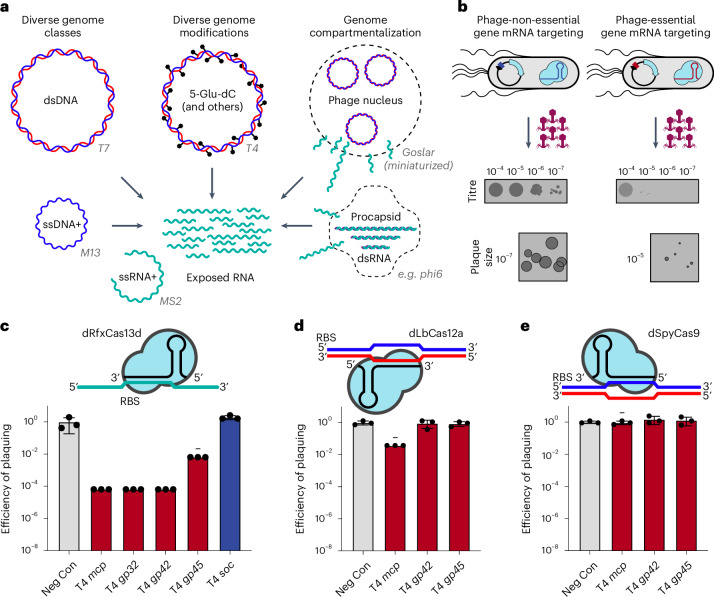
Translational repression provides a simple means to probe phage gene essentiality. **a**, Phage-encoded genome protection strategies. Phage genomes can be constituted by ssRNA+ (green), ssDNA+ (blue), dsRNA (green/purple) or dsDNA (blue/red) molecules (left), heavily modified (centre) or compartmentalized (right) with example phages tested in this study. In all cases, phage mRNA (green) is accessible to Cas13 targeting. Phage genomes not drawn to scale. **b**, Overview of CRISPRi-ART-mediated phage defence. dCas13d expression is driven by the pTet promoter under aTc-inducible control with its crRNA constitutively expressed. Phage infection is inhibited if dCas13d targets an essential phage transcript’s RBS, while infection is productive if the targeted protein is dispensable. Plaque images shown are cartoon illustrations representative of collected data across Figs. 3 and 4. **c**, EOP assays for CRISPRi-ART-mediated phage defence when targeting phage T4 genes. **d**, EOP assays for DNA-targeting dCas12a targeting phage T4 genes. **e**, EOP assays for DNA-targeting dCas9 targeting phage T4 genes. Grey bars: a negative control crRNA; dark red bars, a known T4-essential gene targeting crRNA; dark blue bars: a known T4-non-essential gene targeting crRNA. All EOP values represent the average of 3 biological replicates at 20 nM aTc dCas13d or dSpyCas9 induction or 5 nM aTc dLbCas12a induction. EOP data are presented as mean ± s.d. Minus symbols denote a consistent, ≥4-fold plaque size reduction phenotype if plaques were observed. Source data

To assess whether CRISPRi-ART can discriminate phage gene essentiality through RBS targeting (Fig. 2a)^21^, we applied phage T4 to *E. coli* expressing inducible dCas13d and constitutive crRNA (Methods). We hypothesized that targeting non-essential phage genes would result in no loss of infectivity, while targeting essential phage genes would lead to a reduction in infectivity (Fig. 2b). We found that crRNAs targeting essential T4 genes^25^ consistently reduced the efficiency of plaquing (EOP) by 10^2^–10^4^-fold compared with no inhibition for crRNA targeting a non-essential gene (Fig. 2c and Supplementary Fig. 5). Inhibition occurred only upon dCas13d induction, suggesting that protein knockdown is not due to leaky dCas13d expression (Supplementary Fig. 6). To compare the performance of CRISPRi-ART to previously established double stranded (ds)DNA-targeting CRISPRi tools, we assessed the efficiency of dLbCas12a- and dSpyCas9-mediated phage inhibition. Targeting of essential T4 genes with dsDNA-targeting dLbCas12a (Fig. 2d and Supplementary Fig. 7) or dSpyCas9 (Fig. 2e and Supplementary Fig. 8) resulted in minimal anti-phage activity.

Polar effects, where gene repression yields additional repression of downstream genes in the operon, are known sources of false positive assignments of phage gene fitness using CRISPRi^12^. To explore these effects in CRISPRi-ART, we first targeted essential gene *O* in the well-characterized Lamba P_R_ transcript and observed a large reduction in EOP. Next, we complemented *O* and observed full recovery of EOP (Supplementary Fig. 9), indicating that *O* knockdown does not prohibit expression of downstream essential gene *Q*. This result contrasts with the recent application of dLbCas12a-based DNA-targeting CRISPRi to the same Lambda transcript, which led to the misclassification of non-essential *nin* genes between *O* and *Q* as essential^12^. We conclude that CRISPRi-ART avoids such polar effects, providing a notable advantage in accurately assigning gene essentiality.

### CRISPRi-ART is broadly effective across *E. coli* phage phylogeny

**Fig. 3:**
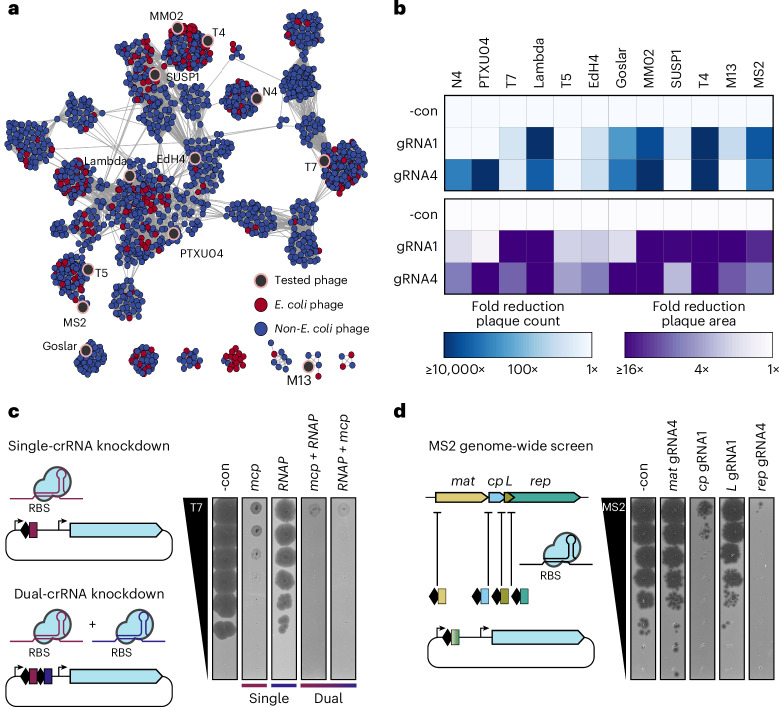
Translational repression is broadly active against phage diversity. **a**, Network graph representation of *E. coli* phages and their relatives^21^. Nodes represent phage genomes connected by edges if they share similarity determined by vContact2 (ref. ^91^). Red and blue nodes represent *E. coli* and non-*E. coli* phages, respectively. Phages assessed here for dCas13d sensitivity are shaded in black. **b**, Anti-phage activity conferred by dCas13d when targeting an essential gene in 12 diverse phages (mean of 3 replicates), scored by EOP reduction (top) or plaque size (bottom). **c**, crRNA multiplexing facilitates more efficient knockdown than component guides, using phage T7 as an example. **d**, Transcriptome-wide knockdown screen in ssRNA phage MS2 using RBS-targeting guides. The best of two guides tested is shown. -con is a non-targeting crRNA control. dCas13d was induced as described in Methods. Source data

To test whether CRISPRi-ART is applicable across diverse bacteriophages, we applied it to 12 coliphages including single-stranded (ss)RNA+, ssDNA+, dsDNA, chemically modified and compartmentalized genomes, as well as temperate, chronic and lytic lifestyles (Fig. 3a and Supplementary Data 8)^13,16–18,20,25–27^. For each phage, we designed two crRNAs (crRNA1 and crRNA4) (Supplementary Fig. 1b) targeting an essential gene encoding the major capsid protein (MCP*)* and measured infection productivity via EOP and plaque size (Fig. 3b). At least one crRNA per phage caused a strong reduction in EOP and plaque size reduction (Supplementary Figs. 10a–j and 11). For a few phages (T7, T5, EdH4, SUSP1 and M13), effective crRNAs caused strong plaque size reduction without a major reduction in EOP. Overall, however, RNA-targeting CRISPRi-ART was far more consistent in its ability to restrict a diverse range of phages compared with DNA-targeting CRISPRi (Supplementary Figs. 12 and 13).

A consistent observation for phages targeted with CRISPRi-ART was a reduction in plaque size when targeting genes expected to affect phage fitness (Fig. 3b and Supplementary Fig. 10j). To test whether repressing two essential genes in these phages would enhance infection inhibition, we employed crRNAs targeting two essential genes either individually or in combination (Fig. 3c). Although each individual crRNA reduced plaque size without EOP reduction, we observed near-complete elimination of plaque formation when using both crRNAs simultaneously. These results suggest that crRNA multiplexing can have synergistic effects.

We next used CRISPRi-ART to analyse the short, 3.6 kb ssRNA genome^28^ of phage MS2, which replicates without DNA intermediates^26^ and thus evades DNA-based tools (Fig. 3b). At least one crRNA targeting each of the four known MS2 genes inhibited infection, while crRNAs targeting inside the coding sequence (CDS) but outside of the susceptible RBS region (Fig. 1d and Supplementary Fig. 1b) on either +sense or −sense RNA strands did not, ruling out direct obstruction of genome synthesis (Fig. 3d and Supplementary Fig. 11a,b). Differences in magnitude of knockdown may reflect a limitation of the crRNAs tested or differential sensitivities to CRISPRi-ART (Supplementary Fig. 11c). Together, these results demonstrate the ability to perform transcriptome-wide knockdown screens in diverse phages (Figs. 2a and 3).

We also tested CRISPRi-ART in four diverse *E. coli* strains sensitive to phage PTXU04, extending applicability to diverse wild-type hosts. CRISPRi-ART achieved substantial reduction of PTXU04 EOP in all four ECOR strains when targeting essential phage genes relative to a non-targeting control crRNA (Supplementary Fig. 14). These results demonstrate that CRISPRi-ART can be successfully applied to genetically diverse *E. coli* strains.

### CRISPRi-ART uncovers diverse superinfection immunity suppressors

**Fig. 4:**
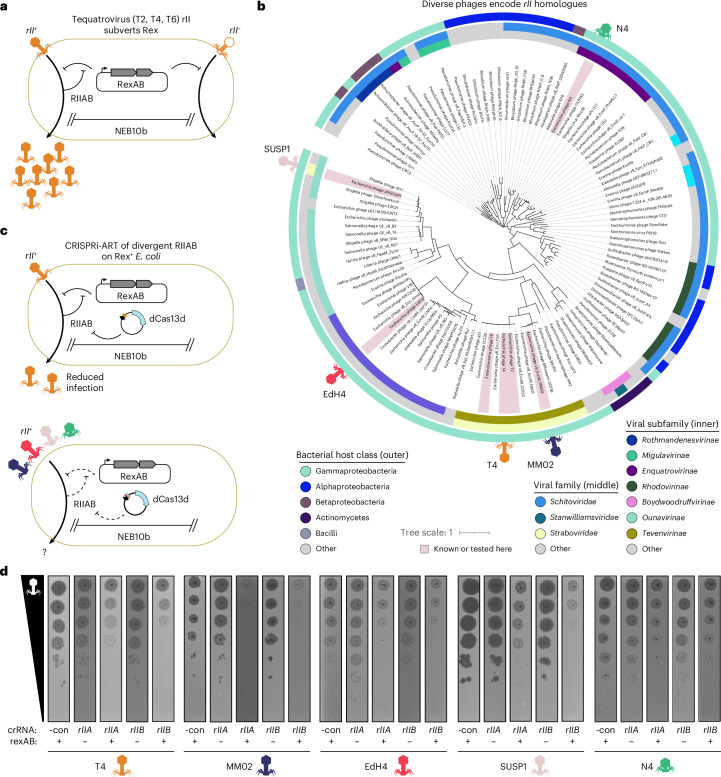
Diverse phages encode divergent rII modules to superinfect lysogens encoding the Rex superinfection immunity system. **a**, Schematic of Rex superinfection immunity defence system and the *rII* counterdefence system. **b**, Phages from 4 subfamilies and 5 genera (phages T4, MM02, EdH4, SUSP1 and N4) encode divergent RIIA and RIIB proteins. RIIA and RIIB homologues were identified from the curated INPHARED viral database^84^. RIIA and RIIB CDSs from each genome were concatenated into a single polypeptide, dereplicated, aligned with MAFFT, assigned phylogenetic structure with FastTree, and visualized with iTOL. Phages whose *rII* genes are known to subvert Rex, or are tested in this study, are indicated with red highlighting. Phages tested here are further denoted with phage symbols. **c**, CRISPRi-ART simplifies investigation of the role of the *rII* module in diverse phages when infecting Rex^+^ *E. coli*. **d**, Representative plaque assays (*n* = 3; Supplementary Figs. 15–20) highlighting the conditional importance of diverse rII homologues. -con is a non-targeting crRNA control. Source data

We next used CRISPRi-ART to investigate the role of the widespread yet poorly understood genetic module rII in subverting RexAB-based superinfection immunity encoded by lambda lysogens. The RexAB system protects against superinfecting phages by inducing membrane depolarization and growth arrest upon detection of phage infection. Phage-encoded RIIA and RIIB proteins counteract RexAB^29,30^, whereas loss-of-function mutants of *rIIA* and *rIIB* render T4 susceptible to RexAB superinfection immunity (Fig. 4a)^31^. Nearly 7 decades after the discovery of these systems^29^, the specificity of this phage–host interaction remains unclear. Given the low sequence identity between diverse *rIIAB* and *rIIAB*-like genes (Fig. 4b), we wondered whether divergent rII systems counteract the Lambda Rex exclusion system or have adapted to preferentially suppress distinct Rex or other immune systems. We confirmed that CRISPRi-ART knockdown of RIIA or RIIB encoded by T4, MM02, EdH4, SUSP1 and N4 phages—spanning four distinct subfamilies and five genera—does not inhibit their infection of *E. coli* lacking Rex, suggesting that these genes are not broadly critical for efficient infection in the absence of Rex-encoding prophages (Fig. 4c,d and Supplementary Figs. 15–20). In contrast, CRISPRi-ART knockdown of RIIA and RIIB reduced EOP during infection of *E. coli* expressing Lambda RexAB, indicating that divergent rII systems (<40% sequence identity) suppress Rex-mediated immunity against diverse phages and thereby license superinfection (Fig. 4c,d and Supplementary Figs. 15–20). Thus, CRISPRi-ART can investigate conditionally important genes involved in the arms race between phages, their hosts and competing mobile genetic elements.

### Transcriptome-scale translational suppression quantifies phage gene fitness

**Fig. 5:**
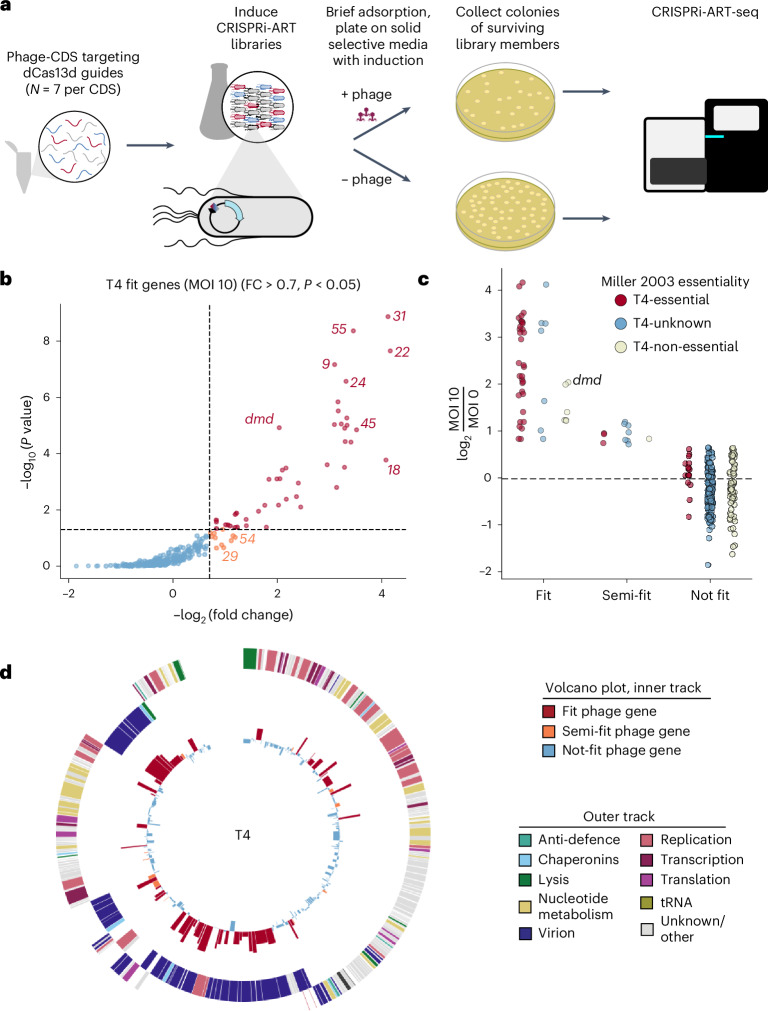
Comprehensive transcriptome-wide gene fitness profiling of phage T4 using pooled CRISPRi-ART. **a**, Experimental library screening pipeline to discover phage genes important for efficient phage infection. **b**, Volcano plot summarizing phage functional genomics screen for phage T4 (273 coding sequences). Representative Fit and Semi-fit genes are highlighted in red and orange, respectively. Thresholds for significance and fitness in volcano plots are shown with a dashed line. **c**, Distribution of Fit, Semi-fit and Not-fit genes in phage T4 binned and coloured by essentiality as reported previously^25^. Antitoxin *dmd*, probably essential in this RnlAB^+^ *E. coli* strain, is labelled. **d**, Phage T4 transcriptome-wide fitness landscape. The outer track displays the phage gene map with genes categorized by function, and the inner track shows fitness measured for each corresponding gene, coloured by Fit (red), Semi-fit (orange) or Not fit (blue). For **b** and **d**, gene fitness is shown as the mean of 3 biological replicates with log_10_-transformed unidirectional K–S *P* value. crRNA fitness is shown as the median of 3 biological replicates. Source data

To implement CRISPRi-ART for phage functional genomics, we targeted each gene in phage T4 with seven individual crRNAs spanning the RBS region (Supplementary Fig. 1) and assessed transcriptome-wide phage-gene fitness in a single, pooled experiment (Fig. 5a). In this assay, crRNAs targeting the RBS of genes essential for phage infection confer a selective advantage to the host and thus increase in relative abundance after treatment, and therefore receive a positive fitness score, an effect we call ‘Fit’. crRNA fitness (and thus gene fitness) was highly consistent across 3 biological replicates (Supplementary Figs. 21 and 22). CRISPRi-ART data were concordant with known gene essentiality in T4 phage, capturing 37 of 50 established T4-essential genes with fold-change alone, alongside 8 genes that are probably essential but not experimentally demonstrated (Fig. 5b,c)^25^. Our results also uncovered several additional Fit genes that are not known to be essential but are important or display host-dependent essentiality (Supplementary Discussion A)^32^. One Fit gene and five Semi-fit genes are of unknown function. The singular Fit gene with unknown function, *frd.2*, is highly conserved across T4-like phages, underscoring unrecognized potential importance in T4 infection. Notably, the 13 T4-essential genes identified as Not fit via CRISPRi-ART displayed higher fitness than Not-fit T4 genes that are non-essential (*P* < 0.05) (Fig. 5c). Future CRISPRi-ART libraries with an increased number of guides per gene may improve sensitivity to identify essential genes with otherwise subthreshold gene fitness (Supplementary Discussion A,E and Fig. 23).

To ensure that fitness quantified in the transcriptome-wide assay corresponded to phage genes rather than toxicity or off-target effects, we validated the pooled assay results with single crRNA plaque assays for ten phage T4 genes (Supplementary Fig. 24). Furthermore, complementation for genes that could be expressed without inducing host toxicity (Supplementary Fig. 25b) recovered EOP and plaque size (Supplementary Fig. 26). Notably, one of these complemented genes, *uvsY.-2*, is of unknown function but is now confirmed to be important for T4 infection. In silico analysis of off-target binding to susceptible phage and *E. coli* RBS regions (Supplementary Fig. 27) revealed minor instances of potential off-target sites when allowing for up to two mismatches in spacer binding, suggesting that off-target binding does not explain false-positive crRNAs. Non-essential gene targeting did not result in a significant fitness effect even when an essential gene was present immediately downstream, ruling out major CRISPRi-ART polar effects (Supplementary Fig. 28).

**Fig. 6:**
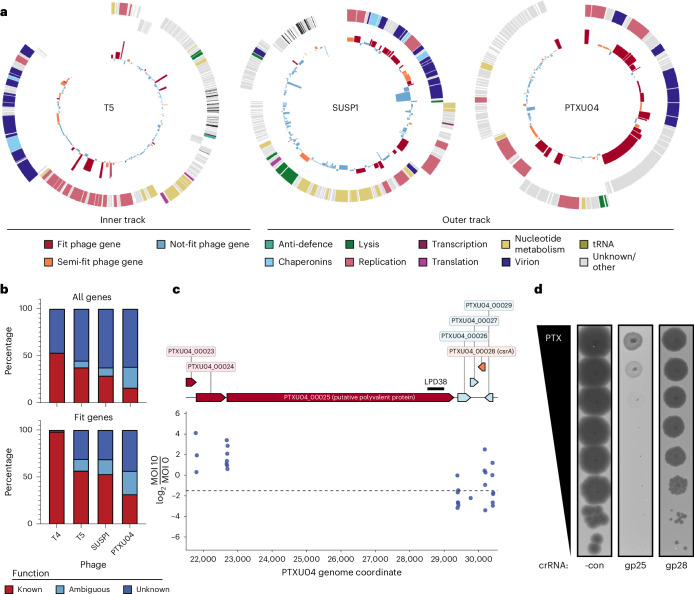
Programmable transcriptome-scale translational suppression unveils the gene fitness landscape of diverse phages. **a**, Transcriptome-wide fitness landscape of phages T5 (162 coding sequences), SUSP1 (138 coding sequences) and PTXU04 (92 coding sequences). The outer track displays the phage gene map with genes categorized by function, and the inner track shows fitness measured for each corresponding gene, coloured by Fit (red), Semi-fit (orange) or Not fit (blue). Gene fitness is shown as the mean of 3 biological replicates. **b**, Distribution of phage gene annotation quality for all phages across the full genome (top) and Fit genes (bottom). **c**, Phage-gene fitness for putative polyvalent protein encoded by *PTXU04_00025* and *csrA* homologue *PTXU04_00028*. crRNA fitness is shown as the median of 3 biological replicates. **d**, Corresponding plaque assays validating pooled gene fitness classification calls with single crRNAs. Targeting Fit gene *PTXU04_00025* (gp25) causes a large EOP reduction, while targeting Semi-fit gene *PTXU04_00028* (gp28) leads to a reduction in plaque size. -con is a non-targeting crRNA control. Representative plaque assays of 3 independent replicates are shown. Source data

In unrelated model phage T5, the top Fit genes uncovered by CRISPRi-ART play roles in early infection (Fig. 6a, and Supplementary Figs. 29a and 30)^16^. Namely, genes important for first/second step transfer (FST/SST) (*A1* and *A2*), a unique feature of T5’s life cycle in which T5 injects ~10 kb of its genome in a discrete stage (FST) from the rest of its genome (SST)^16^, were highly fit (Fig. 6a and Supplementary Fig. 30). To validate the relative importance of lifecycle coordination in phage T5, we investigated the impact of targeting gene *A2*, one of two uncharacterized essential genes involved in T5 SST DNA injection^16,33,34^. A single crRNA targeting *A2* showed strong inhibition of phage T5 (Supplementary Fig. 31), reinforcing the importance of initial infection steps orchestrated by early gene expression.

### Genes of unknown function are central to phage infection

Although most Fit genes identified by CRISPRi-ART in phages T4 and T5 have known functions (Fig. 6b), uncharacterized phages contain many more genes of unknown function. We thus applied transcriptome-wide CRISPRi-ART in non-model phages SUSP1 (ref. ^35^) and PTXU04 (ref. ^36^) (Fig. 6a,b, and Supplementary Figs. 32 and 33). Similar to transcriptome-wide CRISPRi-ART phage screens in T4 and T5, we observed strong enrichment of crRNAs targeting annotated structural phage genes (Figs. 5d and 6a, and Supplementary Discussion C,D). Surprisingly, we found that ~75% of Fit genes have unknown function despite playing important roles in phage infection (Fig. 6b).

After validating 9 CRISPRi-ART-identified SUSP1 genes using single crRNA plaque assays (Supplementary Figs. 25a, 26b and 34), we showed that complementation of 3 of these genes (*gp010*, *gp024* and *gp089*) recovered EOP and plaque size (Supplementary Fig. 26). Notably, these complemented genes in SUSP1 are of unknown function but are now confirmed to be critical for phage infection. In phage PTXU04, we validated all CRISPRi-ART-identified Fit and Semi-fit genes by screening the 2 best-performing crRNAs for each gene using plaque assays (Supplementary Fig. 35). All guides targeting a Fit gene exhibited a severe reduction in EOP or plaque size (Supplementary Fig. 36a); 14 of 16 guides targeting Semi-fit genes conferred a reduction in phage infectivity. In aggregate, these results suggest a non-detectable false discovery rate of Fit genes and a low 12.5% rate among Semi-fit genes. In addition, none of these guides impacted *E. coli* growth in the absence of phage infection (Supplementary Fig. 37). Expressions of genes *PTXU04_00020*, *PTXU04_00022*, *PTXU04_00023*, *PTXU04_00024*, *PTXU04_00025*, *PTXU04_00028* and *PTXU04_00040* were toxic when expressed in *E. coli* or interfered with phage infection, prohibiting complementation experiments for phage PTXU04. These findings underscore the effectiveness of the high-throughput screen in identifying crRNAs that significantly impact phage infectivity.

Across all 4 transcriptome-wide CRISPRi-ART screens, we identified 26 Fit genes of unknown function: 1 in T4 (*frd.2*), 6 in T5 (6 of 17 Fit genes), 7 in SUSP1 (7 of 19 Fit genes) and 12 in PTXU04 (12 of 17 Fit genes) (Fig. 6b and Supplementary Discussion A–D). We used single crRNA targeting to validate one of these, *PTXU04_00025*, a 2,198-amino-acid putative polyvalent protein predicted to play complex roles in subverting host defence during phage infection (Fig. 6a,c,d)^37,38^. We similarly validated the gene *PTXU04_00028*, identified as Semi-fit in this screen and encoding a homologue of the bacterial RNA-binding post-transcriptional regulator CsrA (Fig. 6c). Encoded within diverse mobile elements^39^, the role of CsrA in the phage lifecycle has not been previously tested, although it is proposed to manipulate bacterial hosts by regulating a variety of cellular processes, including immune systems^40^. Knockdown of *PTXU04_00028* caused a substantial reduction in plaque sizes (Fig. 6d), corroborating the transcriptome-wide results.

## Discussion

Here we establish CRISPRi-ART as a foundational phage functional genomics technology that redefines how diverse phage-encoded functions are identified and studied. CRISPRi-ART uses RNA-guided dCas13d to bind and obstruct the RBS of genes, thereby disrupting protein expression and eliciting fitness phenotypes across phylogenetically diverse phages. We employed this approach to uncover previously uncharacterized genes required for phage infection and to dissect several specific aspects of phage–host cell biology.

Harnessing the programmable and multiplexable nature of CRISPRi-ART, we generated genome-wide fitness maps of diverse lytic phages (Supplementary Figs. 22, 30, 32 and 33). Beyond identifying established important genes for well-studied phages, CRISPRi-ART uncovered diverse genes of unknown function with important roles in non-model phage infection. These include genes that are poorly represented in sequence databases and would be omitted from bioinformatic core genome analyses. Comparison of phage gene fitness with comparative-genomic, transcriptomic and predicted protein-folding analyses can catalyse new protein functional discovery. Beyond expanding knowledge of diverse phages’ biology, resolution of gene fitness can rapidly identify dispensable genome regions to inform phage engineering. Coupled with wild-type Cas13 phage gene editing strategies^21^, CRISPRi-ART can guide phage genome reduction for phage therapy or microbiome editing applications^41–44^.

Although phage gene essentiality can be inferred through DNA-binding CRISPRi systems^10,12^, we found CRISPRi-ART to have broader applicability relative to dSpyCas9 and dLbCas12a CRISPRi systems when targeting known essential genes. In parallel with potential future improvements to CRISPRi based on experimental crRNA screening^45^ and native CRISPRi systems^10,46^, CRISPRi-ART offers an alternative, RNA-targeting method for probing gene essentiality in diverse phage genomes. While this manuscript was undergoing review, phage gene knockdown was reported using a non-genetically encoded antisense oligomer strategy in *Pseudomonas* phage PhiKZ^47^. This approach offers a related RNA-targeting approach for genetically intractable hosts but is currently incompatible with pooled assays and involves arrayed, single-gene screening. During individual rounds of culture (plaque assay or liquid infection), this technology yielded limited phage knockdown. Serial passaging amplified modest effects, highlighting how such a strategy may improve poorly performing CRISPRi-ART guides or hosts. Complementary approaches, such as the synthetic construction of genome-wide knockout libraries for phages^48^, offer precise and permanent genetic modifications for dispensable genes. However, challenges associated with assembling large or complex phage genomes and resource-intensive requirements may limit their accessibility and extensibility.

CRISPRi-ART is particularly valuable for the study of essential genes in phage Family *Chimalliviridae* (nucleus-forming phages)^18,49–51^. During infection, these phages sequester their genomes within a proteinaceous phage nucleus, shielding the DNA within from DNA-targeting CRISPRi and genome editing by Cas9 (refs. ^17,20,52^). By targeting mRNA transcripts of such phages, CRISPRi-ART revealed (1) a novel intracellular, phage-generated, membrane-bound compartment during nascent stages of infection (EPI vesicle), (2) the identification and study of a phage nucleus mRNA export protein (ChmC) and (3) a selective phage nuclear protein import machinery (PicA)^49–51^. By disrupting expression of essential phage genes, CRISPRi-ART facilitates the study of other cell states (such as FST during T5 *A2* knockdown) or enigmatic gene functions (such as *PTXU04* gp25 knockdown) during phage infection. Beyond essential gene functions, CRISPRi-ART can identify and study phage-encoded defence-system inhibitors and subversion modules such as Dmd and RIIAB.

We noted several limitations of CRISPRi-ART over the design and course of this study. First, the design of a CRISPRi-ART guide is contingent on correct start codon prediction in phage genomes and thus proximity to its RBS, which may not be straightforward due to highly overlapping CDSs (for example, MS2 *L*), multiple start codons being used for a single gene (for example, T4 *17*), or phage codon recoding^53^. In addition, some guides conferred mild phenotypes such as plaque size changes in plaque assays. One way to improve the signal in such a context would be to leverage multiple rounds of selection, amplifying mild fitness effects^47^. Variability is probably influenced by factors such as crRNA folding, phage biology, phage-gene fitness and host factors. We found that testing two crRNAs (Supplementary Fig. 1) was an effective starting point for the knockdown of a gene of interest. In library experiments, to enhance the likelihood of effective knockdown for each gene, we used seven crRNAs per gene. Post-hoc analysis of crRNA effectiveness did not determine a clear optimal crRNA position relative to the start codon across phages T4, T5, SUSP1 or PTXU04 (Supplementary Figs 38–41). Fitness scores from pooled screening appeared generally proportional to plaque size, indicating that plaque size changes are a form of mild fitness effect (Supplementary Fig. 51). During analysis of transcriptome-wide CRISPRi-ART screens, we observed that crRNA variability yielded a false negative rate, but not a substantial false positive rate, for gene essentiality discovery. Thus, while some essential genes are missed, few non-essential genes are erroneously classified as important. More crRNAs per gene could improve CRISPRi-ART sensitivity during pooled screening (Supplementary Discussion E). The total cost for synthesizing oligonucleotides, library construction and sequencing for a transcriptome-wide CRISPRi-ART screen per phage, including replicates, is approximately US$750, and as short oligo synthesis costs continue to decrease, this expense is expected to decline. Moreover, the entire workflow utilizes standard molecular biology techniques without the need for specialized equipment, making it accessible to laboratories with basic molecular biology capabilities.

The identification of phage phenotypes formed the foundation for modern biotechnology. The simple observation of ‘phage restriction’^54^ led to the discovery and mechanistic understanding of numerous biotechnological tools such as restriction enzymes and CRISPR systems^55–59^. Although over 70% of phage genes are biological ‘dark matter’ with completely unknown functions^4^, CRISPRi-ART provides a versatile and scalable approach for probing the otherwise insurmountable complexity of phage-encoded genetic diversity. Despite substantial genetic and phenotypic differences from *E. coli* K-12, the successful phage gene knockdown in *E. coli* strains ECOR04, ECOR13, ECOR45 and ECOR69 without optimization demonstrates the robustness of CRISPRi-ART, suggesting that it can be readily adapted to a broad range of *E. coli* strains and potentially other bacterial species (Supplementary Fig. 14). The ability to apply CRISPRi-ART across diverse hosts enhances its utility for phage functional genomics and expands the scope of phage–host interaction studies. As CRISPRi-ART identifies new phenotypes across phage diversity, we will discover new functions, uncover new infection strategies and establish new model systems across the phage universe.

## Methods

### Chemicals, reagents and media

All liquid, solid and soft media were prepared with LB Broth Lennox (1% (w/v) tryptone, 0.5% (w/v) yeast extract, 0.5% (w/v) NaCl) and supplemented with antibiotics, inducers and cations as needed and described below. Bottom and top agar were prepared with 1.5% (w/v) and 0.7% (w/v) agar, respectively. LB Broth Lennox was used for routine cultivation of *E. coli* and for experiments in rich medium. Expression of dCas13d was induced by the addition of anhydrotetracycline (aTc; Sigma-Aldrich, CAS 13803-65-1). crRNAs encoded on auxiliary crRNA expression plasmid pBFC1171 were induced using 1 µM crystal violet (for pooled crRNA libraries targeting *E. coli*), while crRNAs encoded on pBFC0984 were expressed from strong constitutive promoter BBa_J23119. Antibiotics were used at a concentration of 34 μg ml^−1^ for chloramphenicol and 100 μg ml^−1^ for carbenicillin. SM buffer (Teknova) was used for phage dilution. All bacterial and phage strains used in this work are listed in Supplementary Data 2.

### Competent cell production

Commercial chemically competent and electrocompetent cells were used when available. Custom chemically competent cells were cultured in ZymoBroth and made competent using Mix & Go buffers (Zymo) according to manufacturer-recommended protocol. For custom electrocompetent cells, overnight *E. coli* cultures in 2×YT medium with appropriate antibiotics were inoculated into the same medium to an optical density (OD) of 0.05 and grown to mid-exponential phase (OD_600_ = 0.4–0.6). Cells were pelleted, washed twice with chilled H_2_O and twice with chilled 10% glycerol, and resuspended in chilled 10% glycerol to achieve a ~300× concentration of the collected culture. Aliquots were immediately frozen at −80 °C.

### Full-plasmid sequencing

All plasmid constructs were sequenced using the full-plasmid sequencing services at the UC Berkeley DNA Sequencing core (Illumina or Oxford Nanopore), Primordium (Oxford Nanopore) or Plasmidsaurus (Oxford Nanopore).

### Phage propagation and scaling

Phages were propagated through commonly used protocols in LB media or LB top agar overlays (0.7%). Unless stated otherwise, phages were propagated on *E. coli* BW25113 (lacI+rrnBT14 ΔlacZWJ16 hsdR514 ΔaraBADAH33 ΔrhaBADLD78 rph-1 Δ(araB–D)567 Δ(rhaD–B)568 ΔlacZ4787(::rrnB-3) hsdR514 rph-1). Phages N4, T4, T5 and T7 were scaled on *E. coli* BW25113. Phage SUSP1 was a gift from Dr Sankar Adhya and scaled on *E. coli* BW25113. Phages EdH4 and MM02 were obtained from the DSMZ culture collection and scaled on *E. coli* BW25113 (DSM 103295 and DSM 29475, respectively). Phage λ cI857 bor::kanR was a gift from Dr Drew Endy and scaled as described previously. Phage MS2 was a gift from Vivek Mutalik and scaled in *E. coli* strain NEB-5α F’Iq (NEB) with 2 mM CaCl_2_. Phage M13 was obtained from ATCC (15669-B1) and was also propagated on NEB 5-α F’Iq genotype cells (NEB) with 2 mM CaCl_2_. All phages were titred through 2 µl spots of 10× serial dilution of phage in SM buffer on *E. coli* BW25113 or NEB-5α F’Iq in a 0.7% top agar overlay.

### CRISPRi-ART vector construction

The *Ruminococcus flavefaciens* Cas13d (RfxCas13d) coding sequence was amplified from addgene plasmid pXR001: EF1a-CasRx-2A-EGFP, a gift from Patrick Hsu (Addgene plasmid 109049; http://n2t.net/addgene:109049; RRID: Addgene_109049). The primary CRISPRi-ART vector pBFC0984 was constructed by assembling a p15A chloramphenicol-resistant backbone with the catalytically deactivated dRfxCas13d coding sequence under transcriptional control of the aTc-inducible pTet promoter, along with a 2xBsaI Golden Gate spacer cloning site for expression of crRNAs from the strong constitutive promoter BBa_J23119. Plasmid pBA556 is similar to pBFC0984 but lacking the crRNA cassette. Plasmid pBFC0984 was used as a spacer entry vector for all individual and dual crRNA constructs, as well as for all phage crRNA libraries. To reduce the likelihood of leaky crRNA expression that could lead to pooled crRNA library bias for libraries targeting the *E. coli* transcriptome, we also built a pBA556-compatible auxiliary crRNA vector pBFC1171 consisting of a low-copy SC101 origin, *bla* ampicillin/carbenicillin resistance marker and a 2xBsaI Golden Gate spacer cloning site for expression of crRNAs from the non-leaky, strong, crystal violet-inducible promoter pJEx^60^. Plasmid pBFC1171 was used as a spacer entry vector for all *E. coli* crRNA libraries. For control crRNA-only samples, plasmid pBFC0843 was constructed and used in place of pBA556 along with the pBFC1171-harboured crRNA library. Plasmid pBFC0843 is dRfxCas13d-null and crRNA-null and possesses a p15A origin, chloramphenicol resistance marker and a pTet promoter without a downstream CDS.

### Individual and dual crRNA cloning

To introduce crRNA spacers into pBFC0984, we employed BsaI-HFv2 (NEB, R3733L) Golden Gate assembly^58^. For individual crRNAs, spacers were designed as two complementary oligonucleotides with 4 bp 5’ overhangs that matched the staggered ends of the BsaI-digested destination plasmid. For dual crRNAs, two pairs of oligos (each encoding one of the two spacers and part of the central direct repeat) were designed to ligate into the backbone in a similar manner, inserting a spacer-repeat-spacer segment. These oligonucleotides were phosphorylated using T4 PNK (NEB) at 37 °C for 30 min and then duplexed at a concentration of 10 μM. Duplex formation involved melting at 100 °C for 5 min, followed by slow cooling to room temperature over a span of 15 min. The PNK-annealed spacer duplexes (100 fmol) served as the insert templates in each Golden Gate reaction. Cloning reactions were subsequently transformed into competent *E. coli* (Mach1-T1R, NEB10B or IG10B) and clones verified by full-plasmid sequencing.

### Cloning and expression of phage gene complementation plasmids

Each complemented phage gene was cloned as a fusion to the C terminus of sfGFP into the 2×BsaI entry vector pBA1328 (low-copy SC101, kanamycin-resistant, tightly regulated pJEx promoter) using BsaI Golden Gate cloning. Removal of the phage gene start codon and fusion to sfGFP shifts the crRNA binding site at the beginning of the phage CDS far downstream of the RBS/translational start site of the sfGFP:phage gene fusion, preventing CRISPRi-ART knockdown of the complemented gene due to its distance from the RBS. This facilitates use of crRNAs targeting the phage-encoded CDS without having to recode the complemented gene. Complementation plasmids were co-transformed with CRISPRi-ART plasmids as needed and maintained with 34 µg ml^−1^ chloramphenicol and 50 µg ml^−1^ kanamycin selection. Phage genes were expressed from complementation plasmids using CV induction of the pJEx promoter^60^ using 200 nM CV. CV concentrations were determined by running a titration series to determine the phenotypically effective concentration.

### Quantifying CRISPRi-ART against phages using plaque assays with individual and dual crRNAs

Bacteriophage plaque assays were performed using a modified double agar overlay protocol as reported previously^21^. Unless stated otherwise, phage assays were performed using DH10b-genotype *E. coli* (NEB, Intact Genomics), DH5α F’ genotype *E. coli* (NEB C2992) (phage M13) or *E. coli* K-12 F+ (Yale CGSC 4401, phage MS2) transformed with a plasmid containing dRfxCas13d, dLbCas12a or dSpyCas9 under pTet control, with a crRNA (or sgRNA in the case of dSpyCas9) under constitutive control (Supplementary Data 1). Cultures were grown overnight at 37 °C and 250 r.p.m. with appropriate antibiotics, and 100 µl of saturated overnight culture was mixed with 5 ml molten LB Lennox top agar supplemented with appropriate inducer (below) and antibiotics. This mixture was decanted onto a corresponding 5 ml LB Lennox + chloramphenicol agar plate to final overlay concentrations of 0.7% (w/v) agar, aTc (variable, below) and 34 µg ml^−1^ chloramphenicol. For dCas13d experiments, the following final concentrations of aTc were used to minimize background toxicity while maintaining phage inhibition: phages T4, MM02, and Lambda (20 nM), phage Goslar (50 nM), and phages EdH4, M13, MS2, N4, PTXU04, SUSP1, T5 and T7 (100 nM). For T4 phage experiments involving dLbCas12a, a lower final aTc concentration of 10 nM was used due to expression toxicity.

In general, no supplementary CaCl_2_ or MgSO_4_ salts were added except for experiments involving phages MS2 and M13, which employed a final concentration of 1 mM CaCl_2_. Overlays were left to dry for 15 min under microbiological flame. For each Cas–crRNA–phage combination, 10× serial dilutions of the appropriate phage were performed in SM buffer (Teknova), and 2 µl of each dilution were spotted onto the top agar and allowed to dry for 10 min. Plaque assays were incubated at 37 °C for 12–16 h. Post incubation, plaques were scanned using a photo scanner and plaque-forming units (p.f.u.s) enumerated. When no plaques but clearings were observed at high phage concentrations, we considered these as ‘lysis from without’ and indicated a lack of productive phage infection^61^. We approximated these EOPs as 1 p.f.u. at the most concentrated dilution of clearing. EOPs were calculated by normalizing the mean of p.f.u.s for a condition to the mean p.f.u.s of a negative control (targeting RFP by default): mean(p.f.u.s condition)/mean(p.f.u.s negative control). All plaque assays were performed in biological triplicate and EOP calculations performed using GraphPad PRISM.

Plaques were further analysed by size in Fiji^62^. Image scale was set to 0 and individual plaques were selected as regions of interest using the full plaque area including the turbid zone. The area of each plaque was calculated. Fold-change for plaque size measurements was calculated as: mean(area_condition)/mean(area_control).

### Oligo pool design and amplification

Oligo pools were designed using a custom script packaged and available for use at https://github.com/BenAdler14/CRISPRi-ART (ref. ^63^). Designed oligo pools were synthesized by Twist Bioscience and designed to encode PCR amplifiable crRNA libraries to be cloned into pBFC0984 or pBFC1171 using BsaI Golden Gate assembly. Oligos containing internal BsaI sites (due to BsaI in the target or a rare BsaI arising when concatenating the final oligo components) were excluded from synthesis to reduce assembly errors. Duplicate oligos encoding crRNAs targeting multicopy or repetitive genomic features were deduplicated before synthesis. The crRNA libraries were synthesized as pools in which each distinct crRNA library was designed to be uniquely amplifiable with an orthogonal primer pair^64^. Each oligo was composed of the following key sequence elements, concatenated in the 5’ to 3’ direction: a 20 nt subpool-specific forward primer, 11 nt encoding the upstream BsaI site and AAAC overhang, 31 nt variable spacer sequence, 1 nt to maintain the starting base of the downstream terminator feature on the crRNA entry vector, 11 nt encoding the downstream TGCT overhang and BsaI site, a 20 nt subpool-specific reverse primer matched to the upstream primer, and a 6 nt arbitrary DNA (Supplementary Fig. 1). Orthogonal primer pairs used for subpool amplification are listed in Supplementary Data 4. Sense RBS control oligos (described below) were synthesized as part of a separate oligo pool from antisense RBS-targeting oligos to prevent amplification problems arising from hybridization of these complementary oligos. Oligo pools were resuspended in Qiagen EB (10 mM Tris, pH 8.5) to 10 ng μl^−1^ and stored at −80 °C when not in use. Subpools were amplified using subpool-specific primers following Twist recommendations and the KAPA HiFi HotStart DNA Polymerase kit (7958889001). Specifically, 25 µl reactions were assembled with 0.5 U KAPA HiFi HotStart DNA Polymerase, KAPA HiFi Fidelity Buffer, 0.3 mM dNTPs, 5 ng oligo pool and 0.3 µM of each subpool-specific primer. The thermocycler programme included an initial melting step for 3 min at 95 °C; 8 cycles of 98 °C melting for 20 s, 50 °C annealing for 15 s, 72 °C extension for 15 s; and a final extension at 72 °C for 1 min. Bioanalyzer confirmed successful amplification of the expected 98 bp products. These PCR products were purified using a DNA Clean and Concentrator-5 kit (Zymo, D4004), eluted with 10 µl milliQ H_2_O and used to assemble crRNA libraries as described below.

### *E. coli* CRISPRi-ART single-nucleotide-tiling crRNA library design

We designed a pooled, single-nucleotide-resolution library with 29,473 crRNAs tiled antisense to 18 *E. coli* BW25113 (accession CP009273) transcripts (Supplementary Data 5). crRNAs were tiled 100 nt beyond the ends of transcriptional start and stop sites when known^65^, or 100 nt beyond the outermost coding sequences when not previously reported. Metadata for the single-nucleotide-tiling crRNA library are shown in Supplementary Data 5. For some of the essential genes on these transcripts, the characteristic RBS-centred fitness defect tract was not observed; we noted that a distinguishing feature of these genes was a markedly lower protein synthesis rate^66^, suggesting that CRISPRi-ART might be more effective at targeting highly expressed proteins. To highlight this susceptible tract, 9 essential genes with marked RBS-centred fitness defect tracts out of the 18 targeted transcripts are plotted in Fig. 1d.

### *E. coli* CRISPRi-ART pooled crRNA library construction

Given the higher diversity of our 1-nt-tiling *E. coli*-targeting library, we used a crRNA library construction approach aimed at maintaining high library coverage and avoiding bias in the cloning and propagation steps. PCR products containing the crRNA libraries were cloned into pBFC1171 using BsaI Golden Gate assembly. To remove undigested entry vector, reactions were subsequently treated with a follow-up digestion and cleanup procedure, consisting of BsaI digestion at 37 °C for 1 h, BsaI heat inactivation at 85 °C for 20 min, Plasmid-Safe ATP-Dependent DNase (LGC Biosearch Technologies) exonuclease treatment at 37 °C for 1 h, Plasmid-Safe heat inactivation at 75 °C for 30 min, and purification using a DNA Clean and Concentrator-5 with 10 μl elution in milliQ H_2_O. High-competency Endura (LGC Biosearch Technologies) electrocompetent cells were electroporated with 1 µl of this product and recovered at 37 °C and 250 r.p.m. for 1 h. A small aliquot was serially diluted and spot plated to count colonies, estimate library coverage and sequence 10 colonies to confirm efficient and diverse crRNA insertion, and the remainder of the recovery stored at 4 °C overnight. On the basis of transformation titres, an appropriate volume of the recovery was plated onto pre-dried bioassay dishes containing LB agar plus carbenicillin, aiming for 100× c.f.u.s over library size and no more than 1,000,000 c.f.u.s on a single bioassay dish. After 14 h overnight growth at 37 °C, colonies were scraped from each bioassay dish into 50 ml LB plus carbenicillin, vortexed thoroughly, pooled if spread across multiple dishes, pelleted by centrifugation and midiprepped with 200 μl milliQ H_2_O elution (ZymoPURE II Plasmid Midiprep kit, D4201) to collect plasmid library DNA (Supplementary Data 1 and 2). To ensure complete removal of undigested entry vector from the plasmid library, 2 µg of DNA was treated with the follow-up digestion and cleanup procedure described above.

Next, experimental strain *E. coli* BW25113 was transformed with either pBA556 to build strain sBFC0264, or pBFC0843 to build strain sBFC0265, and subsequently made electrocompetent in preparation for transformation of crRNA library DNA. The single-nucleotide tiling library was electroporated into sBFC0264 and sBFC0265 (crRNA-only control library) and recovered at 37 °C and 250 r.p.m. for 1 h. Small aliquots of the recoveries were serially diluted and spot plated onto LB agar plus chloramphenicol and carbenicillin to count colonies, estimate library coverage and sequence 10 colonies to confirm maintenance of diverse crRNAs. The remainder of the recoveries were inoculated into 20 ml pre-warmed LB plus chloramphenicol and carbenicillin, grown at 37 °C and 250 r.p.m. until OD_600_ = 0.4–0.8, mixed with equal volume 40% sterile glycerol and frozen at −80 °C as 200 µl 20% revivable glycerol stocks. One glycerol stock for each library was thawed and titred on selective LB agar, indicating high viability after thawing with sufficient c.f.u.s to maintain high library coverage.

### *E. coli* pooled competitive fitness assays

Library aliquots (200 µl) were thawed on ice for 10 min. One aliquot for each library was inoculated into 3 ml LB plus chloramphenicol and carbenicillin in a deep 24-well block and cultivated in a Multitron (Infors) plate shaker at 37 °C and 750 r.p.m. until OD_600_ = 0.5–1.0. At this point, each culture was centrifuged at 4,000 × *g* for 5 min, supernatants aspirated and pellets gently washed in 1 ml M9 base medium (without casamino acids and without a carbon source). This wash procedure was repeated for a total of three times before a final resuspension in 3 ml M9 base medium, and 30 µl of well-mixed cells were inoculated into 3 ml of fresh assay medium, aiming for an initial cell count of 3 × 10^7^ c.f.u.s. All assay media contained the relevant base medium, antibiotics, and 200 nM aTc for dRfxCas13d induction or 1 µM crystal violet for crRNA induction. LB Lennox was used as the base assay rich medium for single-nucleotide tiling (Fig. 1c,d). Competitive growth assays proceeded in 24 deep-well blocks at 37 °C and 750 r.p.m. until OD_600_ = 0.5–1.0 (7–8 doublings), at which point 30 µl of well-mixed culture was subcultured into the same fresh assay medium and the remainder of the culture pelleted and frozen for subsequent CRISPRi-ART-seq of the intermediate time point. The final cultures were cultivated under the same conditions for another 7–8 doublings before collection and freezing at −80 °C for subsequent CRISPRi-ART-seq, totalling 14–16 doublings post induction.

### Phage CRISPRi-ART crRNA library design

The transcriptome-wide phage-targeting crRNA libraries were designed to target the RBS region of all annotated CDSs, using 7 crRNAs coarsely tiled in 5 nt increments antisense to the susceptible RBS region highlighted in Fig. 1d (Supplementary Fig. 1). This enabled comprehensive transcriptome-wide coverage, with at least one guide overlapping both the RBS and start codon of each target gene. For each phage used in this study, gene coordinates and start codon annotations were obtained directly from NCBI, using the accession numbers for phage T4 (NC_000866), T5 (NC_005859), SUSP1 (NC_028808) and PTXU04 (NC_048193).

### Phage CRISPRi-ART pooled crRNA library construction

Given the lower diversity of our phage-targeting CRISPRi-ART libraries, we used a simpler approach for crRNA cloning in which our libraries were transformed into NEB10Beta (the assay strain for T4 and SUSP1, and the cloning strain for T5 and PTXU04). Oligo amplification, Golden Gate assembly, and follow-up digestion and cleanup steps were performed as described above, except that pBFC0984 was used as the entry vector. Commercial electrocompetent NEB10Beta cells were then transformed with 1 µl of plasmid library DNA and recovered at 37 °C and 250 r.p.m. for 1 h. A small aliquot of each recovery was serially diluted and spot plated on LB agar plus chloramphenicol to titre the transformations, and the remainder of the recoveries was stored at 4 °C overnight. Transformation efficiencies were high, producing at least 100× greater c.f.u.s than library size, and sequencing of 10 colonies confirmed high cloning efficiency and crRNA diversity. On the basis of c.f.u. count, an appropriate volume of recovery was plated on standard pre-dried LB agar plus chloramphenicol plates to obtain 100× c.f.u.s over library size, aiming for less than 100,000 c.f.u.s per plate. After 14 h overnight growth at 37 °C, colonies were scraped from each plate into 50 ml LB plus chloramphenicol, vortexed thoroughly, and cultivated in non-baffled shake flasks at 37 °C and 250 r.p.m. After 3 h cultivation, 8 ml of culture was mixed with an equal volume of 40% sterile glycerol and frozen at −80 °C as 1 ml 20% revivable glycerol stocks. The remainder of the culture was pelleted and midiprepped. The T5 and PTXU04 plasmid library DNA samples were further treated with follow-up digestion and cleanup steps, and then electroporated into their final assay strains *E. coli* IG10Beta and BL21, respectively, and validated and stocked as described here for NEB10Beta.

### Phage pooled crRNA competitive fitness assays

Library aliquots (1 ml) were thawed on ice for 10 min, inoculated into 25 ml LB Lennox plus chloramphenicol in non-baffled flasks, and then cultivated at 37 °C and 220 r.p.m. for 30 min. At this point, the cultures were adjusted to 20 nM aTc to induce dRfxCas13d expression. Cultures were grown under induction at 37 °C and 220 r.p.m. for another 1.5–2 h. OD_600_ measurements were used to estimate bacterial c.f.u.s ml^−1^, and ~5 × 10^5^ c.f.u.s (to ensure at least 100× coverage over library size) were set aside for plating. To these tubes containing *E. coli* expressing CRISPRi-ART libraries, phage stocks diluted in SM buffer were added to a multiplicity of infection (MOI) of 0 or 10, mixed, allowed to adsorb for 15 min and then plated on pre-dried LB agar plus chloramphenicol plates containing 200 nM aTc. After overnight incubation at 30 °C, all colonies from a given plate were pooled into 10 ml LB, pelleted and frozen at −80 °C for subsequent CRISPRi-ART-seq.

### CRISPRi-ART-seq

Frozen pellets from fitness experiments were thawed at room temperature after being stored at −80 °C. Plasmid DNA encoding the CRISPRi-ART crRNA library was isolated using a QIAprep Spin Miniprep kit (Qiagen) and quantified using a Qubit High Sensitivity Assay (Thermo) for each sample. A PCR reaction was performed using 50 ng (or a maximum of 10 µl if the sample was below 5 ng µl^−1^) of DNA from each sample in a 50 μl reaction volume, utilizing Q5 HotStart polymerase and pre-barcoded primers. The P1 appending reaction consisted of an initial denaturation at 98 °C for 30 s, followed by 20 cycles of denaturation at 98 °C for 30 s, annealing at 55 °C for 30 s, extension at 72 °C for 30 s and a final extension at 72 °C for 2 min. PCR reactions were cleaned up using SPRISELECT beads according to manufacturer instructions, targeting product sizes of 250 bp (1.8×). Purified PCR products were quantified via Qubit for each sample. Samples were pooled using 20 ng of each sample. The pooled sample was requantified via Qubit and normalized to 15 nM for Illumina sequencing. The final samples were then sequenced on either an Illumina iSeq or Miseq, or pooled on a NextSeq, as specified in Supplementary Data 5.

### crRNA read counting, normalization and fitness calculations

For single-nucleotide-tiling assays against *E. coli* essential gene transcripts, crRNAs were counted with 2fast2q^67,68^ (https://github.com/afombravo/2FAST2Q) using the command ‘python3 -m fast2q -c–m 0–st 30–l 31–ph 0’ and an input.csv file containing a crRNA ID and corresponding spacer sequence for all crRNAs within the counted crRNA library. To account for variations in read depth between individual samples, raw feature counts were internally normalized for each tested crRNA by converting to reads per million. To account for denominator effects, 1 was added to each raw feature count before this normalization. For *E. coli* crRNA log(fold-change (FC)) calculations, crRNA feature counts in test samples were averaged across replicates and divided by corresponding counts in the *T* = 0 condition. Two-way *t*-statistic *P* values were calculated using the scipy.stats module in Python3. FC was log base 2 transformed using the numpy module in Python3. A pairwise comparison of crRNA fitness values indicated strong correlations between all replicates, indicating high reproducibility of single-nucleotide tiling CRISPRi-ART assays in *E. coli* (Supplementary Fig. 2). Fitness values are plotted in Fig. 1c, which shows a single representative gene, including a comparison to crRNA-only controls. Figure 1d presents data for 9 essential genes from the 18 targeted transcripts that exhibited a characteristic fitness defect around the ribosome-binding site region. Supplementary Fig. 3 provides an alternative presentation of Fig. 1d, expanded to a 1,000 bp window around the start codon.

For phage FC calculations, the smallest and largest deciles of crRNA (by read_counts) for MOI 0 or 10 conditions were discarded to eliminate extrema from gene fitness calculations before plating. Remaining crRNA feature counts in test samples were averaged across replicates and divided by corresponding counts in the MOI = 0 condition. To determine whether a gene conferred positive or negative fitness to a phage, the distribution of crRNA FCs for each gene was compared to the distribution of crRNA FCs across the entire phage genome. A unidirectional Kolmogorov–Smirnov test (K–S test) (via the scipy.stats module in Python3) was used to establish whether these distributions significantly differed.

For phage assays, fitness measurements were calculated using a custom conda environment packaging a SnakeMake pipeline and custom Python scripts (https://github.com/BenAdler14/CRISPRi-ART (ref. ^63^)). Briefly, paired-end reads were trimmed and merged using fastp^69^ with custom adapters corresponding to the dCas13d repeat and the crRNA terminator (GTTTCAAACCCCGACCAGTT, ATGCTTGGGCCCGAA). Merged reads were counted using fast2q^67,68^ (https://github.com/afombravo/2FAST2Q) (–m 2–l 30–ph 20–us GGTTTGAAAC–ds ATGCTTGGGC) against a csv encoding the spacers within the library. A quality control metric was imparted on the negative control condition (MOI 0), and the top 10% and bottom 10% most biased crRNAs in the negative control were excluded from analysis. Included reads were normalized to pseudocounts of 1 × 10^6^ per index centred around the mean. For guide fitness (crRNA log(FC)) calculations, crRNA feature counts in test samples were by-sample and divided by the average corresponding counts across the base conditions. For each crRNA feature within a condition, log_2_(FC) values were averaged and two-way *t*-statistic *P* values were calculated using the scipy.stats module in Python3. To determine whether crRNAs targeting a specific phage gene conferred positive fitness against a phage, the distribution of crRNA FCs for each gene was compared to the distribution of crRNA FCs across the entire phage genome. A unidirectional K–S test (via the scipy.stats module in Python3) was used to establish whether a positive benefit was conferred. The fitness threshold for the ‘Fit’ status was determined by the lowest fitness value with a K–S *P*-statistic less than 0.05. ‘Semi-fit’ status was determined by targeted genes with average guide fitness above threshold but below statistical significance (that is, large guide-to-guide variability). A pairwise comparison of crRNA fitness values indicated strong correlations among all replicates, indicating high reproducibility of phage transcriptome-scale CRISPRi-ART assays (Supplementary Fig. 30). For phage T5, the highest correlation was observed between replicates of crRNAs with a fitness value >−1 (Supplementary Fig. 21b inset).

### Phage genome re-annotation

T4, T5, SUSP1 and PTXU04 bacteriophage genomes were functionally annotated through a combination of automated and manual methods. T4, T5 and SUSP1 phages were automatically annotated using genomic annotations from CD-SEARCH^70^ and PHROGS^71^. Because PTXU04 CDSs exhibited limited relation to known proteins and PTXU04 is not in the PHROGS database, PTXU04 was manually annotated with HHpred on the MPI Bioinformatics Toolkit (using PDB_mmCIF70_18_Jun, COG_KOG_v1.0, NCBI_Conserved_Domains(CD)_v3.19, and PHROGs_v4 domain databases)^72^. Each PTXU04 gene was manually assigned an annotation based on either a clear, confident hit (*E*-value < 1 × 10^−5^) or repeated low-confidence annotations (for example, phage tail protein). Additional attempts to annotate remaining hypothetical PTXU04 genes were performed using AlphaFold prediction, followed by structural alignment to PDB and AFDB to limited success^73–76^. All annotations were further manually inspected against known gene content in model phages T4 (ref. ^25^) and T5 (refs. ^16,77^), and non-model phages SUSP1 (via progressiveMauve alignment with default parameters to related *Salmonella* phage FelixO1)^78,79^ and PTXU04 (ref. ^36^). In addition, phage genomes were annotated with phage-defence inhibitors found in T4 and T5 phages^80–83^. If conflict arose during annotation assignment, annotations were prioritized with the following confidence heuristic: literature > PHROGs > CD-SEARCH > HHpred. Any deviations were made on the basis of annotation detail and annotation confidence.

In addition to the above annotations, genes were assigned ‘class’ and ‘annotation quality’ scores. The ‘class’ annotation included the following annotations: anti-defence, chaperonin, lysis, nucleotide metabolism, replication, transcription, translation, tRNA, virion, or unknown/other. ‘Anti-defence’ refers to genes involved in subverting phage-defence systems, including restriction modification systems. ‘Chaperonin’ refers to genes involved in phage virion or protein maturation, but not a structural component of the phage virion. ‘Lysis’ refers to genes involved in lysis, regulation of lysis timing or degradation of cell wall components. ‘Nucleotide metabolism’ refers to genes responsible for nucleotide biosynthesis, degradation, modification and regulation thereof, but not directly a part of replication. ‘Replication’ refers to genes involved in phage replication liberally applied. ‘Transcription’ refers to genes that modulate transcription in either the phage or host genome. ‘Translation’ refers to genes that modulate translation, including genes that modulate RNA or tRNA stability. ‘tRNA’ refers to tRNA genes, but not genes that modify them. ‘Virion’ refers to genes that are structural components or part of the virion produced in infection. ‘Unknown/other’ refers to all other genes encoded in phage. ‘Annotation quality’ was assigned manually based on both confidence, detail and known literature of the annotation and its source content: ‘known’ for genes with known function, ‘ambiguous’ for known genes with ambiguity to substrate or role of the gene, and ‘unknown’ for genes of unknown function.

In general, these were in agreement with PHROG category, with the following exceptions for visualization simplicity: (1) all predicted phage structural components were grouped into the ‘virion’ category including packaged phage proteins; (2) many genes that are critical for phage lifecycle but of unknown molecular function (for example, T5 genes *A1* and *A2*) were grouped into ‘replication’; (3) any gene responsible for assisting folding or assembly was overridden to fall under the ‘chaperonins’ category; (4) genes responsible for anti-phage defence through nucleotide modification were labelled as ‘nucleotide metabolism’; (5) genes with overlapping category functions (for example, RNase H) were labelled with a primary annotation on the basis of literature^25^ and (6) predicted subgenomic mobile elements (for example, homing endonucleases) were assigned ‘unknown/other’ for simplicity. All phage annotations are listed in Supplementary Data 6.

### Analysis of phage CRISPRi-ART-seq

Following CRISPRi-ART-seq processing, phage genes were interpreted for fitness. To identify FC thresholds for Fit and Semi-fit genes, for each phage–MOI condition, we identified the lowest fitness score with a K–S *P* value less than 0.05 on the right tail of the fitness distribution (that is, positively enriched). This value was used as an inclusive FC threshold for fitness. Thus, we determined Fit genes through the following metrics: T4 (10 MOI) (FC > 0.7, *P* < 0.05), T5 (10 MOI) (FC > −3, *P* < 0.05), SUSP1 (10 MOI) (FC > 1.2, *P* < 0.05), PTXU04 (10 MOI) (FC > −1.1, *P* < 0.05). Genes passing FC thresholds but not statistical thresholds were considered to be Semi-fit; such cases are probably reflective of crRNA variability and typically reflected strong fitness of more than one crRNA per gene, but were underpowered to call Fit with significance (Supplementary Fig. 29). Due to the strong selection pressure baseline imposed by phage predation at high MOI, we refrained from interpreting significant negative fitness scores from phage assays in this study.

Volcano plot visualization of phage gene fitness was performed using Python in matplotlib, utilizing FC for the mean of 3 biological replicates and −log_10_-transformed *P* values. Circos plots were generated in Python using pycircos (https://github.com/ponnhide/pyCircos), utilizing phage annotations and mean fitness scores (and Fit/Semi-fit/Not-fit classifications) from CRISPRi-ART-seq as outer and inner tracks, respectively. Genome-wide and genomic-region visualizations were generated using dna_features_viewer (https://edinburgh-genome-foundry.github.io/DnaFeaturesViewer/) and matplotlib in Python. Colouring of phage genes was assigned by CRISPRi-ART-seq fitness classification and crRNA fitness displayed as the median of 3 biological replicates. Median crRNA fitness across the entire phage genome is shown with a dashed line. Comparison of T4 gene fitness scores to T4 essentiality^25^ was performed using gene fitness scores and Fit, Semi-fit and Not-fit classifications for the MOI 10 T4 infection condition using seaborn in Python.

crRNA effectiveness in targeting phage genomes (Supplementary Figs. 38–41) was determined by analysing the crRNAs targeting the top 10 fit genes in phages T4, T5, SUSP1 and PTXU04 (that is, targeting genes that are clearly Fit). To assess absolute and relative crRNA effectiveness, the distribution of crRNA fitness scores and within-gene *z*-score (scipy.stats), respectively, were plotted using seaborn in Python. In addition, guides were interpreted by rank, and the number of top 3 guide RNAs within a gene were plotted by crRNA number using seaborn in Python.

### Construction of phylogenetic tree of RIIA/RIIB homologues

Bacteriophage genomes were acquired from the GenBank-derived INPHARED^84^ database of filtered, curated and annotated sequences (February 2024 accession). Protein sequences from these genomes were searched with MMseqs2 (ref. ^85^) for homologues of RIIA and RIIB using default parameters. Query sequences included RIIA and RIIB sequences from bacteriophages T4, SUSP1, N4, EdH4, MM02, T2 and T6. For each genome where both RIIA and RIIB homologues were found, the CDSs of these genes were concatemerized into a single polypeptide. Concatemerized sequences from the query genomes were included in this collection. Concatemerized sequences were dereplicated with CD-HIT^86^ using an identity cut-off of 0.9. Sequences were then aligned using MAFFT^87^ with default parameters. Gaps in the alignment were removed using TrimAL^88^ with a gap threshold (-gt) of 0.7. Trimmed alignments were converted to a phylogenetic tree with FastTree^89^ using default parameters. Resulting trees were visualized and annotated using iTOL^90^. Viral and host taxonomies in the annotations reflect values in the INPHARED database.

### Statistical analysis

For all pooled crRNA studies and plaque assays, three independent samples were used. Statistical analyses are described in the methods associated with the respective experiments.

### Reporting summary

Further information on research design is available in the Nature Portfolio Reporting Summary linked to this article.

## Supplementary information

Supplementary Information Supplementary Discussions A–E and Figs. 1–42.

Reporting Summary

Supplementary Data 1 Plasmids used in this study.

Supplementary Data 2 Bacterial strains used in this study.

Supplementary Data 3 Phages used in this study.

Supplementary Data 4 Oligos used in this study.

Supplementary Data 5 Processed guide read counts, guide fitness and gene fitness calculations determined in this study.

Supplementary Data 6 Phage-gene annotations used in this study.

Supplementary Data 7 Dataset of all plaque size measurements performed in this study.

Supplementary Data 8 Classification of phages used in this study.

## Source data

Source Data Fig. 1 Fitness data for CRISPRi-ART screens targeting the *E. coli* genome for Fig. 1.

Source Data Fig. 2 EOP data for CRISPRi-ART and CRISPRi targeting bacteriophage T4.

Source Data Fig. 3 EOP and plaque size data used to plot Fig. 3b. The network graph in Fig. 3a is adapted from ref. ^21^.

Source Data Fig. 4 Tree formatted file to plot the tree shown in Fig. 4.

Source Data Fig. 5 Gene fitness data, previous knowledge data, and gene annotation data used for graphs in Fig. 5.

Source Data Fig. 6 Gene fitness and annotation data split by phage. Data are a subset of Supplementary Dataset 5.

Source Data Supplementary Figures Statistical data for supplementary figures where appropriate, compressed into a zip file and organized by supplementary figure number.

## Data Availability

All data supporting the findings of this study are available within the paper and the Supplementary Information. Illumina sequencing data used in this study are deposited in NCBI SRA under BioProject accession PRJNA1196681. Key plasmids used in this study are available through Addgene (https://www.addgene.org/), including the dRfxCas13d entry vector (Addgene plasmid 231992), dRfxCas13d negative control spacer vector (Addgene plasmid 231993) and auxiliary crRNA expression vector (Addgene plasmid 231994). Source data are provided with this paper.

## Code availability

Transcriptome-scale CRISPRi-ART crRNA library design and analysis software developed and implemented in the manuscript are available on GitHub at https://github.com/BenAdler14/CRISPRi-ART (ref. ^63^).
