# Supplementary material for: CRISPRi-ART enables functional genomics of diverse bacteriophages using RNA-binding dCas13d
Source: Nat Microbiol. 2025 Feb 26;10(3):694–709. doi: 10.1038/s41564-025-01935-7 (PMC11879866; doi:10.1038/s41564-025-01935-7)
Supplement: Supplementary file 1 — Supplementary Discussions A–E and Figs. 1–42. [file 41564_2025_1935_MOESM1_ESM.pdf]

nature microbiology

Supplementary information https://doi.org/10.1038/s41564-025-01935-7

CRISPRi-ART enables functional genomics of diverse bacteriophages using RNA-binding dCas13d

In the format provided by the authors and unedited

Table of Contents

Supplementary Discussion

A. Summary of Transcriptome-wide screens in T4
B. Summary of Transcriptome-wide screens in T5
C. Summary of Transcriptome-wide screens in SUSP1
D. Summary of Transcriptome-wide screens in PTXU04
E. Statistical and Practical Considerations for Future CRISPRi-ART Screens

Supplementary Discussion

A. Summary of Transcriptome-wide screens in T4

Our screens identified 46 Fit genes in model phage T4: 1, *3*, *6*, *7*, *8*, *9*, 10, *11*, *12*, *13*, *15*, *16*, *18*, *19*, *20*, *21* (both proteins [1]), *22*, *23*, *24*, *26*, 28, *31*, *32*, *34*, *35*, *36*, *37*, *38*, *39*, 41, *42*, *43*, *45*, *48*, *51*, *52*, *55*, *56*, *67*, *dmd*, *frd.2*, *imm*, *rnh*, *rnlA*, and *uvsX*. Phage T4 is a large dsDNA phage with systematic efforts to fully annotate its genome[2]. These efforts, while still incomplete - approximately one third of its genes remain entirely of hypothetical function - make T4 an ideal model phage to benchmark success of CRISPRi-ART in phage genomes.

Of the 46 Fit genes in model phage T4, we 45 had clear annotations: *1* (deoxynucleoside monophosphate kinase), *3* (tail tube), *6* (baseplate wedge subunit), *7* (baseplate wedge subunit), *8* (baseplate wedge subunit), *9* (baseplate wedge tail fiber protein connector), *10* (baseplate wedge subunit), *11* (baseplate wedge subunit), *12* (short tail fibers), *13* (neck protein), *15* (tail sheath stabilizer), *16* (small terminase subunit), *18* (tail sheath), *19* (tail tube), *20* (portal vertex), *21* (head maturation protease (both proteins [1]), *22* (head scaffolding protein, *23* (major capsid protein), *24* (capsid vertex), *26* (baseplate hub), *28* (gp28 baseplate hub distal subunit), *31* (head assembly chaperone), *32* (single stranded DNA binding protein), *34* (long tail fiber), *35* (tail fiber hinge connector), *36* (tail fiber hinge connector), *37* (long tail fiber), *38* (tail fiber assembly chaperone), *39* (DNA topoisomerase II large subunit), *41* (DnaB Replicative helicase), *42* (dCMP hydroxymethylase), *43* (DNA polymerase), *45* (sliding clamp), *48* (tail tube junction protein), *51* (baseplate hub), *52* (DNA topoisomerase II medium subunit), *55* (late transcription sigma factor), *56* (dCTP pyrophosphatase), *67* (prohead core protein), *dmd* (RnlA anti-toxin), *imm* (superinfection immunity protein), *rnh* (RnaseH), *rnlA* (RNA ligase A), and *uvsX* (UvsX RecA-like recombination protein). An additional Fit gene has no known function: *frd.2*.

In addition we observed 11 Semi-Fit genes in T4, 6 with clear annotations: *29* (baseplate hub and tail length determinator protein), *44* (clamp loader), *47* (endonuclease subunit), *54* (tail tube), *57A* (tail fiber chaperone), and *t* (holin). In addition, we observed 5 T4 Semi-Fit genes with purely unknown function: *30.1*, *30.6*, *mrh.1*, *uvsY.-2*, and *vs.1*.

While much has been learned about the T4 genome since then, Miller et al.,[2] remains the most comprehensive, yet consolidated resource on the T4 genome and the encoded functions within. One particularly useful resource provided in this work is a careful accounting of empirical essentiality and non-essentiality of genes in the T4 genome,

estimating a total of 49 individually essential genes in the T4 genome. Benchmarked against this resource, we recall 33 of these documented essential genes with high confidence and 36 including Semi-Fit genes. Our screens fail to capture fitness for 13 of these genes: *2* (DNA end protector protein), *4* (head completion protein), *5* (tail lysozyme), *14* (neck protein), *17* (Terminase large subunit), *25* (baseplate wedge subunit), 27 (baseplate hub), *33* (late promoter transcriptional regulator), 53 (baseplate wedge subunit), *60* (DNA topoisomerase II), *62* (clamp loader), *68* (prohead core protein), and *e* (endolysin). One insight we drew from this analysis was a limitation of CRISPRi-ART: multiple start sites for the same protein. T4's Large Terminase encoded by gene *17* is encoded by multiple start sites (fig. S20)[3]. Given our model of function, we believe that dCas13d is occluding only one RBS at a time and potentially the Large Terminase is being produced in alternative isoforms from the same transcript. Future work is needed to determine if this is indeed the case. Nonetheless, CRISPRi-ART captures a very complete picture of essentiality in phage T4 when compared to deep literature review.

Our screens capture an additional 12 Fit and 8 Semi-Fit genes beyond what is listed in literature as T4-essential. Some of these genes are ostensibly essential due to clear ties to replication or virion structural components, but not proven empirically: *16* (small terminase subunit), *21* (head maturation protease (both proteins [1]), *28* (gp28 baseplate hub distal subunit), *29* (baseplate hub and tail length determinator protein), *48* (tail tube junction protein), and *54* (tail tube). Some of these genes reflect genes important, but are documented not strictly essential for phage infection: *31* (GroES Chaperone), *32* (single-stranded DNA-binding protein), *rnh* (RNase H), 47 (host-genome phosphoesterase), *rnlA* (RNA ligase A), and *uvsX* (UvsX RecA-like recombination protein). An additional few may reflect a true population-level decrease in fitness, but not essentiality due to effects on superinfection exclusion (*imm*).

One particularly interesting result was T4-encoded *dmd* emerging as a Fit gene. Dmd encodes for an antitoxin inhibiting *E.coli* -encoded toxin RnlA, whose activity otherwise degrades RNA. Inhibition of RnlA by Dmd stabilizes T4 mid-infection, conferring a strong benefit by Dmd[4]. While Dmd is non-essential in the context of RnlA- infection, DH10b harbors an intact copy of RnlA, suggesting that CRISPRi-ART screens can identify defense system inhibitors encoded in lytic phage genomes. Notably anti-defense genes that were not relevant to the strain used in this screen do not appear phage fit[5–7].

Finally, we highlight the inclusion of 11 genes of hypothetical function that appear Fit (*frd.2*) or Semi-Fit (*30.1*, *30.6*, *mrh.1*, *uvsY.-2*, and *vs.1*). These genes represent

potential inroads into new, previously unidentified biology such as host-encoded defense system inhibition encoded by phage T4.

B. Summary of Transcriptome-wide screens in T5

Our screens identified 17 Fit genes in model phage T5: *A1*, *A2*, *C1*, *D3*, *D5*, *D11, D15*, *dmp*, *pol*, *sciB*, *T5.026*, *T5.047*, *T5.089*, *T5.107*, *T5.114*, *T5.148*, and *T5.156*. While phage T5 is among the original model type phages[8], it remains comparatively uncharacterized relative to model phages such as T4. While key features of T5 genetics remain a mystery, mechanisms in phage-defense and phage-defense-subversion mechanisms have renewed interest in this phage.

Of the 17 Fit genes in model phage T5, 9 had clear, known annotations: *dmp* (5′-Deoxyribonucleotidase), *A1* (SST DNA injection essential protein), *A2* (SST DNA injection essential protein), *C1* (holin), *D11* (putative single strand DNA binding protein), *D15* (exonuclease), *pol* (DNA polymerase), *sciB* (terminase small subunit), and *T5.148* . Additionally 2 putative DNA-binding regulatory proteins, *D3* and *D5* appeared Fit as well. The remaining 6 Fit genes (*T5.026*, *T5.047*, *T5.089*, *T5.107*, and *T5.114*) are of entirely unknown function.

One of the most striking results when screening across the T5 genome is the clear enrichment of crRNAs targeting pre-early genes: dmp, A1, and A2. Following phage adsorption to the cell, T5 injects the first 10kb of its genome (encompassing 17 genes) into the cytoplasm, beginning a phase called first step transfer (FST) [9]. Following expression and activity of A1 and A2 (the only strictly essential genes in this region), T5 injects the remainder of its genome by an unknown mechanism in a phase called second step transfer (SST). Our results corroborate the relative importance of A1 and A2 activity. Additionally host-genome degrading protein Dmp, shows fitness in our screens, deviating from established non-essentiality of this gene [9]. The enrichment of crRNAs targeting host degradation genes in this region likely helps facilitate survival of the host, albeit to a lesser extent than targeting A1 and A2.

We additionally identified 15 Semi-Fit genes in T5, 12 of which had clear annotations:
D2 (helicase), *D12* (sbcD endonuclease), *D18-19* (tail length tape measure protein), *D20-21* (major head protein), *ligA* (DNA ligase), *oad* (long tail fiber), *rnh* (Rnase H), *T5.076* (anti-retron protein), *T5.139* (distal tail protein), *T5.146* (tail terminator), *T5.150* (head maturation protease), *T5.155* (terminase large subunit), *T5.011* (hypothetical protein), *T5.062* (hypothetical protein), and *T5.123* (hypothetical protein).

It should be noted that outside of the pre-early region in T5, we noticed a decrease in CRISPRi-ART consistency between different crRNAs targeting the same genes, especially at high MOIs. This means that many clearly-essential genes in T5 are omitted from Fit classification. For instance, in the core region of T5 between D2 and D20-21,

many likely essential genes fall in the Semi-Fit category due to crRNA to crRNA variability (D18-D19 tape measure protein is a clear example). However inclusion of Semi-Fit genes introduces weaker-than-normal precision in analysis of the T5 genome too. Relative to other phages in this study, direct interpretation of Semi-Fit genes should be approached with extra caution during analysis of the T5 genome due to this decreased precision.

Outside of the pre-early region, we observed a clear enrichment of crRNAs targeting replication and virion proteins in the region between *D2* and *oad* (region from 63,000-111,000). In particular, of the 18 replication genes annotated as replicative in T5 that were not called Fit or Semi-Fit were *D2*, *ligB*, *pri*, and *D13*. In addition, only 6 of 16 virion proteins were not Fit or Semi-Fit. Of these, *T5.151* (hoc-like protein, known dispensable), *T5.036* (an Ambiguous annotation), *ltf* (L-tail fiber), and T5.136 (L-tail fiber attachment) are likely truly non-essential for infection of O-antigen deficient *E.coli* [10–12].

Finally, we observed 5 proteins of completely unknown function or roles in T5 with Fit status: T5.047. T5.084, T5.089, T5.090, and T5.114. Some of these proteins are among the most fit in our screens in T5 (T5.047 and T5.114). T5.114 colocalizes with another Fit gene, *D3*, suggestive of a confident, but unknown role of this two-gene cassette in the context of T5 infection. The gene *T5.047* is localized nearby, but not within, a region of known dispensability in T5 [13]. These proteins might be a reflection of to-be-discovered mechanisms of how T5 interacts with *E.coli* DH10b or missing, non-structural components of the T5 lifecycle.

C. Summary of Transcriptome-wide screens in SUSP1

Our screens identified 19 Fit genes in non-model phage SUSP1: a*gp002*, a*gp006*, a*gp007*, *agp010*, *agp012*, *agp013*, *agp014*, *agp016*, *agp018, agp023*, *agp024*, *agp027*, *agp046*, *agp048*, *agp054*, *agp055*, *agp061*, *agp089*, and *agp130*. As a non-model phage, SUSP1 has relatively little known about its biology beyond being a "superspreader phage" [14]. In addition, SUSP1 displays significant homology to *Salmonella* phage FelixO1, a *Salmonella*-specific, but broad host range phage within species, recently identified to have complex genetic interactions with its host [15–17]. Of the 138 coding annotations for SUSP1, 114 ultimately derive from FelixO1 via Mauve whole genome alignment and may reflect on this classical phage's biology [15,18].

Of the 19 Fit genes, 10 had clear- and 2 had ambiguous- annotations: a*gp002* (portal protein), a*gp006* (tail protease), a*gp007* (major capsid protein), a*gp012* (tail sheath), a*gp013* (virion structural protein), a*gp014* (tail assembly chaperone), a*gp016* (tape measure protein), a*gp023* (baseplate protein), a*gp046* (DNA polymerase), a*gp48* (tail protein), a*gp055* (exonuclease), a*gp61* (ribonucleoside-diphosphate reductase large subunit (NrdA)). These proteins primarily reflect structural features of the phage, but also include nucleotide metabolism (a*gp061*/*nrdA*) and replicative functions (a*gp046* and a*gp055*). The remaining 5 Fit genes are of entirely hypothetical function.

We additionally identified 8 genes of Semi-Fitness: a*gp001* (terminase large subunit), a*gp020* (baseplate spike protein), a*gp021* (baseplate wedge subunit), a*gp022* (baseplate wedge subunit), *agp038* (hypothetical protein), *agp081* (RIIA lysis inhibitor), *agp134* (hypothetical protein), *agp137* (hypothetical protein). 5 of these had clear annotations and 3 with no known function.

Between the 27 Fit or Semi-Fit genes a very high percentage of the intuitively-essential SUSP1 genome was captured. Of the 19 predicted structural components (17 of them directly measured confirmed present in FelixO1 virions [15]), 10 appear Fit or Semi-Fit for SUSP1 and only miss proteins with ambiguous (*agp003*, *agp004*, *agp008*, *agp009*, *agp017*, *agp111*, *agp125*) or adsorptive functions that might have functional redundancy and thus dispensable against *E.coli* DH10B (*agp025*, *agp026*). 2 of 5 of the identifiable chaperonins appeared Fit as well as 4 of 6 identifiable replication genes (missing *agp040* encoding DNA Ligase and *agp051* encoding DNA primase). While SUSP1 is a non-model phage, our results corroborate that the high-level understanding of the important structural and replicative components of SUSP1 (and by extension, FelixO1) is well-understood.

Notably absent from our Fit or Semi-Fit genes are genes related to lysis by SUSP1 (with the exception of semi-fit gene *AVU07_agp081*). Strangely, this is a consistent theme during the study of lysis in *Ounaviridae*, which have unique phenotypes surrounding lysis. SUSP1 (and FelixO1) release plasmids into the environment upon lysis of the infected cell. Model *Ounavirus*, FelixO1 (nonetheless SUSP1) to date lacks an identifiable holin [15], the primary essential component for cellular lysis by double-stranded DNA phages [19]. The lysis aspect of the *Ounavirus* lifecycle remains a mystery.

Across the four phages assessed in this study, SUSP1 was the only phage to show strong phenotypes when targeting genes responsible for *de novo* nucleotide biosynthesis (*agp061*) [20]. Potentially, this too is related to the "superspreader" phenotypes documented with SUSP1 [14]. Because SUSP1 doesn't digest the host genome or plasmids during the course of infection, SUSP1 might be comparatively reliant on *de novo* nucleotide biosynthesis during replication. Although these genes have homologs in phage T4 (*frd*, *td*, *nrdA*, and *nrdB*), these genes do not show up as fit in T4. Potentially, one of the trends in the evolution of phages with larger genomes is the ability to acquire "building blocks" via multiple strategies.

Finally, our collective results identify 9 genes in SUSP1 of completely hypothetical function displaying Fit or Semi-Fit phenotypes: *agp010*, *agp024*, *agp027*, *agp038*, *agp054*, *agp089*, *agp130*, *agp134*, and *agp137*. On average, these genes were smaller on average and were dispersed across the SUSP1 genome. Occasionally, as the case for *agp130*, these genes were among the top scoring in our SUSP1 Fit screens.

To identify candidates that may reflect essential host-adaptation to *E.coli* relative to *Salmonella* we looked for genes that were Fit in SUSP1, but were not in the FelixO1 genome. We identified 3 such genes: *agp038* and *agp137*. One particularity is the location of *agp137*, which occur amidst the tRNA-encoding region of SUSP1. Potentially these genes in the auxiliary genome of *Ounaviridae* reflect an adaptation to host defenses that target tRNAs.

D. Summary of Transcriptome-wide screens in PTXU04

Our screens identified 17 Fit genes in singleton phage PTXU04: *gp01*, *gp07*, *gp08*, *gp09*, *gp10*, *gp11*, *gp16*, *gp17*, *gp19*, *gp20*, *gp23*, *gp24*, *gp25*, *gp38*, *gp40*, *gp41*, and *gp92*. PTXU04 was originally reported as a "singleton phage" and we find it to have only marginal similarity to other isolated phages: in our network graph, PTXU04 only shares homology of one of its proteins with neighboring phages (Fig. 4A) [21]. Because of this distant relation to other studied phages, annotation of PTXU04 is comparatively poor and is predominantly encoded by genes of unknown function. All functional annotations generally need to be approached with skepticism.

Of the 17 fit genes identified in this study, only 5 had confidently inferable function with hhpred: *gp01*, *gp07*, *gp10*, *gp38*, and *gp41*, encoding small terminase subunit, portal protein, putative major capsid protein (*mcp*), single-stranded DNA binding protein (SSB), and replicative DNA helicase, respectively. An additional 5 genes had ambiguous but weak function: *gp9*, *gp11*, *gp16*, *gp17*, and *gp92*, encoding putative head scaffolding protein, virion protein, and endonuclease respectively. Another gene, *gp25*, harbored a "Large Polyvalent Domain 38" (LPD38) annotation at its C-terminus[22]. While its predicted size and intrinsic disorder suggest that it's a polyprotein, no other domains of confidence were inferrable in this large protein. Polyvalent proteins play an intriguing role in phage biology and are predicted to play enigmatic roles in the phage-host arms race [22].

We additionally identified 17 genes of Semi-Fitness: *gp03*, *gp04*, *gp05*, *gp18*, *gp22*, *gp28*, *gp30*, *gp34*, *gp37*, *gp42*, *gp44*, *gp55*, *gp60*, *gp68*, *gp74*, *gp81*, and *gp83*. Only 2 had confidently inferable functions with hhpred[23]: *gp04* and *gp28*, encoding a tail fiber protein and a CsrA homolog, respectively. Another 2 genes encoded inferred structural components, but lacked confident annotations: *gp3* and, *gp18*.

In aggregate, these screens reveal a reliable trend with the PTXU04 genome. Consistent with the model phages, ostensible essential genes in PTXU04 appear to colocate in several regions of the genome: *gp1*- *gp11*, gp16-*gp25, and gp34-gp44*. However, we observed notable omissions of many of the expected essential genes encoded within. For instance, *gp02* and *gp30* encode likely essential genes encoding large terminase and lysozyme encoded within otherwise Fit regions that display subthreshold fitness. Others, such as *gp30* might merely exhibit "true" crRNA to crRNA variability, as its maximal fitness guide performs better, limiting their fitness in this context.

Finally, maybe the most curious outcome of these screens is the clear existence of a highly Fit series of genes from *gp16*-*gp25*, the vast majority of which have no identifiable function. This result suggests a probable deviation for PTXU04 from other phages in terms of its lifecycle and might provide a clear path for characterization of the enigmatic LPD domains of bacterial viruses.

E. Statistical and Practical Considerations for Future CRISPRi-ART Screens

For phage functional genomic screens, we designed phage transcriptome-wide libraries by synthesizing seven guides per gene as outlined in Supplementary Figs. 1. We found that between biological replicates guide RNA fitness was very consistent with Spearman correlation ≥ 0.8 across all replicates except for phage T5 (Supplementary Fig. 30). For phage T5, only the most fit crRNAs showed such reproducibility. Because of the strong fitness observed with *dmp*, *A1* and *A2* targeting crRNAs consistent with their established roles for T5 infection progression, we believe that low reproducibility outside of the best performing guides is due to T5's ability to rapidly degrade the host nucleic acid.

Seven guides per gene were chosen to balance biological variability, pooled synthesis costs and appropriate sample size to be sensitive to conclude that these guides represent Fit genes from a one-sided Kolmogorov–Smirnov (KS) test relative to the entire guide fitness distribution. Supplementary Fig. 32a shows a theoretical analysis of minimum KS statistic for determining a Fit gene for "k" guides from a distribution of crRNA fitness containing "N" total guides, where N=k*num_genes. Fewer than five guides per gene dramatically lowers the sensitivity for concluding a true positively Fit gene. Increased number guides could improve sensitivity, especially at higher levels of confidence. Additionally, we found that this level of sensitivity was invariant across an increasing number of phage genes above ten genes in a genome (Supplementary Fig. 32b). Given that phages T4, T5, SUSP1 and PTXU04 each have over 90 genes, we felt that seven guides per gene was appropriate for this experimental setup.

In practice, due to variability during library outgrowth and bottlenecking during plating (Methods) fewer than 7 guides per gene factored into gene fitness calculations (Supplementary Fig. 32c,e,g,i, Methods). This would lower the sensitivity for these genes, incurring an increased risk of False Negatives. To estimate this impact, we simulated what would have happened if only "k" guides were sampled per gene from the library from our library experiments (Supplementary Fig. 32d, f, h, j) with 50 repetitions. The number of estimated Fit genes with high confidence decreases and variability increases, especially at k≤4, consistent with decreasing sensitivity in Supplementary Fig. 32a. Given that 10s of genes per phage were represented with 4 or fewer guides, some true Fit genes were likely missed but called Semi-Fit in our analyses.

Given that additional sources of biological and technical variability exist in screens, we would broadly recommend a slightly higher number of guides per gene of 10 for increased robustness to sources of experimental variability and guide variability. At the time of writing, crRNA libraries for T4, T5, SUSP1 and PTXU04 can be synthesized as

oligonucleotide pools at under $500 USD per phage, decreasing in cost with the number of phages investigated. The corresponding plasmid libraries can be readily prepared using straightforward steps accessible to any standard molecular biology lab. Specifically, a single PCR and one Golden Gate cloning reaction yield sufficient colony-forming units for 100× library coverage on a single petri dish. We feel that such a recommendation is both economically feasible for future work while bolstering against sources of variability.

Supplementary Figures

Supplementary Fig. 1 | crRNA design for CRISPRi-ART RBS tiling.
Supplementary Fig. 2 | Replicate Correlation Plots of Single Nucleotide Tiling CRISPRi-ART for E. coli Essential Gene Transcripts.
Supplementary Fig. 3 | Expanded Fitness Plots of Single Nucleotide Tiling CRISPRi-ART for E. coli Essential Gene Transcripts.
Supplementary Fig. 4 | Impact of CRISPRi-ART Induction on E. coli Growth.
Supplementary Fig. 5 | T4 CRISPRi-ART plaque assays.
Supplementary Fig. 6 | Assessment of CRISPRi-ART Induction in Plaque Assays.
Supplementary Fig. 7 | T4 dLbCas12a CRISPRi plaque assays.
Supplementary Fig. 8 | T4 dSpyCas9 CRISPRi plaque assays.
Supplementary Fig. 9 | Lambda CRISPRi-ART Polarity Assessment Plaque Assays.
Supplementary Fig. 10 | CRISPRi-ART plaque assays targeting phage RBS with dRfxCas13d.
Supplementary Fig. 11 | MS2 CRISPRi-ART Plaque Assays.
Supplementary Fig. 12 | dLbCas12a CRISPRi Assays Across Phage Diversity.
Supplementary Fig. 13 | dSpyCas9 CRISPRi Assays Across Phage Diversity.
Supplementary Fig. 14 | CRISPRi-ART Inhibits Phage PTXU04 infection in Diverse E. coli ECOR strains.
Supplementary Fig. 15 | T4 CRISPRi-ART RIIA/B Plaque Assays.
Supplementary Fig. 16 | MM02 CRISPRi-ART RIIA/B Plaque Assays.
Supplementary Fig. 17 | EdH4 CRISPRi-ART RIIA/B Plaque Assays.
Supplementary Fig. 18 | SUSP1 CRISPRi-ART RIIA/B Plaque Assays.
Supplementary Fig. 19 | N4 CRISPRi-ART RIIA/B Plaque Assays.
Supplementary Fig. 20 | CRISPRi-ART RIIA/B Plaque Assay EOPs.
Supplementary Fig. 21 | Transcriptome-Wide CRISPRi-ART Replicate Correlation Plots.
Supplementary Fig. 22 | Transcriptome-wide CRISPRi-ART Fitness for Phage T4.
Supplementary Fig. 23 | Theoretical and Post-Hoc Analysis of CRISPRi-ART Library Design.
Supplementary Fig. 24 | T4 CRISPRi-ART Single crRNA Validation Plaque Assays.
Supplementary Fig. 25 | T4 and SUSP1 CRISPRi-ART Complementation Replicates.
Supplementary Fig. 26 | T4 dCas13d CRISPRi plaque assays.
Supplementary Fig. 27 | Phage CRISPRi-ART Off-Target Analysis.
Supplementary Fig. 28 | Assessment of CRISPRi-ART Polarity in T4.
Supplementary Fig. 29 | T5, SUSP1, and PTXU04 Transcriptome-Wide Gene Fitness Summaries.

Supplementary Fig. 30 | Transcriptome-wide CRISPRi-ART Fitness for Phage T5.
Supplementary Fig. 31 | Phage T5 Gene Fitness in Pre-Early Genes.
Supplementary Fig. 32 | Transcriptome-wide dCas13d-fitness for phage SUSP1.
Supplementary Fig. 33 | Transcriptome-wide CRISPRi-ART Fitness for Phage PTXU04.
Supplementary Fig. 34 | SUSP1 CRISPRi-ART Single crRNA Validation Plaque Assays.
Supplementary Fig. 35 | PTXU04 CRISPRi-ART Validation Plaque Assays.
Supplementary Fig. 36 | CRISPRi-ART Validation Plaque Assays EOPs.
Supplementary Fig. 37 | Assessment of Phage-Targeting Fit crRNAs on *E. coli* Growth.
Supplementary Fig. 38 | crRNA Fitness Distributions for Top 10 Fit Phage T4 Genes.
Supplementary Fig. 39 | crRNA Fitness Distributions for Top 10 Fit Phage T5 Genes.
Supplementary Fig. 40 | crRNA Fitness Distributions for Top 10 Fit Phage SUSP1 Genes.
Supplementary Fig. 41 | crRNA Fitness Distributions for Top 10 Fit Phage PTXU04 Genes.
Supplementary Fig. 42 | Guide fitness versus plaque size for T5 D20-21 (mcp).

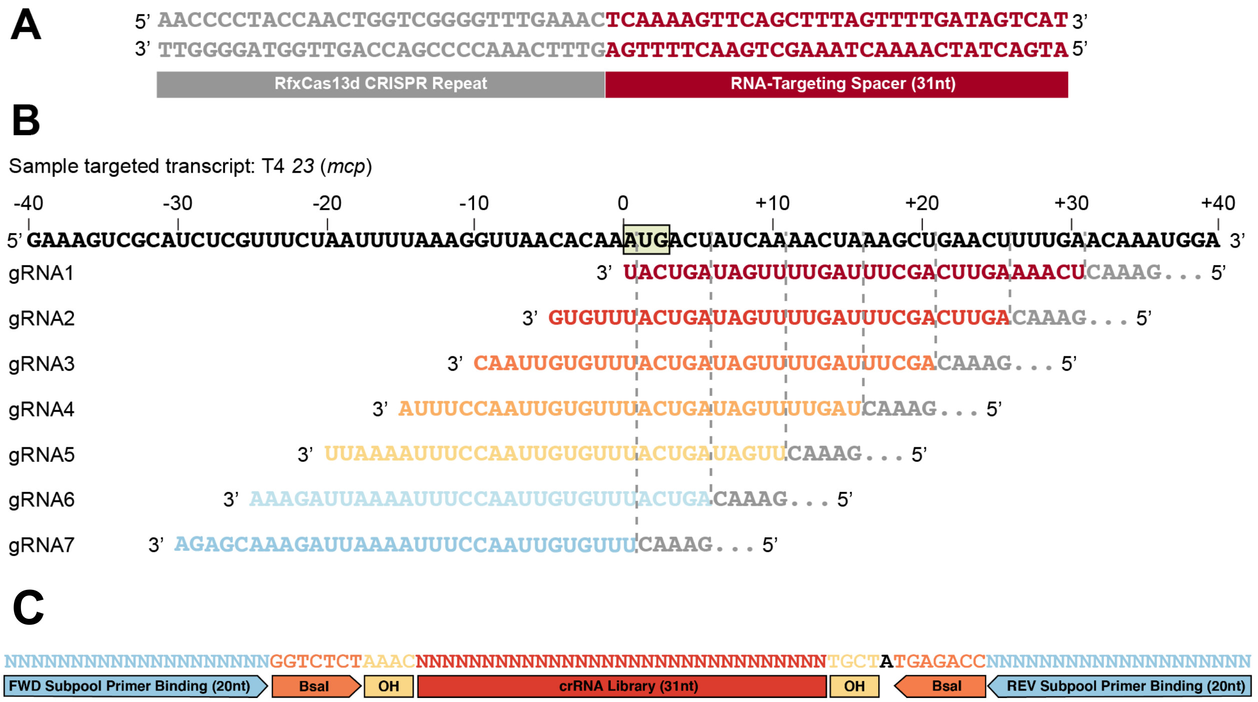

Supplementary Fig. 1 | crRNA design for CRISPRi-ART RBS tiling. All examples in Supplementary Fig. 1 refer to crRNAs (referred to here as guide RNAs, or crRNAs) that target Phage T4 *23* (*mcp*) **a,** Example T4 *mcp* targeting crRNA1 encoded in dRfxCas13d plasmid consisting of a CRISPR repeat (gray) and a 31 nucleotide spacer (red, reverse complement of targeted transcript) **b,** For RBS-tiling designs, crRNAs are designed using 31 nucleotide spacers targeting every 5 nucleotides along a transcript from +31 to +1 starting positions for crRNA1 to crRNA7 respectively. Mature RNA targeting sequences are highlighted in rainbow. Partial CRISPR repeats are shown in gray. Targeted transcript is shown in black with the start codon highlighted. **c,** All oligos in the crRNA oligo pools encode two 20 nt subpool-specific priming sites to facilitate selective amplification of distinct subpools, two BsaI sites with appropriate overhangs to clone into oligo library entry vectors, and 31 nt crRNAs.

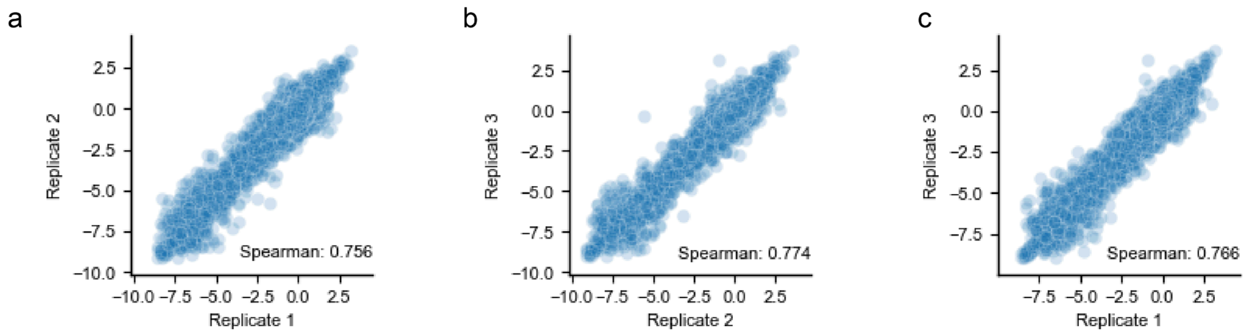

Supplementary Fig. 2 | Replicate Correlation Plots of Single Nucleotide Tiling CRISPRi-ART for *E. coli* Essential Gene Transcripts. Pairwise comparison of crRNA fitness between replicates of pooled single nucleotide tiling CRISPRi-ART assays targeting *E. coli* essential gene-encoding transcripts indicates high reproducibility. **a-c** provide pairwise comparisons between each combination of three replicates. For all plots, crRNA fitness Spearman correlation coefficient is provided between replicate experiments.

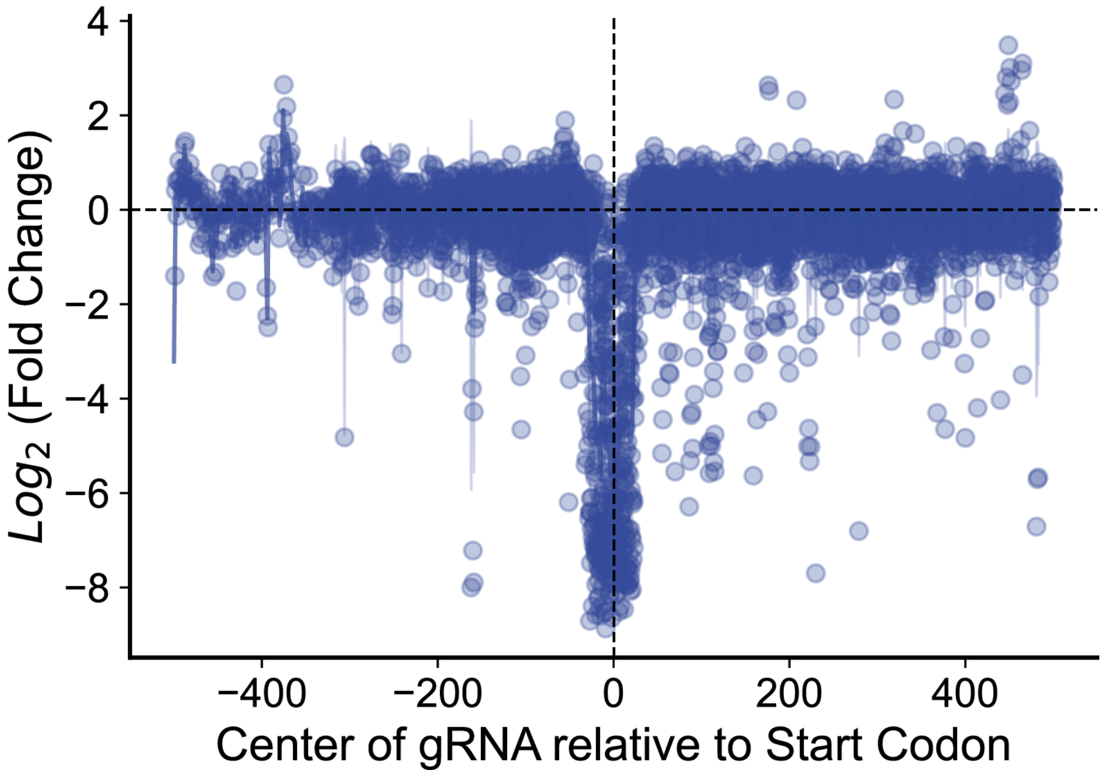

Supplementary Fig. 3 | Expanded Fitness Plots of Single Nucleotide Tiling CRISPRi-ART for *E. coli* Essential Gene Transcripts. The observed Log_2(fold change) of crRNA abundances targeting the RBS region of 9 essential genes is compiled into a single plot, where points represent the center of the crRNA spacer. Highlighted is an expanded 1000 bp surrounding the start codon relative to Fig. 1d. Average of values at each nucleotide position is plotted across the region, along with a 95% confidence interval.

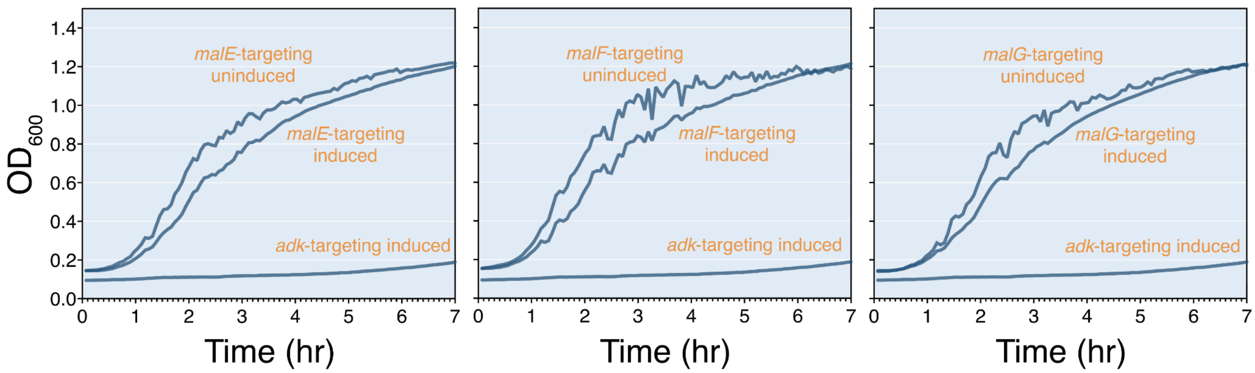

Supplementary Fig. 4 | Impact of CRISPRi-ART Induction on *E. coli* Growth. Growth curves of *E. coli* expressing CRISPRi-ART targeting non-essential genes *malE*, *malF*, and *malG* under uninduced and induced conditions. Induced guide targeting *E. coli* essential gene *adk* is provided as a positive control demonstrating CRISPRi-ART-mediated growth repression. Cultures were grown in the absence of inducer (aTc) (uninduced) and in the presence of 200 nM aTc (induced). Optical density at 600 nm (OD_{600}) was measured at 5 min intervals to monitor bacterial growth over time. Experiments were performed in triplicate, and data are presented as mean of triplicates.

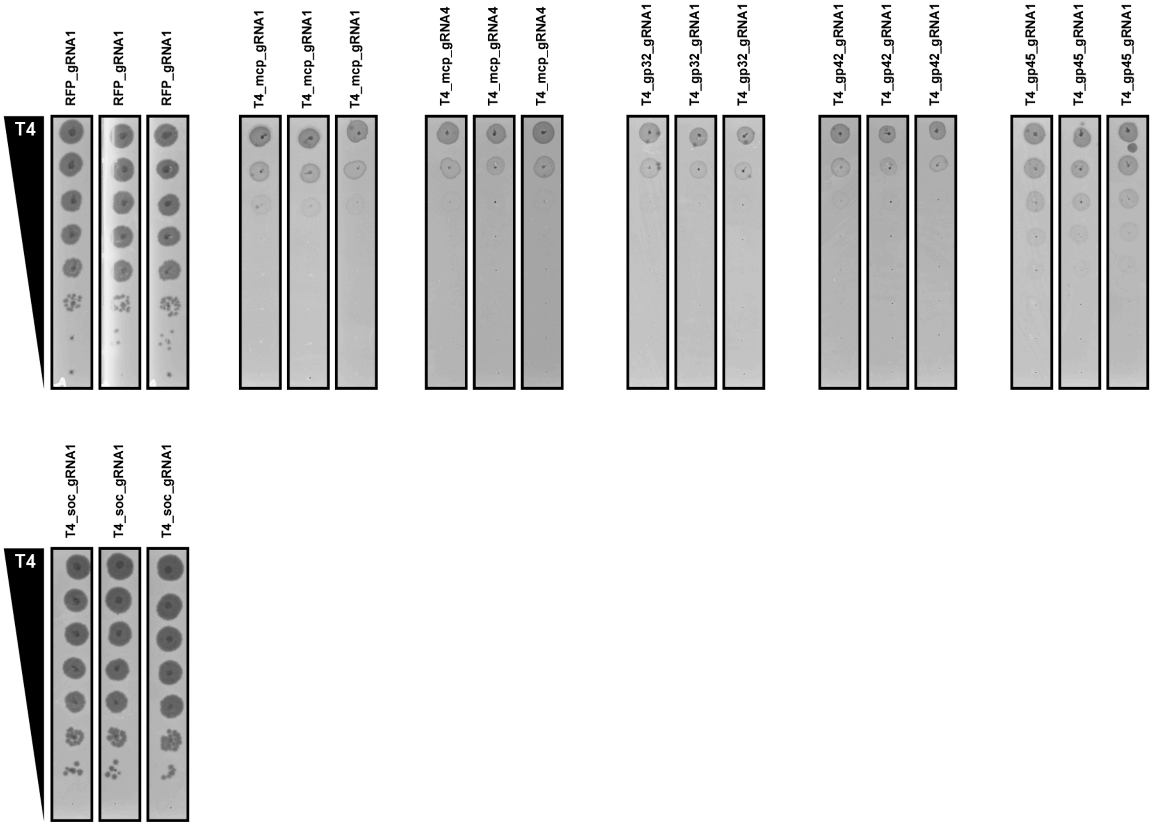

Supplementary Fig. 5 | T4 CRISPRi-ART plaque assays. Plaque assays for CRISPRi-ART-mediated phage defense when targeting phage T4 RBS with dRfxCas13d, data reported in Fig. 2c and Fig. 3b heatmap. A guide targeting RFP is provided as a negative control. dRfxCas13d experiments were expressed using +20nM aTc. Data shown are for 3 biological replicates.

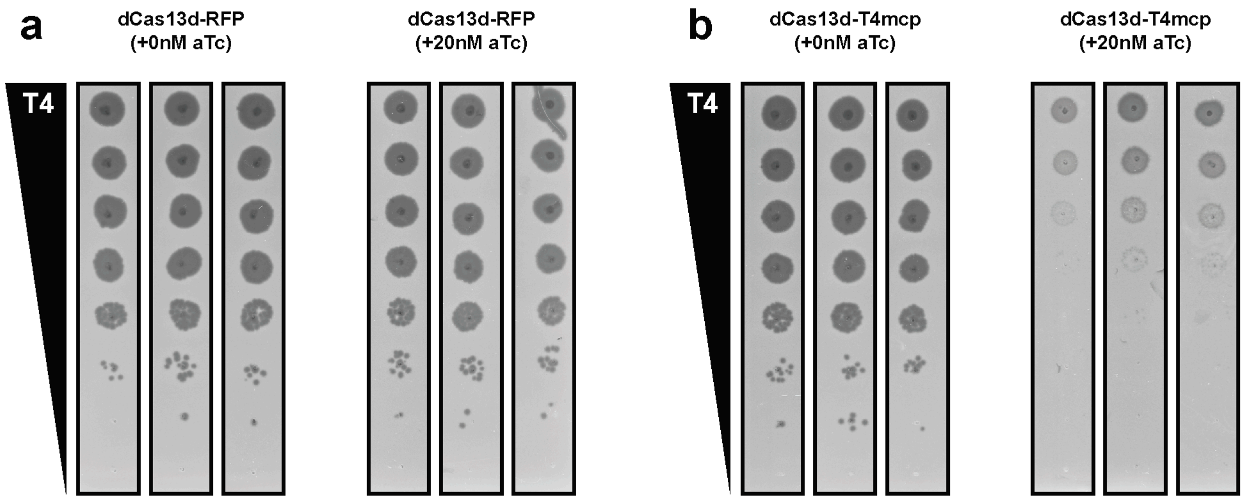

Supplementary Fig. 6 | Assessment of CRISPRi-ART Induction in Plaque Assays. Triplicate T4 plaque assays in cells expressing CRISPRi-ART targeting **a,** RFP (negative control) or **b,** T4 mcp. dCas13d effect on phage infection inhibition was compared between uninduced (+0nM, left) and induced (+20nM aTc, right) conditions.

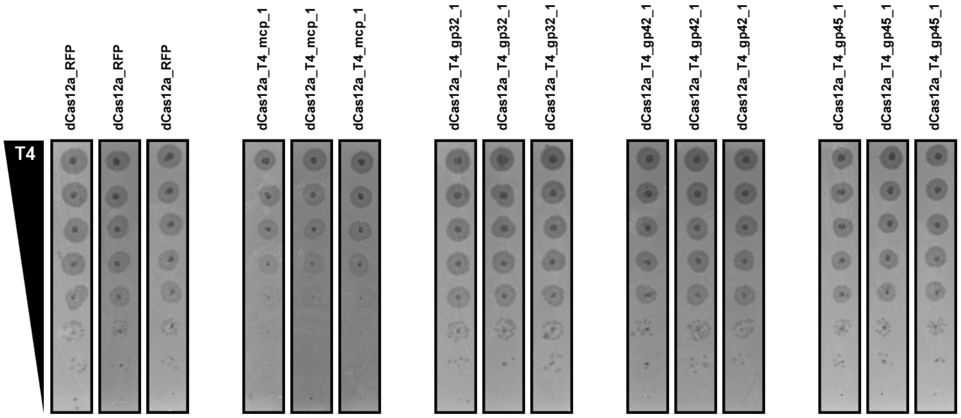

Supplementary Fig. 7 | T4 dLbCas12a CRISPRi plaque assays. Plaque assays for CRISPRi-mediated phage defense when targeting phage T4 CDS with dLbCas12a, data reported in Fig. 2d. A guide targeting RFP is provided as a negative control. dLbCas12a experiments were expressed using +10nM aTc. Data shown are for 3 biological replicates.

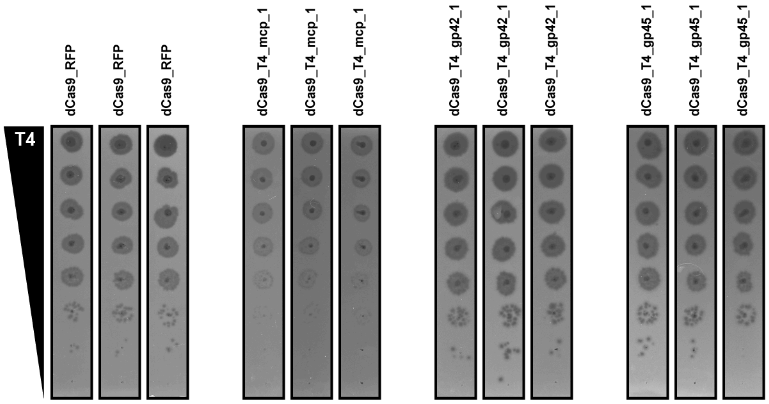

Supplementary Fig. 8 | T4 dSpyCas9 CRISPRi plaque assays. Plaque assays for CRISPRi-mediated phage defense when targeting phage T4 CDS with dRfxCas13d, data reported in Fig. 2e. A guide targeting RFP is provided as a negative control. dSpyCas9 experiments were expressed using +20nM aTc. Data shown are for 3 biological replicates.

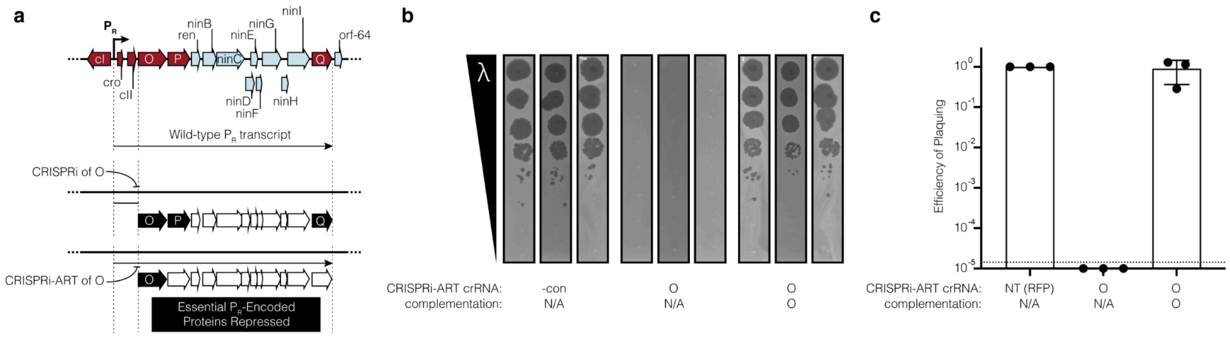

Supplementary Fig. 9 | Lambda CRISPRi-ART Polarity Assessment Plaque Assays. a, Schematic of phage Lambda transcript P_R, depicting essential (red) and non-essential (blue) genes in its operon. Comparison of the effect of *O*-targeting CRISPRi transcriptional repression (middle) and CRISPRi-ART translational repression (bottom), where CRISPRi polarity in this transcript has been shown to repress downstream essential genes[24]. In contrast, we show that CRISPRi-ART of *O* does not exhibit polar repression of essential genes *P* and *Q* by fully recovering infectivity through *O* complementation. **b,** Plaque assays for CRISPRi-ART-mediated inhibition of phage Lambda infection when targeting the RBS of Lambda *O*, with or without *O* complementation. A guide targeting RFP is used as a negative control (-con). dRfxCas13d was induced using 20 nM aTc, and *O* was induced with 50 nM crystal violet. Data shown are for 3 biological replicates. **c,** EOP calculations based on b, where the dashed line reflects the limit of detection. Error bars are presented as mean +/- SD.

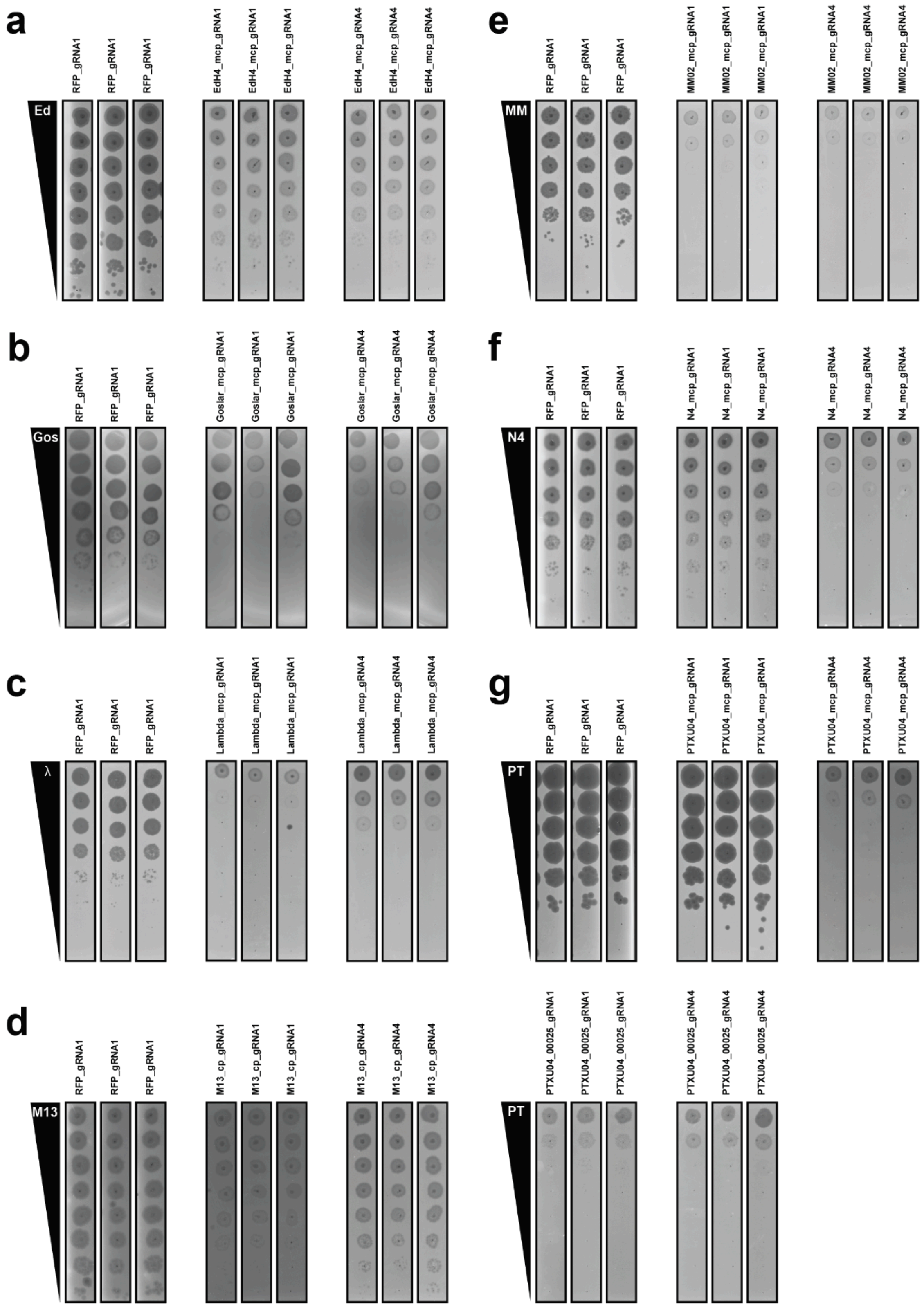

Supplementary Fig. 10 | CRISPRi-ART plaque assays targeting phage RBS with dRfxCas13d (continued below).

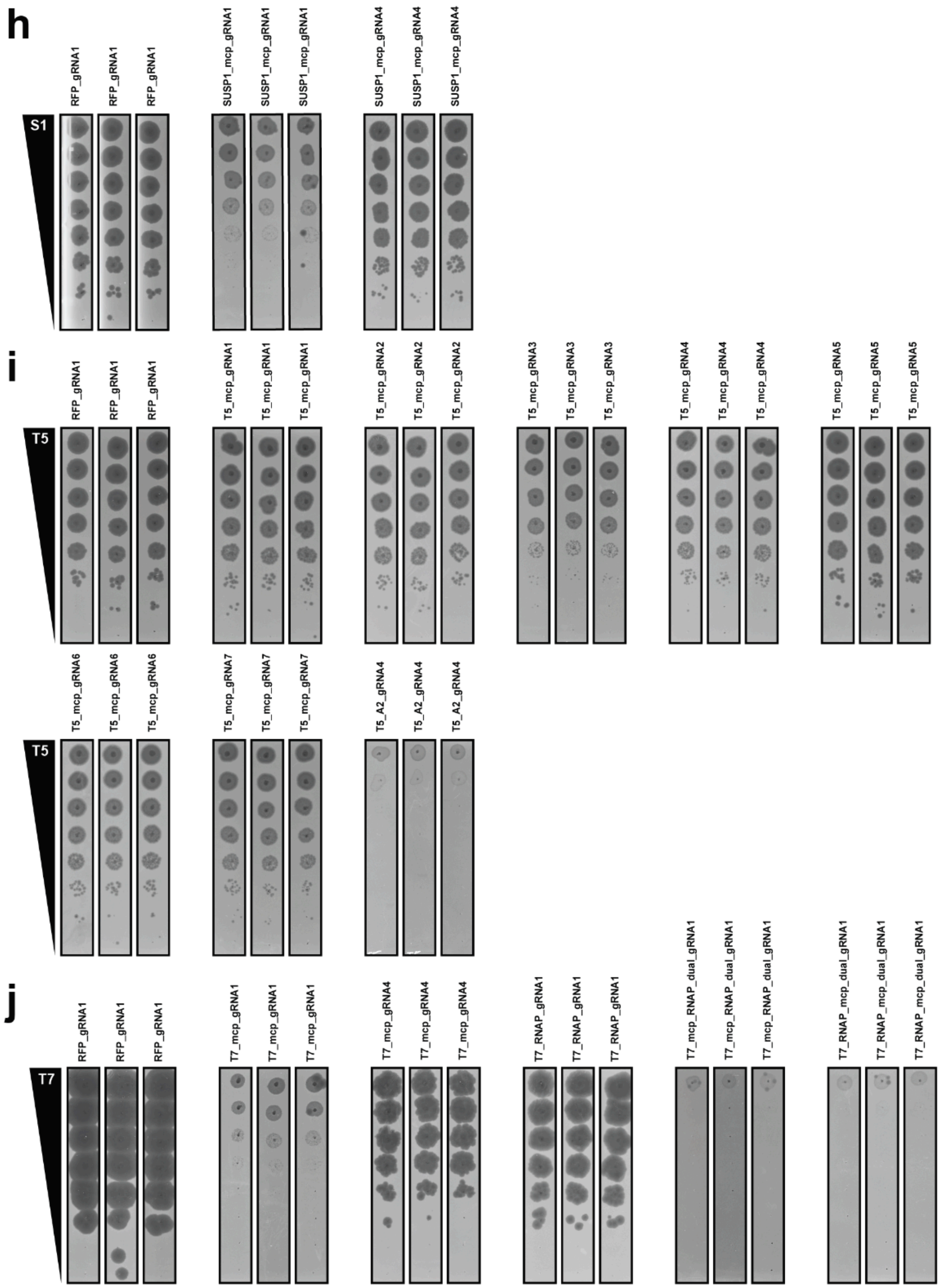

Supplementary Fig. 10 | CRISPRi-ART plaque assays targeting phage RBS with dRfxCas13d.

Plaque assays demonstrating CRISPRi-ART-mediated phage defense by targeting the ribosome binding sites (RBS) of various phages using dRfxCas13d. A guide targeting RFP serves as a negative control in all cases. dRfxCas13d expression was induced with anhydrotetracycline (aTc) at concentrations specified for each phage. Data are shown for three biological replicates and correspond to those reported in Fig. 3b heatmap and other figures as indicated.

Panels (a–j) represent results for phages EdH4 (a), Goslar (b), Lambda (c), M13 (d), MM02 (e), N4 (f), PTXU04 (g), SUSP1 (h), T5 (i), and T7 (j), respectively. For phages EdH4, Goslar, N4, PTXU04, SUSP1, T5, and T7, dRfxCas13d was expressed using +100 nM aTc. Phages Lambda and MM02 were induced with +20 nM aTc. Phage M13 experiments included +100 nM aTc and +1 mM $CaCl_2$. Data for PTXU04 are also reported in Fig. 6d; full data for T5 are in Supplementary Fig. 29; data for T7 are also presented in Fig. 3c.

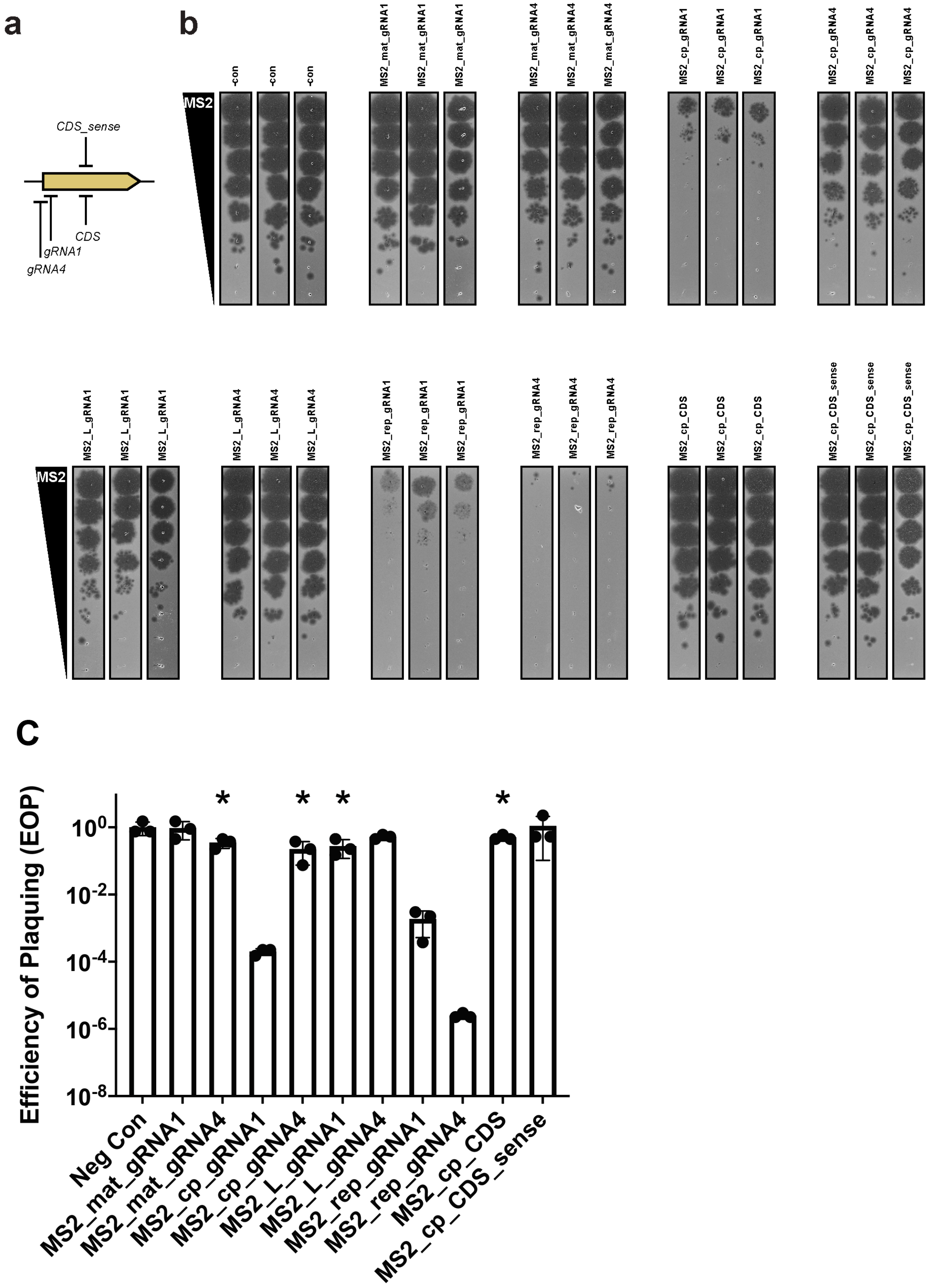

a
b
CDS_sense
CDS
gRNA1
gRNA4
-con
MS2_mat_gRNA1
MS2_mat_gRNA4
MS2_cp_gRNA1
MS2_cp_gRNA4
MS2
MS2_L_gRNA1
MS2_L_gRNA4
MS2_rep_gRNA1
MS2_rep_gRNA4
MS2_cp_CDS
MS2_cp_CDS_sense
C
Efficiency of Plaquing (EOP)
10^0
10^-2
10^-4
10^-6
10^-8
*
Neg Con
MS2_mat_gRNA1
MS2_mat_gRNA4
MS2_cp_gRNA1
MS2_cp_gRNA4
MS2_L_gRNA1
MS2_L_gRNA4
MS2_rep_gRNA1
MS2_rep_gRNA4
MS2_cp_CDS
MS2_cp_CDS_sense

Supplementary Fig. 11 | MS2 CRISPRi-ART Plaque Assays. a, Overview of guides used for MS2 relative to a MS2 coding sequence (CDS). crRNA11 and crRNA44 refer to crRNA nomenclature described in Supplementary Figure 1 and used in the rest of the paper. Because MS2 is a ssRNA(+) phage with dsRNA intermediates, we employed antisense ("CDS") and sense ("CDS_sense") guides targeting the middle of the CDS as controls for replication inhibition for CRISPRi-ART. **b,** Plaque assays for CRISPRi-ART-mediated phage defense when targeting phage MS2 RBSs with dRfxCas13d, data subset reported in Fig. 3d. When targeting phage genes outside of the susceptible RBS region (Fig. 1D, Supplementary Fig. 1) but inside the CDS, no phage defense is detected. **c,** EOP measurements of MS2 plaque assays. Guides that led to a significant reduction in plaque size compared to the non-targeting crRNA control (Neg Con) as measured by a two-way ANOVA and are indicated with an asterisk (adjusted p-value < 0.0001) reported only when there is not a significant reduction in EOP. MS2 CRISPRi-ART experiments were expressed using +100nM aTc and +1mM $CaCl_2$. Data shown are for 3 biological replicates. Error bars are presented as mean +/- SD.

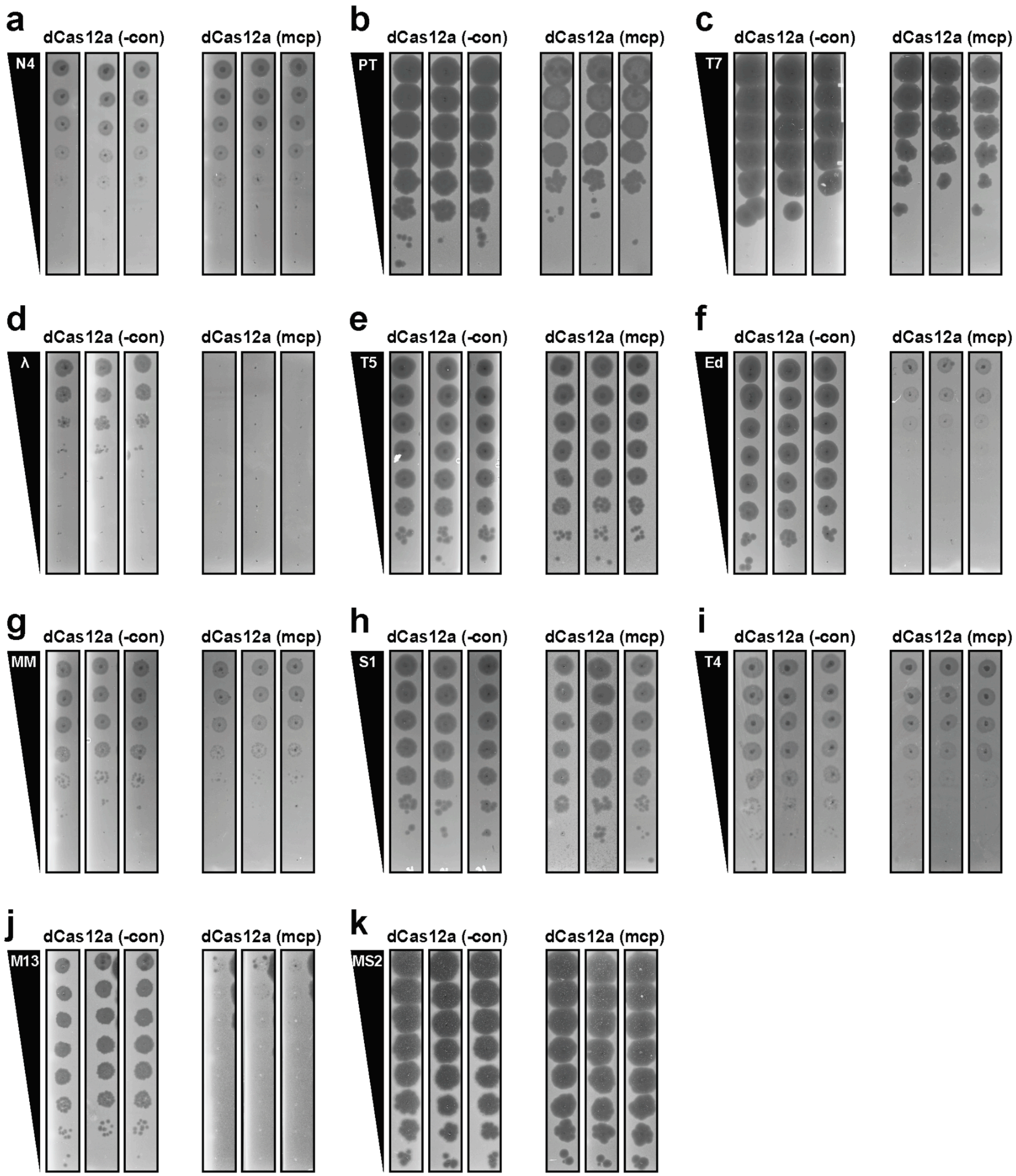

Supplementary Fig. 12 | dLbCas12a CRISPRi Assays Across Phage Diversity. Plaque assays for dsDNA-targeting CRISPRi when targeting phage major capsid proteins (right) versus a non-targeting crRNA (left) with dLbCas12a for phages **a,** N4, **b,** PTXU04, **c,** T7, **d,** Lambda, **e,** T5, **f,** EdH4, **g,** MM02, **h,** SUSP1, **i,** T4, **j,** M13, **k,** MS2. A guide targeting RFP is provided as a negative control. dLbCas12a experiments were expressed using +5nM aTc (maximal expression before toxicity observed). Assays for

phages M13 and MS2 were supplemented with +1mM $CaCl_2$. Data for T4 is also presented in Supplementary Fig. 4. Data shown are for 3 biological replicates.

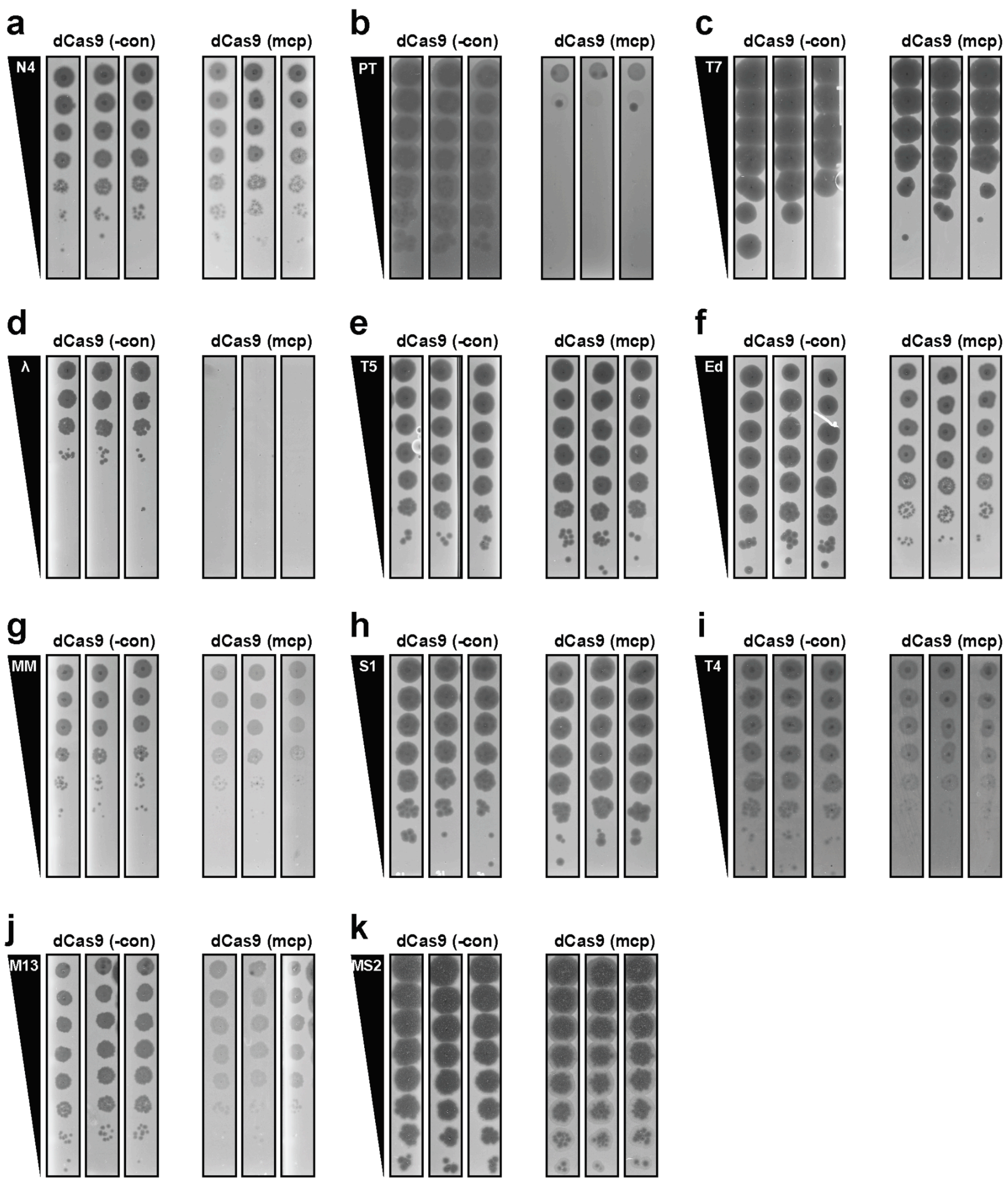

Supplementary Fig. 13 | dSpyCas9 CRISPRi Assays Across Phage Diversity. Plaque assays for dsDNA-targeting CRISPRi when targeting phage major capsid proteins (right) versus a non-targeting crRNA (left) with dSpyCas9 for phages **a,** N4, **b,** PTXU04, **c,** T7, **d,** Lambda, **e,** T5, **f,** EdH4, **g,** MM02, **h,** SUSP1, **i,** T4, **j,** M13, **k,** MS2. A guide targeting RFP is provided as a negative control. dSpyCas9 experiments were expressed using +20nM aTc (maximal expression before toxicity observed). Assays for

phages M13 and MS2 were supplemented with +1mM $CaCl_2$. Data for T4 is also presented in Supplementary Fig. 5. Data shown are for 3 biological replicates.

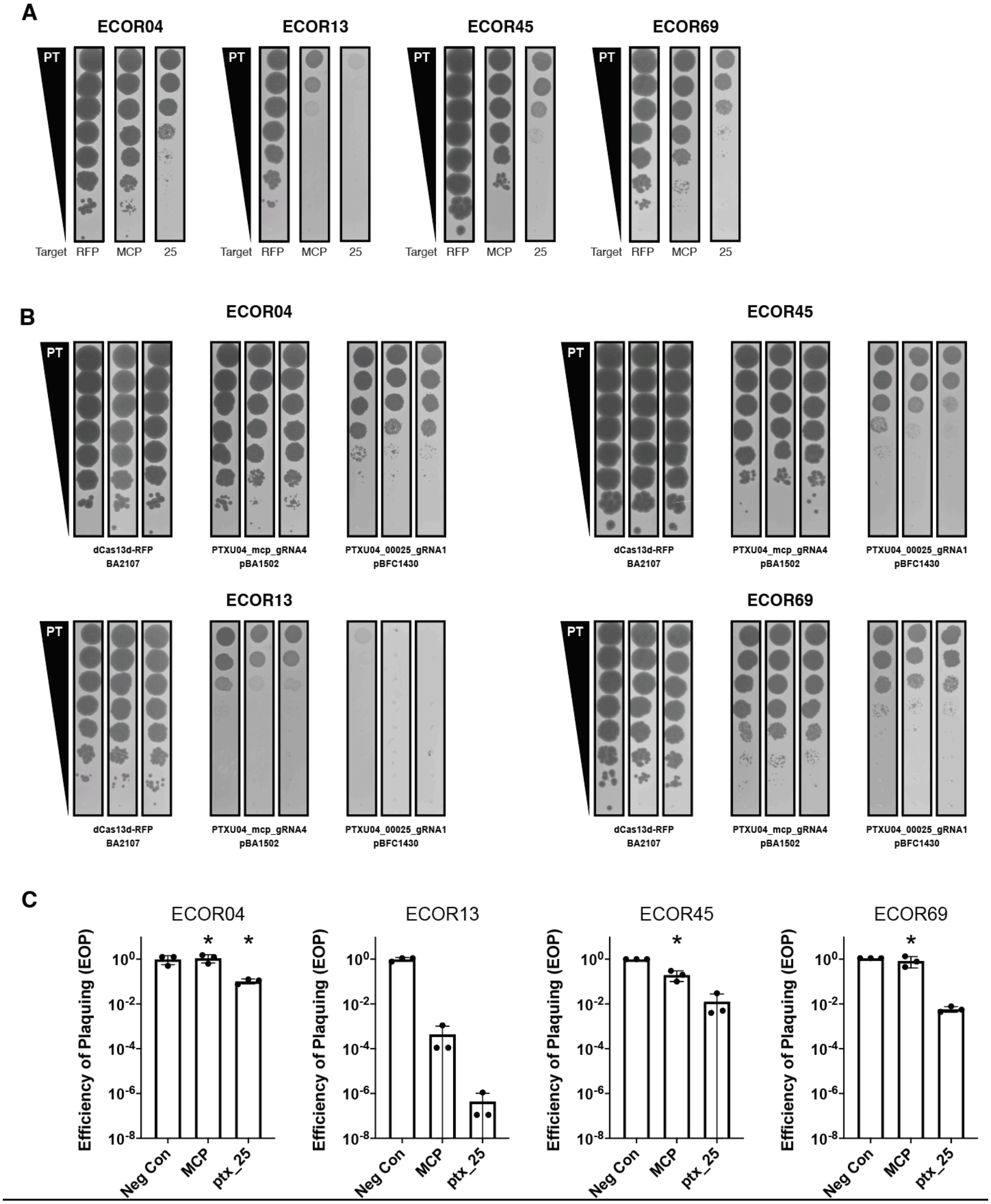

Supplementary Fig. 14 | CRISPRi-ART Inhibits Phage PTXU04 infection in Diverse *E. coli* ECOR strains. a, Representative plaque assays demonstrating the effectiveness of CRISPRi-ART in four genetically diverse *E. coli* ECOR strains,

ECOR04, ECOR13, ECOR45, and ECOR69, when targeting phage PTXU04 genes encoding mcp and PTXU04_00025. Cells expressing CRISPRi-ART constructs targeting mcp or PTXU04_00025 showed a significant reduction in EOP or plaque size when compared to cells expressing a non-targeting control crRNA. **b,** Replicate plaque assays corresponding to a. A guide targeting RFP is provided as a negative control. dRfxCas13d experiments were expressed using +100nM aTc. **c,** EOP measurements obtained from CRISPRi-ART validation plaque assays for three different phage sets scaled to their respective non-targeting control. Guides that led to a substantial reduction in plaque size but not EOP compared to the non-targeting crRNA (Control, Neg Con) are indicated with an asterisk. For **a-c**, data shown are for 3 biological replicates. Error bars are presented as mean +/- SD.

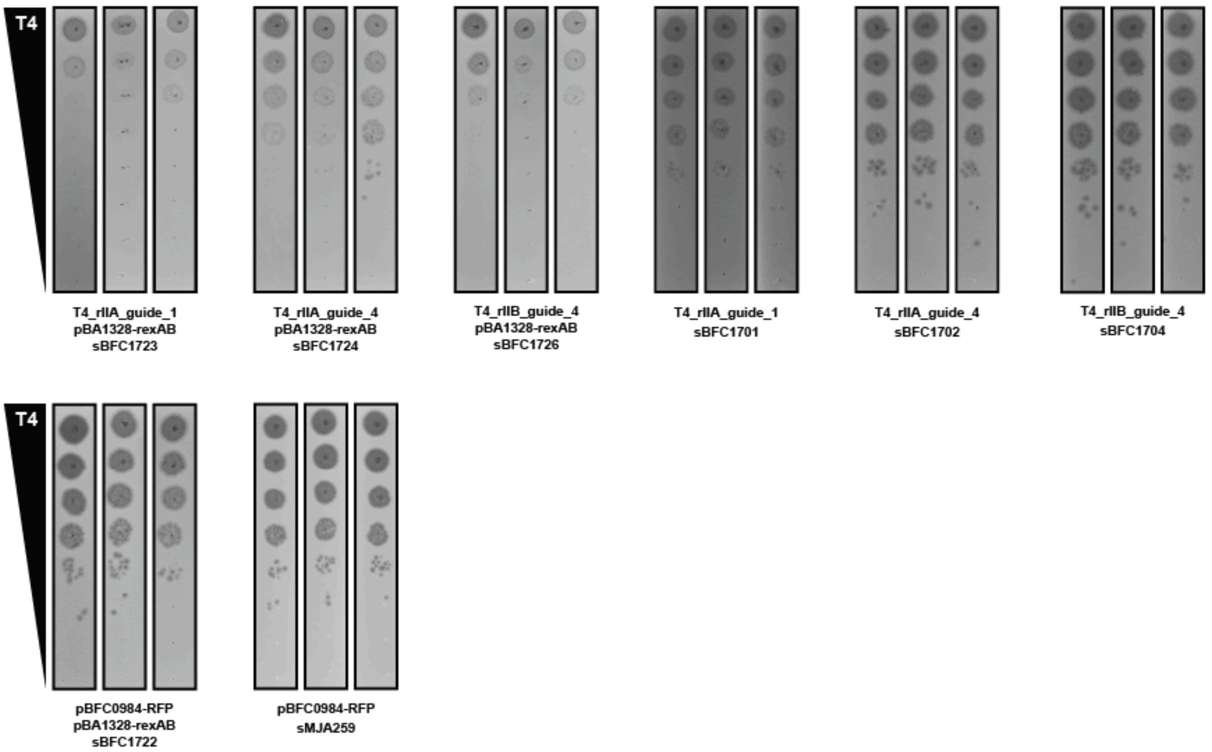

Supplementary Fig. 15 | T4 CRISPRi-ART RIIA/B Plaque Assays. Plaque assays for CRISPRi-ART-mediated phage defense when targeting phage T4 RIIA and RIIB RBSs with dRfxCas13d, data reported in Fig. 4d. A guide targeting RFP is provided as a negative control. dRfxCas13d experiments were expressed using +100nM aTc. Data shown are for 3 biological replicates. The first and fourth guide were selected for each of the two RII genes. Lambda RexA/B genes were expressed using +200nM Crystal Violet.

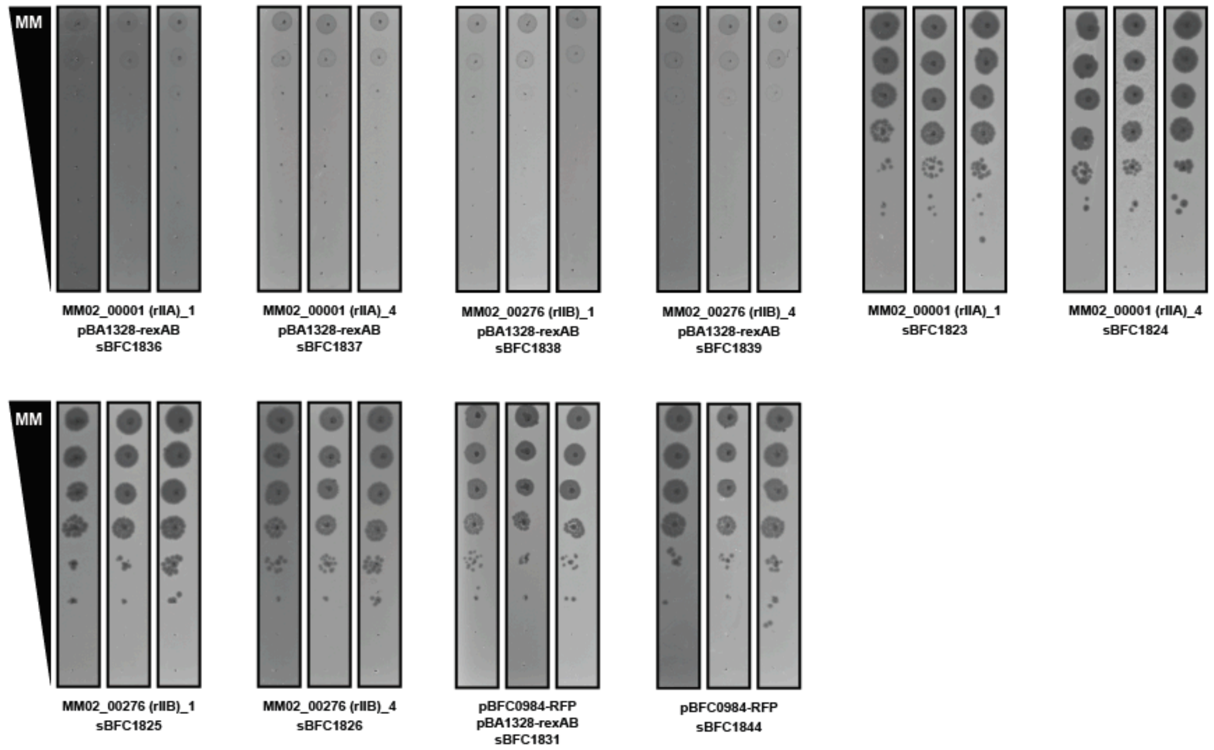

Supplementary Fig. 16 | MM02 CRISPRi-ART RIIA/B Plaque Assays. Plaque assays for CRISPRi-ART-mediated phage defense when targeting phage MM02 RIIA and RIIB RBSs with dRfxCas13d, data reported in Fig. 4d. A guide targeting RFP is provided as a negative control. dRfxCas13d experiments were expressed using +100nM aTc. Data shown are for 3 biological replicates. The first and fourth guide were selected for each of the two RII genes. Lambda RexA/B genes were expressed using +200nM Crystal Violet.

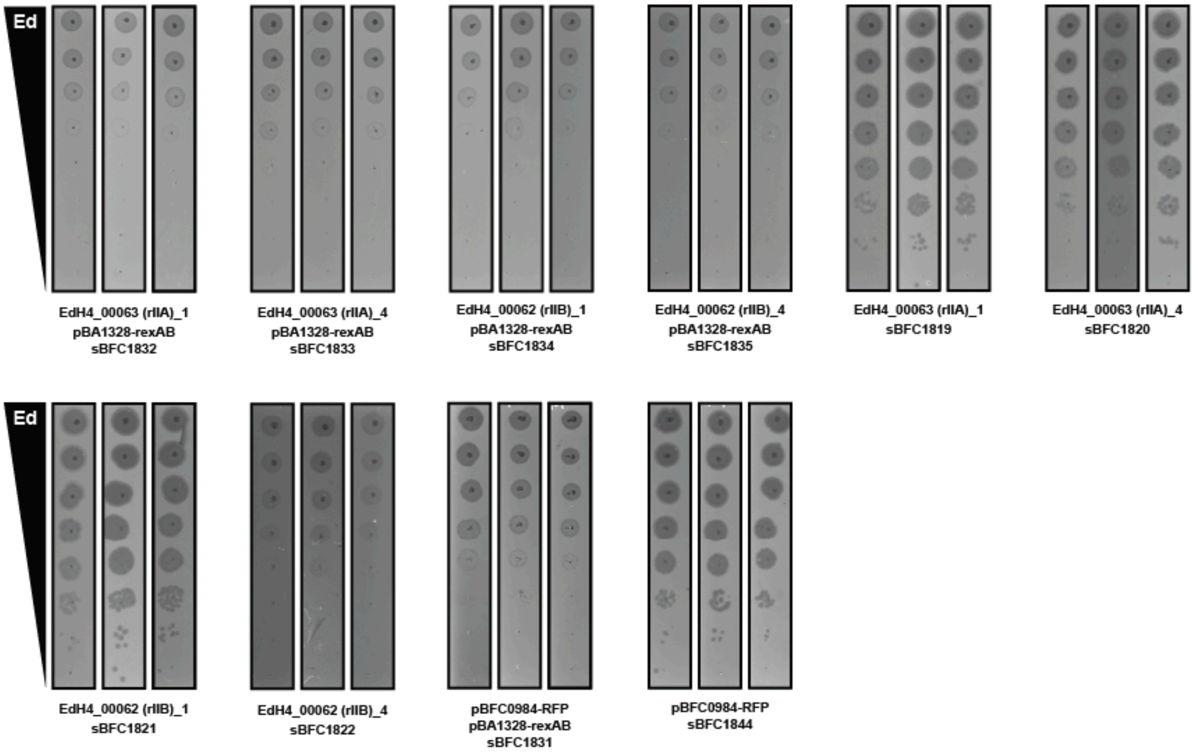

Supplementary Fig. 17 | EdH4 CRISPRi-ART RIIA/B Plaque Assays. Plaque assays for CRISPRi-ART-mediated phage defense when targeting phage EdH4 RIIA and RIIB RBSs with dRfxCas13d, data reported in Fig. 4d. A guide targeting RFP is provided as a negative control. dRfxCas13d experiments were expressed using +100nM aTc. Data shown are for 3 biological replicates. The first and fourth guide were selected for each of the two RII genes. Lambda RexA/B genes were expressed using +200nM Crystal Violet.

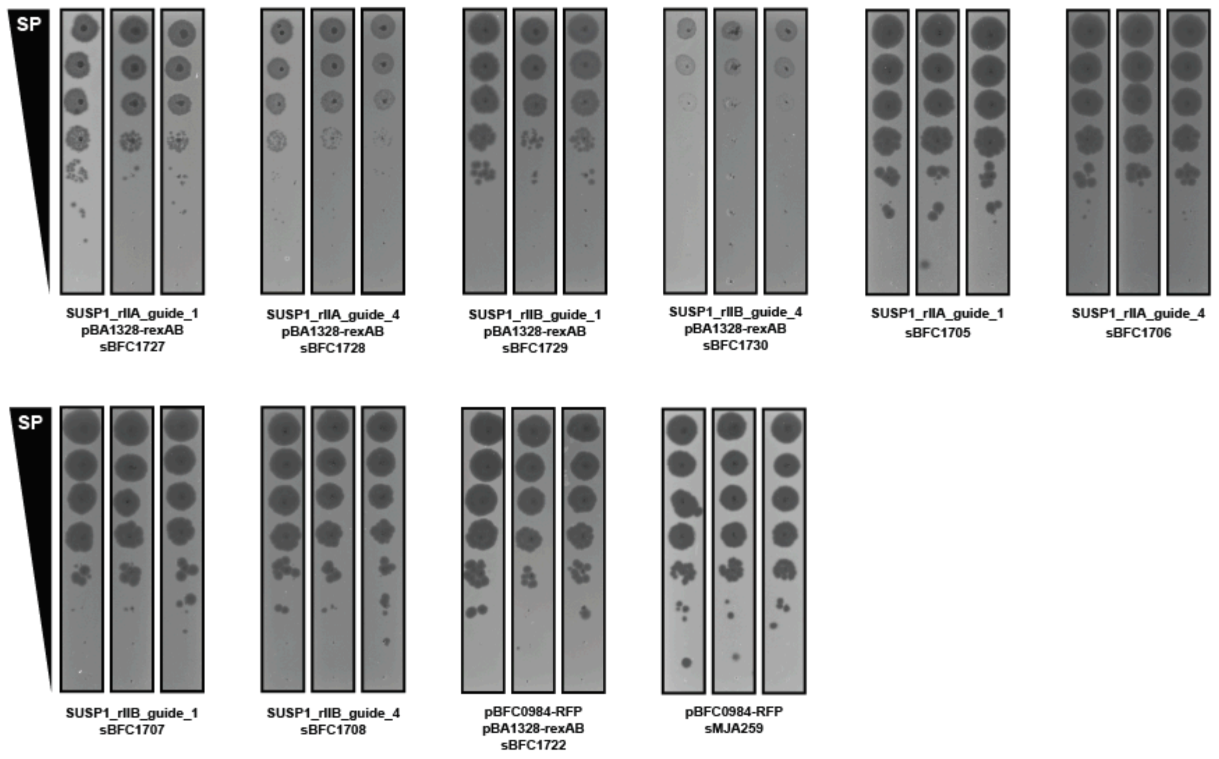

Supplementary Fig. 18 | SUSP1 CRISPRi-ART RIIA/B Plaque Assays. Plaque assays for CRISPRi-ART-mediated phage defense when targeting phage SUSP1 RIIA and RIIB RBSs with dRfxCas13d, data reported in Fig. 4d. A guide targeting RFP is provided as a negative control. dRfxCas13d experiments were expressed using +100nM aTc. Data shown are for 3 biological replicates. The first and fourth guide were selected for each of the two RII genes. Lambda RexA/B genes were expressed using +200nM Crystal Violet.

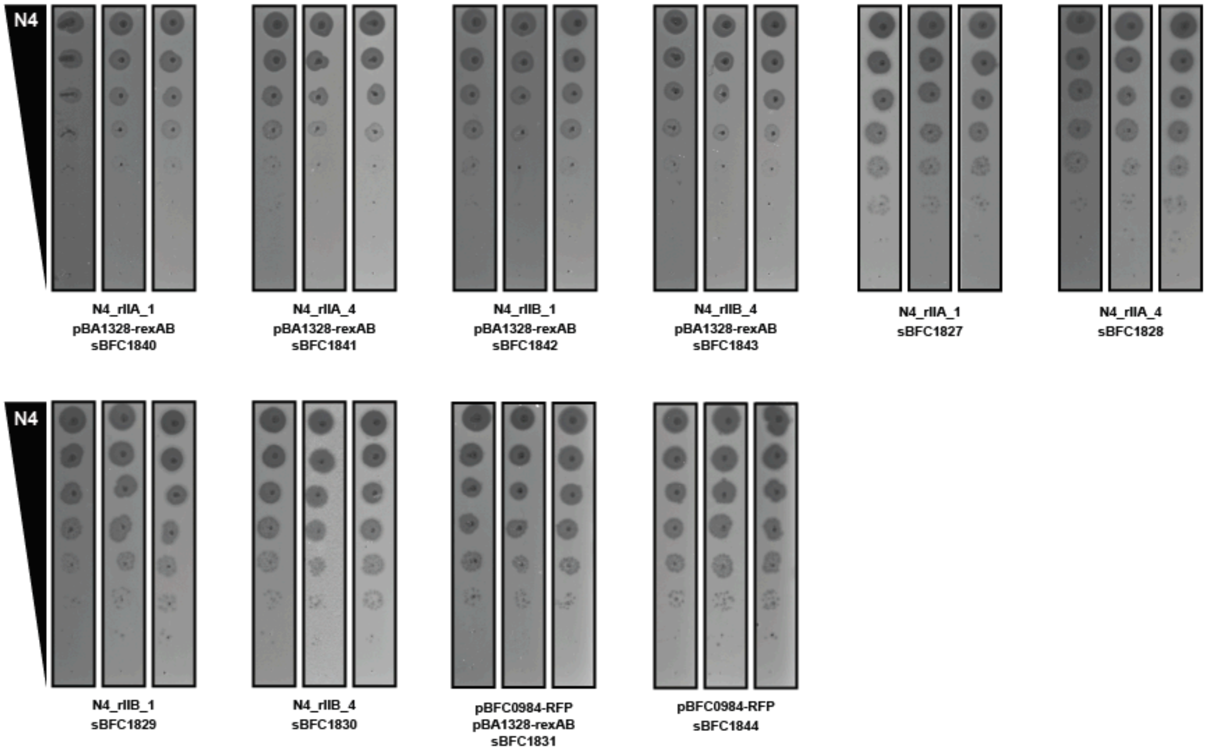

Supplementary Fig. 19 | N4 CRISPRi-ART RIIA/B Plaque Assays. Plaque assays for CRISPRi-ART-mediated phage defense when targeting phage N4 RIIA and RIIB RBSs with dRfxCas13d, data reported in Fig. 4d. A guide targeting RFP is provided as a negative control. dRfxCas13d experiments were expressed using +100nM aTc. Data shown are for 3 biological replicates. The first and fourth guide were selected for each of the two RII genes. Lambda RexA/B genes were expressed using +200nM Crystal Violet.

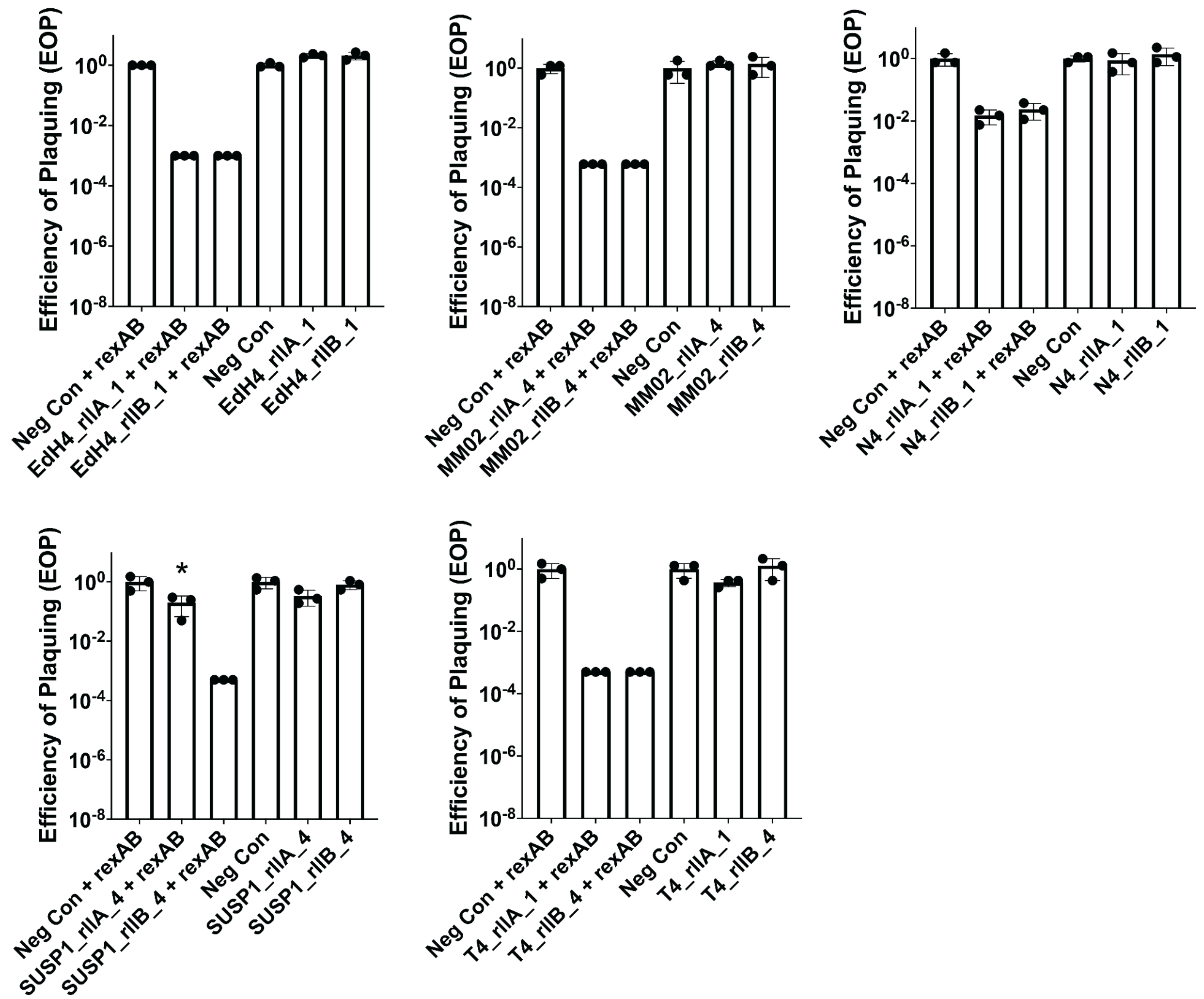

Supplementary Fig. 20 | CRISPRi-ART RIIA/B Plaque Assay EOPs. EOP measurements of rII triplicate plaque assays. Guides that led to a significant reduction in plaque size compared to the non-targeting crRNA control (Neg Con) as measured by a two-way ANOVA are indicated with an asterisk (adjusted p-value < 0.0001), reported only when there is not a significant reduction in EOP. Error bars are presented as mean +/- SD.

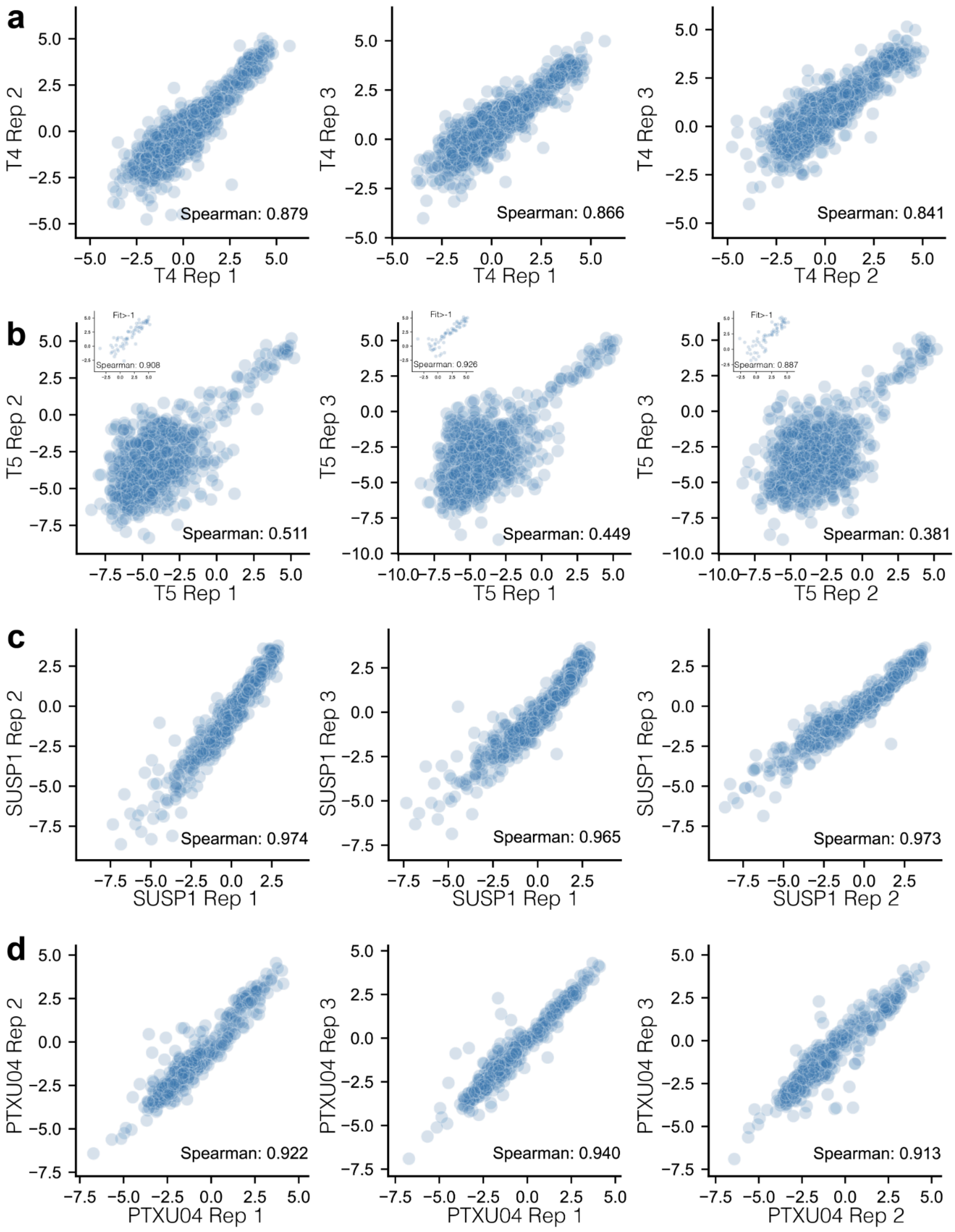

Supplementary Fig. 21 | Transcriptome-Wide CRISPRi-ART Replicate Correlation Plots. Pairwise comparison of crRNA fitness between replicates of pooled phage CRISPRi-ART assays indicates high reproducibility. **a,** T4. **b,** T5 with inset highlighting

high correlation of crRNA fitness between replicates for high fitness crRNAs (Fit>-1). **c,** SUSP1. **d,** PTXU04. For all plots, crRNA fitness Spearman correlation coefficient is provided between replicate experiments.

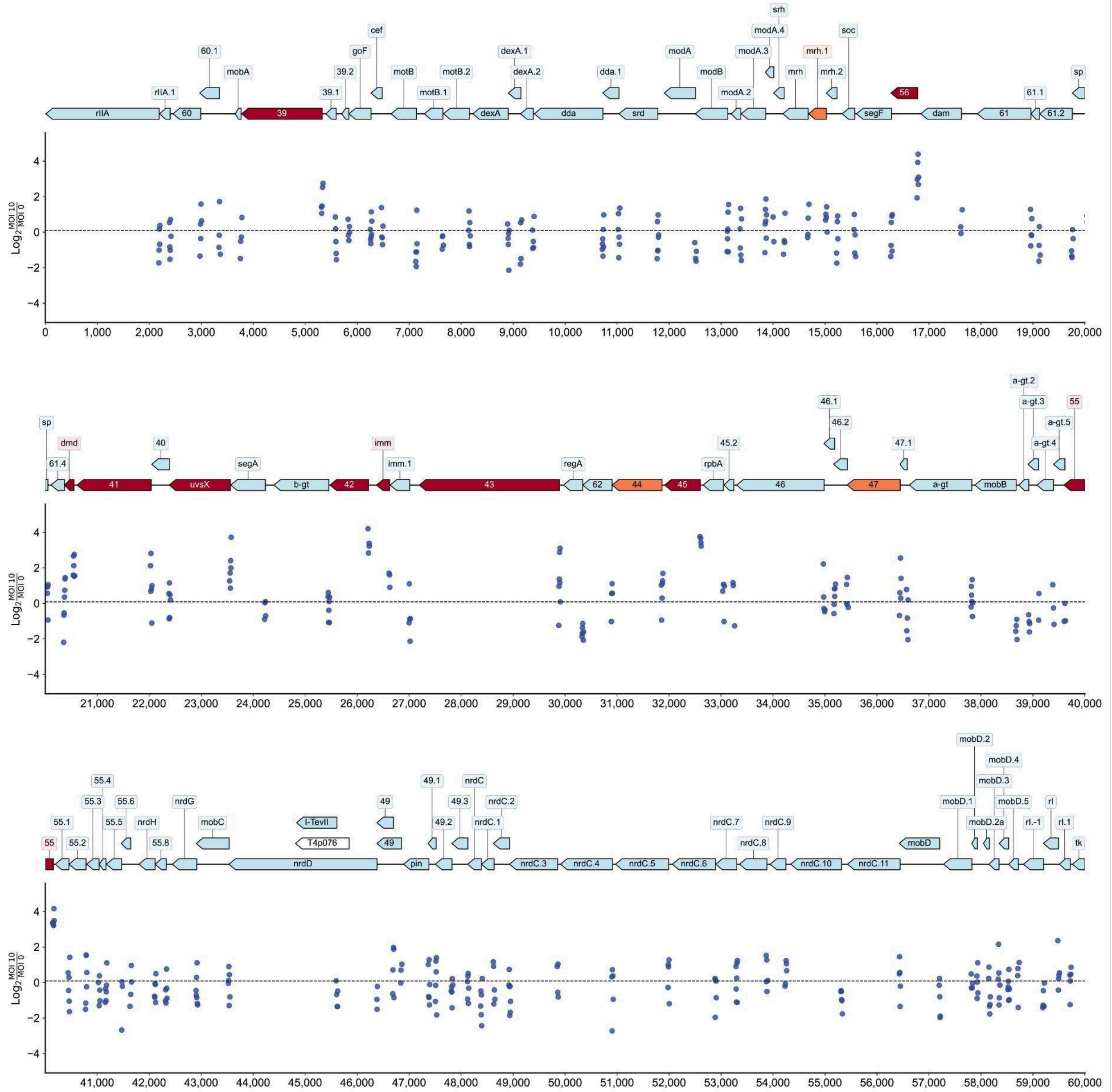

Supplementary Fig. 22 | Transcriptome-wide CRISPRi-ART Fitness for Phage T4 (continued below)

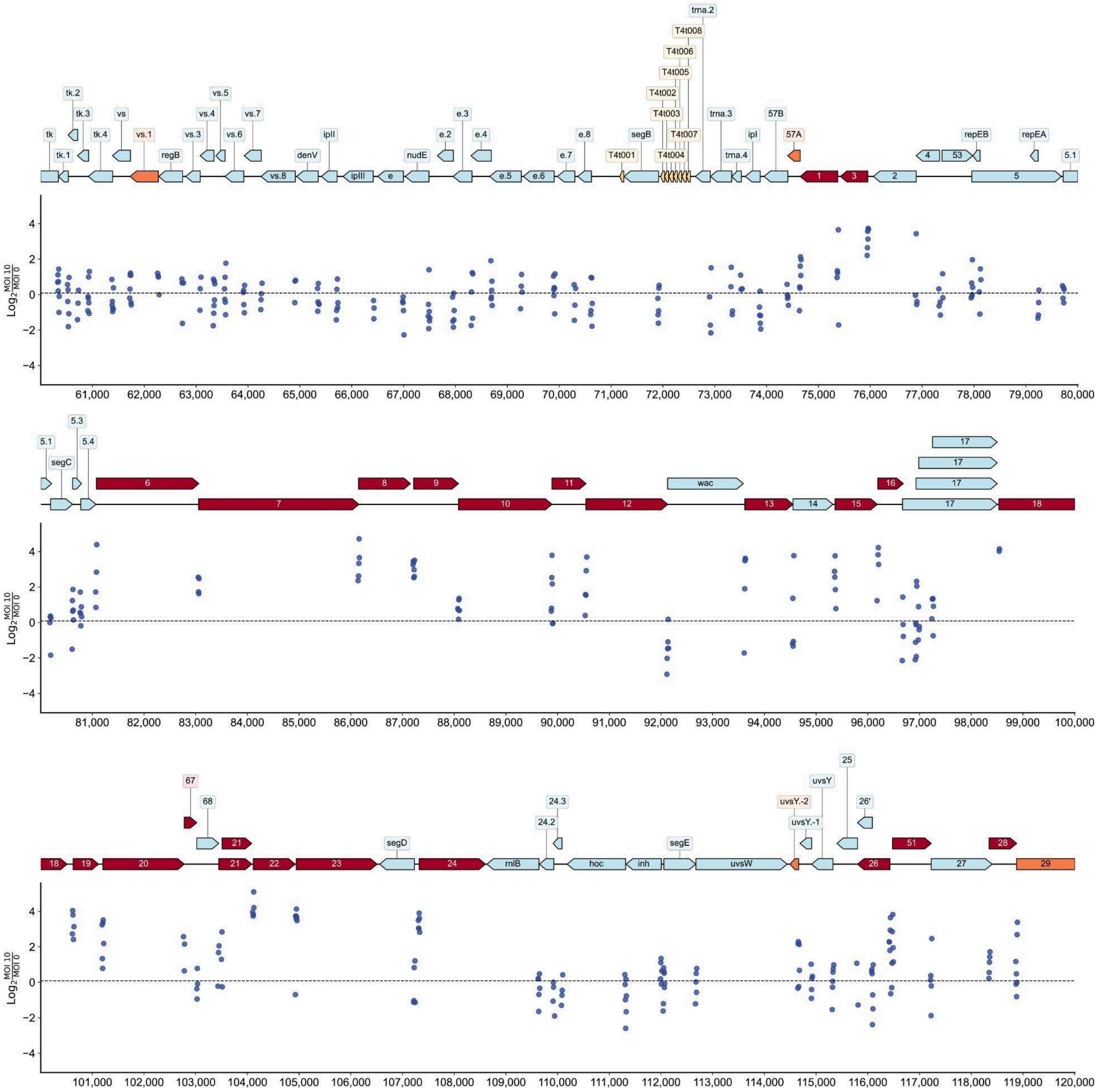

Supplementary Fig. 22 | Transcriptome-wide CRISPRi-ART Fitness for Phage T4 (continued below)

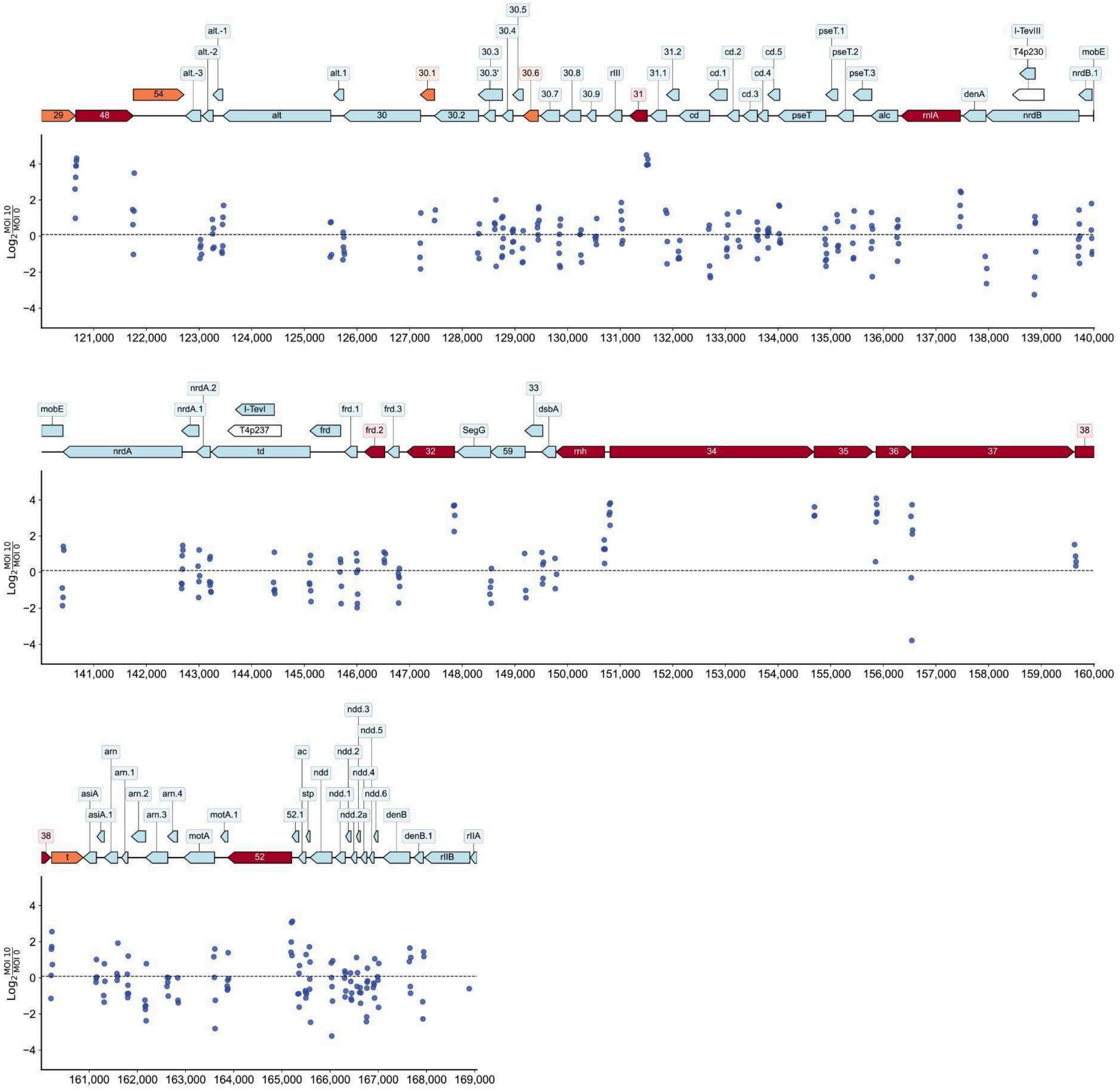

Supplementary Fig. 22 | Transcriptome-wide CRISPRi-ART Fitness for Phage T4. (Top track) Gene overview of phage T4 (NC_000866.4). Coding genes are shown in light blue and highlighted in red if they met significance thresholds (Methods). tRNAs are shown in orange. Other noncoding genetic elements are shown in white. (Middle track) dCas13d gene knockdown fitness when targeting T4 genes during infection at 10 MOI. Median guide fitness is shown with a dashed line. (Bottom track) dCas13d gene knockdown fitness when targeting T4 genes during infection at 100 MOI. Median guide fitness is shown with a dashed line.

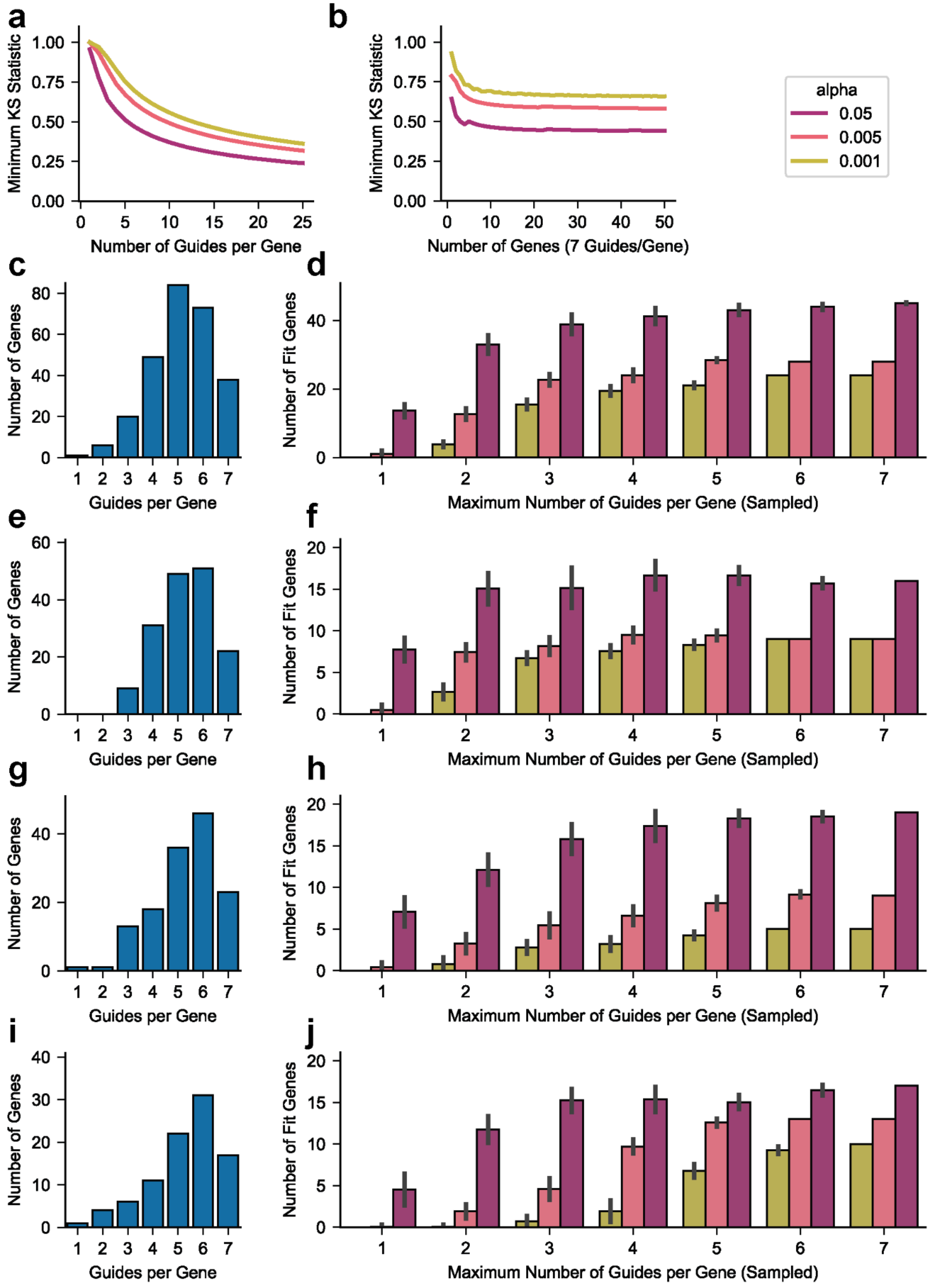

a
Minimum KS Statistic
1.00
0.75
0.50
0.25
0.00
0
5
10
15
20
25
Number of Guides per Gene
b
Minimum KS Statistic
0
10
20
30
40
50
Number of Genes (7 Guides/Gene)
alpha
0.05
0.005
0.001
c
Number of Genes
Guides per Gene
d
Number of Fit Genes
Maximum Number of Guides per Gene (Sampled)
e
Number of Genes
Guides per Gene
f
Number of Fit Genes
Maximum Number of Guides per Gene (Sampled)
g
Number of Genes
Guides per Gene
h
Number of Fit Genes
Maximum Number of Guides per Gene (Sampled)
i
Number of Genes
Guides per Gene
j
Number of Fit Genes
Maximum Number of Guides per Gene (Sampled)

Supplementary Fig. 23 | Theoretical and Post-Hoc Analysis of CRISPRi-ART Library Design. a, Minimum power required for two-sample, one-sided K-S comparison as varied by number of guides targeting per gene for a phage genome size of 273 genes at multiple levels of confidence. **b,** Minimum power required for two-sample, one-sided K-S comparison as varied by number of genes in a genome using 7 guides per gene at multiple levels of confidence. **c, e, g, i,** Empirical distribution of the number of guides per gene passing QC in the MOI 0 condition that were used in analysis for this manuscript. **d, f, h, j,** Simulation of Fit genes called in this study by post-hoc varying the number of guides used per gene. Simulation was performed by recalculating gene fitness after randomly sampling k guides per gene for all reported library experiments (or the maximum number if less than k). Sampling across the entire dataset was performed 50 times and gene fitness calculated from guide fitness as described in Methods. The total number of "Fit" genes with p-like statistics of 0.05, 0.005 and 0.001 were calculated and presented as mean±standard deviation. Panels c and d refer to T4 library experiments. Panels e and f refer to T5 library experiments. Panels g and h refer to SUSP1 library experiments. Panels **i** and **j** refer to PTXU04 library experiments. For panels **a,b,d,f,h,j** results are shown for multiple levels of confidence, alpha (p-like statistic), of 0.05, 0.005, 0.001.

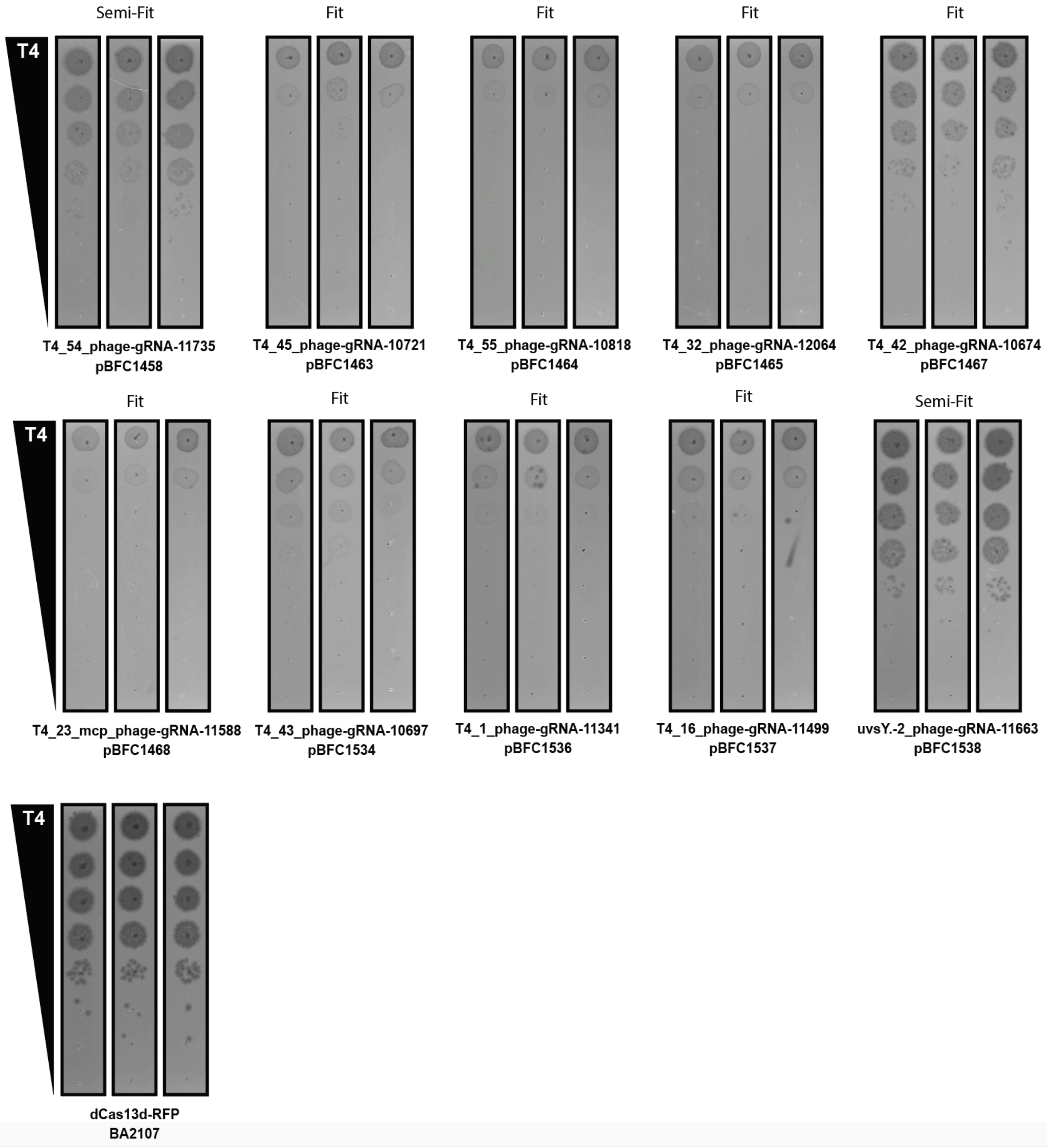

Supplementary Fig. 24 | T4 CRISPRi-ART Single crRNA Validation Plaque Assays. Plaque assays to validate pooled CRISPRi-ART results using single crRNA to target phage T4 RBS with dRfxCas13d. Guides were selected for their performance in the high-throughput screen. 10 genes of both known and unknown function of semi-fit and fit classification were selected for plaque assay validation. A guide targeting RFP is provided as a negative control. dRfxCas13d was expressed using +20nM aTc. Data shown are for 3 biological replicates.

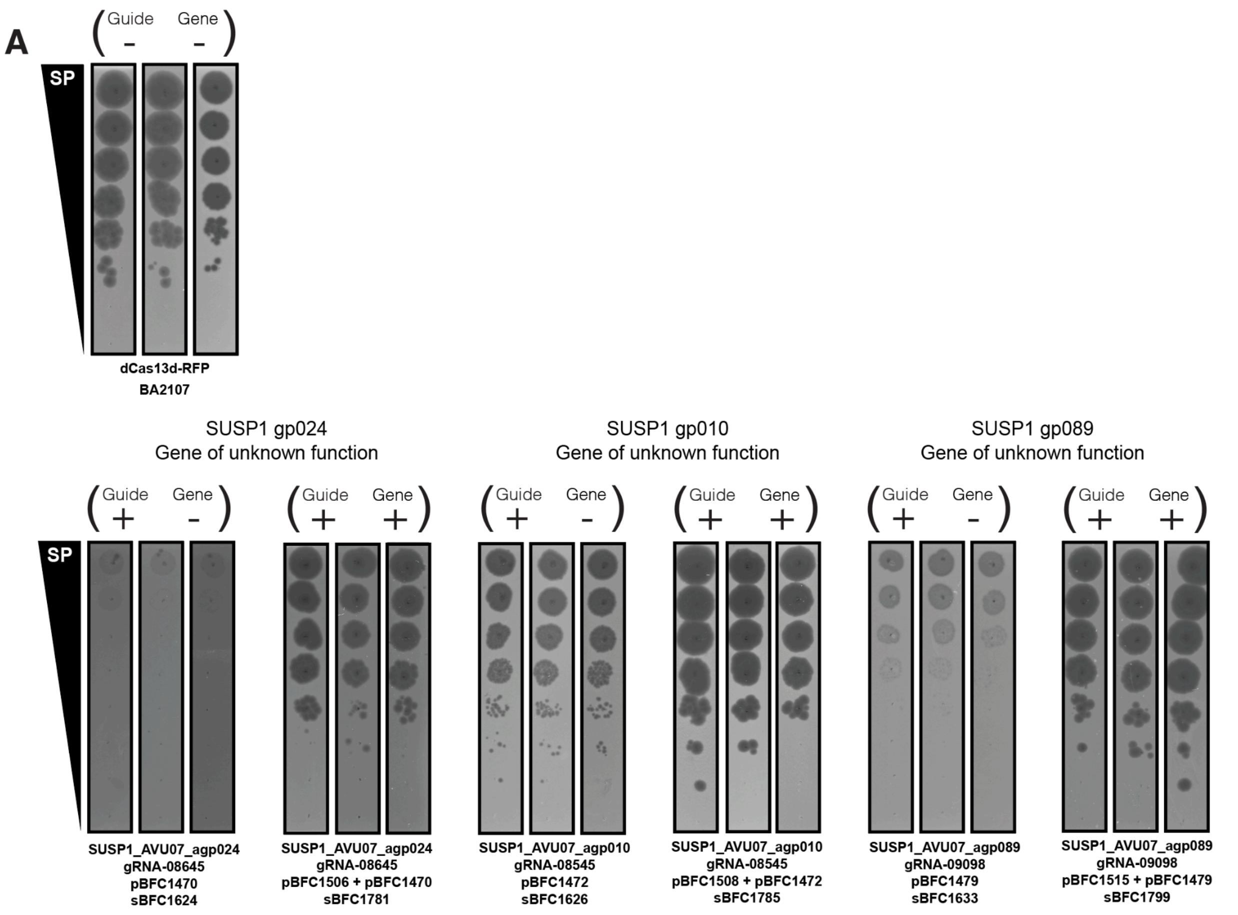

Supplementary Fig. 25 | T4 and SUSP1 CRISPRi-ART Complementation Replicates (Continued Below).

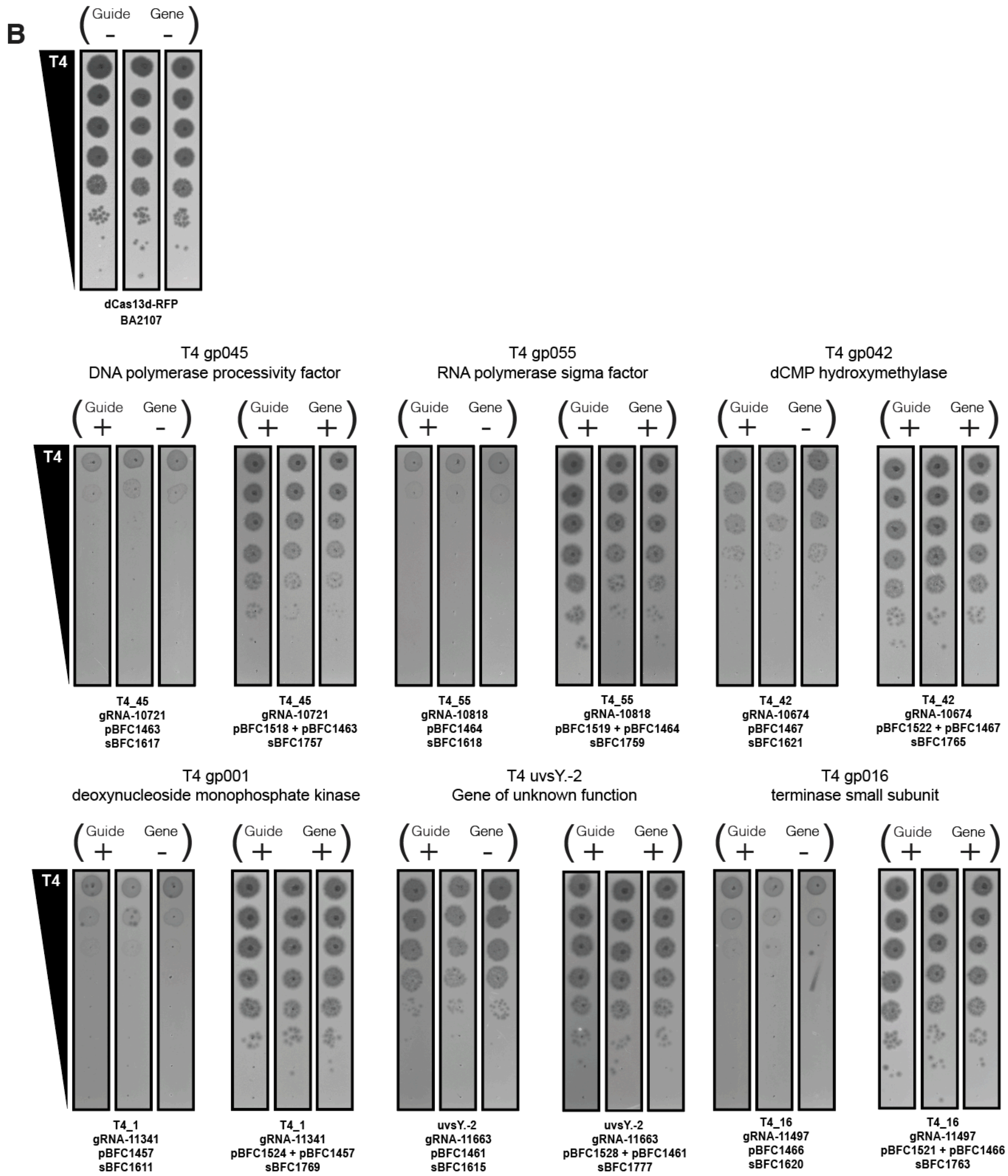

Supplementary Fig. 25 | T4 and SUSP1 CRISPRi-ART Complementation Replicates. Plaque assays for CRISPRi-ART-mediated phage defense targeting T4 (**a**) and SUSP1 (**b**) genes at their ribosome-binding site (RBS) using dRfxCas13d. A guide targeting rfp served as a negative control. Experiments were conducted at aTc concentrations consistent with previous assays, and complemented genes were expressed using 200 nM crystal violet. Data represent three biological replicates. Genes

that exhibited toxicity upon induction were excluded from complementation plaque assays.

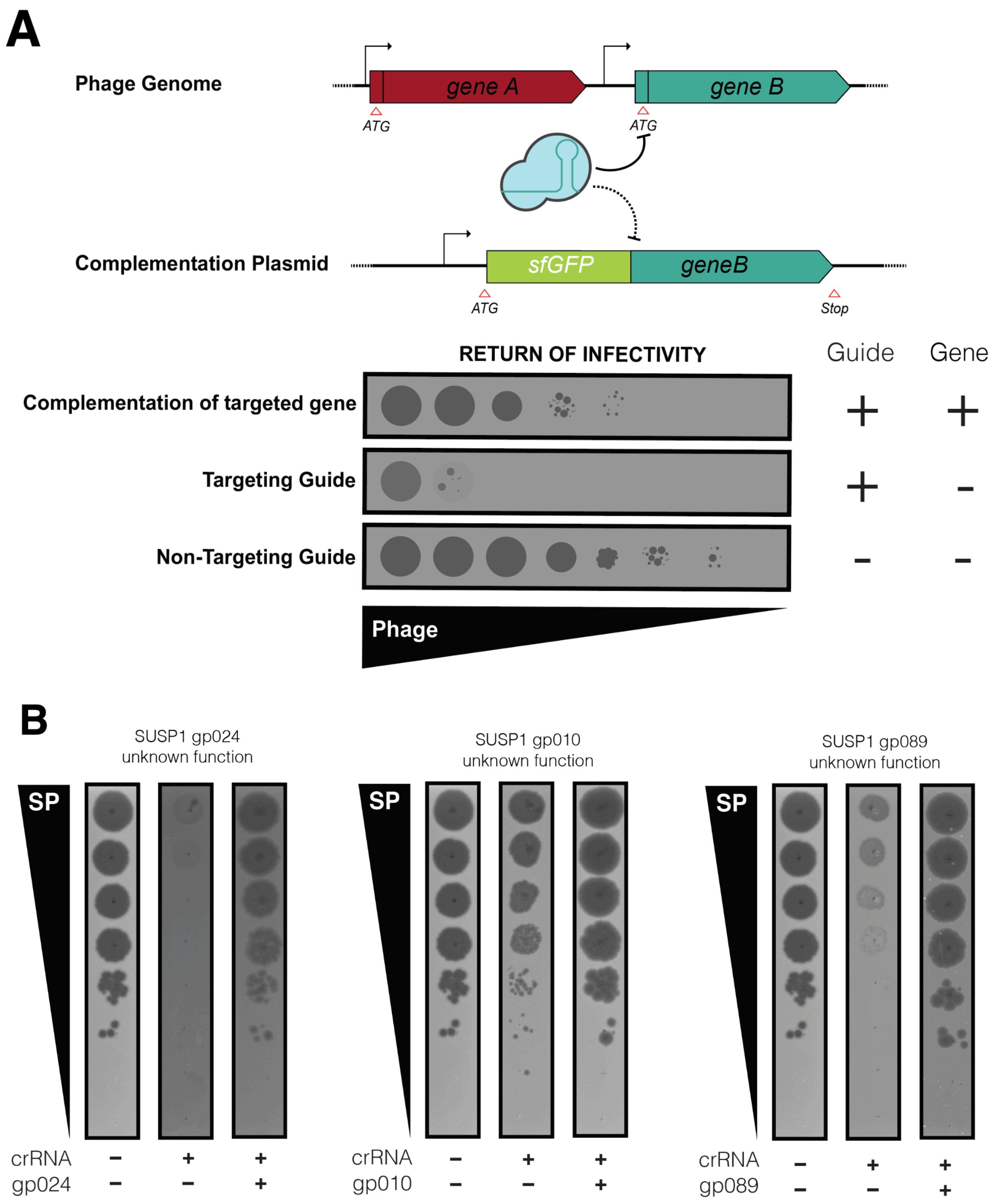

Supplementary Fig. 26 | T4 dCas13d CRISPRi plaque assays. (Continued Below)

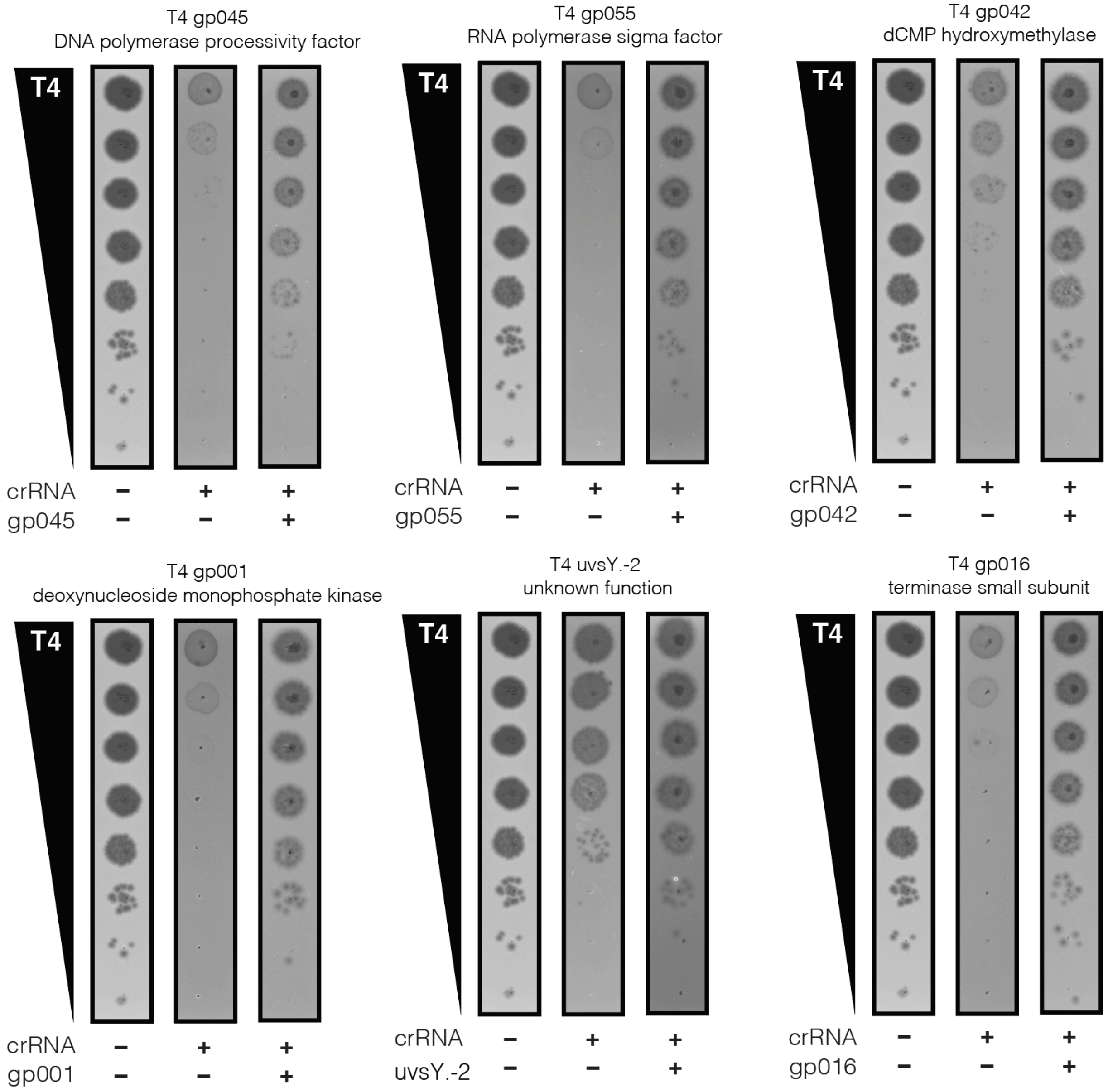

Supplementary Fig. 26 | T4 dCas13d CRISPRi plaque assays. a, Model of complementation. Genes of interest were cloned out of their native RBS context and cloned inline with sfGFP in a plasmid containing an alternate RBS. This ensures that the only available binding site is present in the phage genome. **b,** Phage gene expression in the presence of infection presented side by side negative control and guide plasmid.

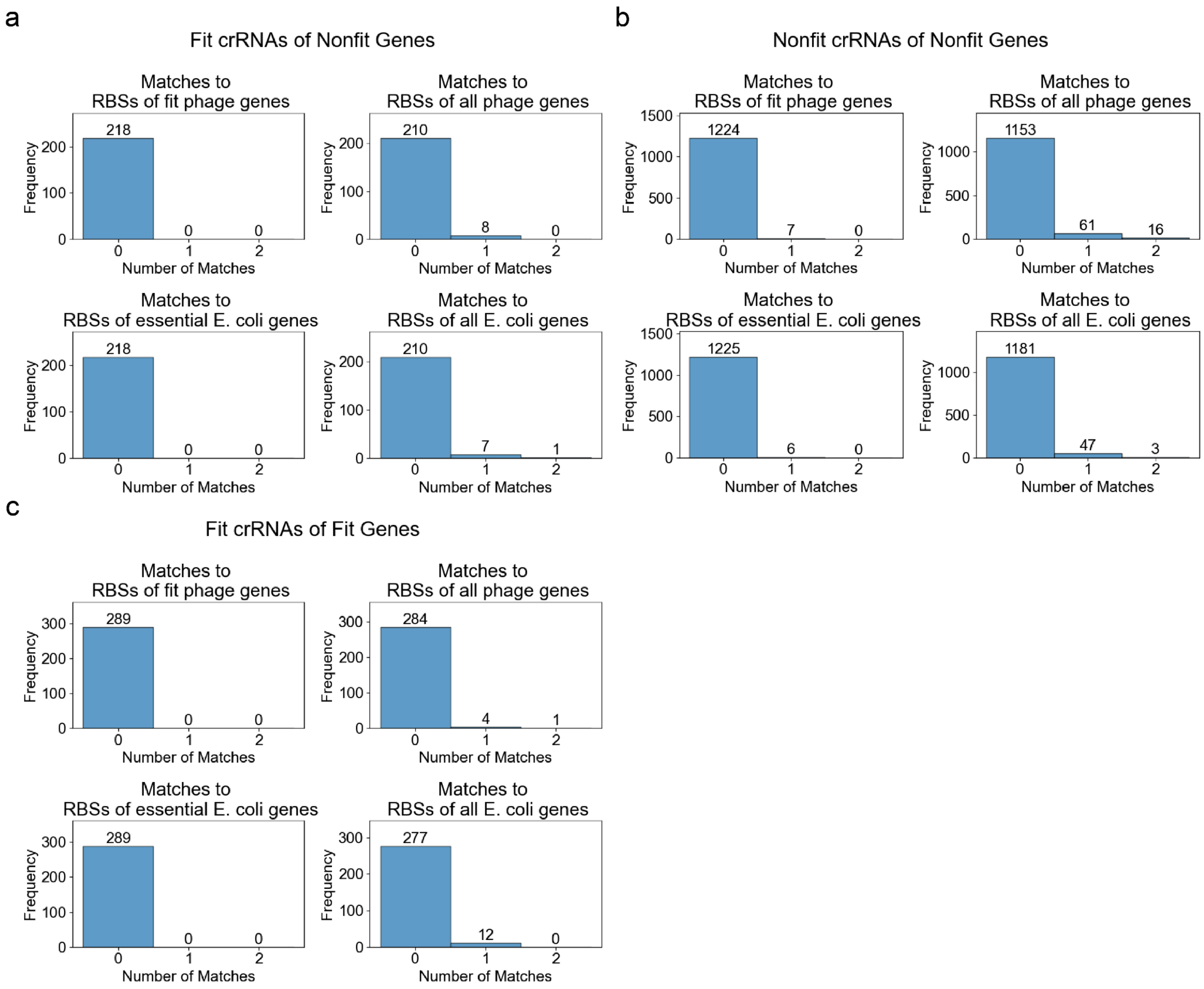

Supplementary Fig. 27 | Phage CRISPRi-ART Off-Target Analysis. For each crRNA in the phage T4 CRISPRi-ART library, an *in silico* analysis was performed to quantify potential off-target binding in the susceptible RBS region (Fig. 1d). The processed 21 nt spacer sequence of each crRNA was queried against the RBS regions of all or Fit T4 and essential *E. coli* genes. As delineated in Fig. 1d, the RBS region of each gene was defined as +/-35 bp flanking the start codon. Up to 3 mismatches were tolerated for calling matches to the 21 nt query. The frequency at which crRNAs matched off-target RBS regions is plotted as a histogram. Analysis is performed for **a,** crRNAs which had a significant high fitness score (Fit), despite targeting a T4 gene classified in the transcriptome-wide screen as Not Fit, **b,** crRNAs which did not have a significant high fitness score (Nonfit), despite targeting a T4 gene classified in the transcriptome-wide screen as Not Fit, and **c,** crRNAs which had a significant high fitness score (Fit), while targeting a T4 gene classified in the transcriptome-wide screen as Fit.

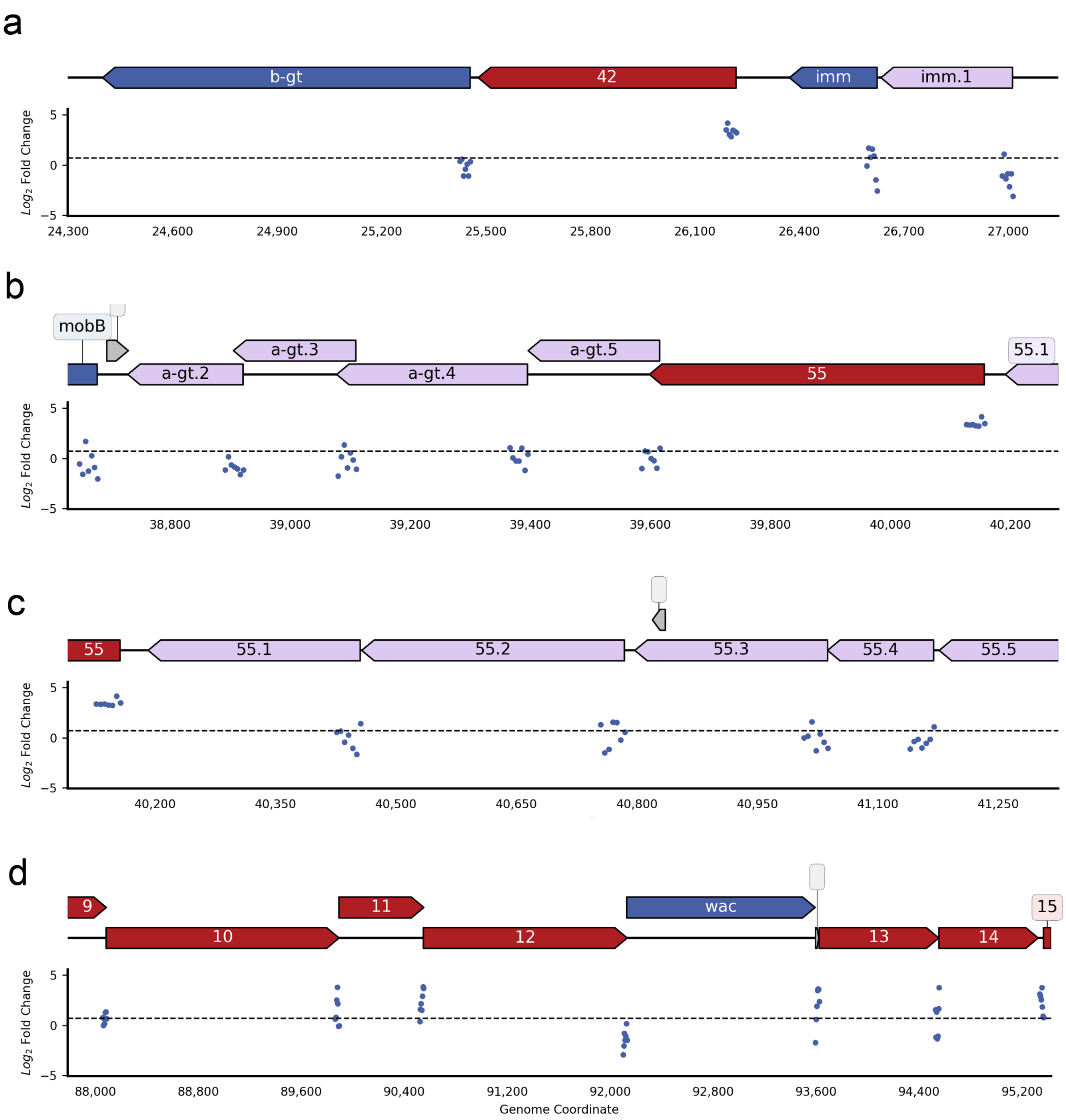

Supplementary Fig. 28 | Assessment of CRISPRi-ART Polarity in T4. Select operons (**a**–**d**) from the T4 genome containing genes of mixed essentiality are highlighted. For each operon, genome tracks are shown alongside the Log2 fold change values for each tested crRNA (MOI 10 vs. MOI 0). The dotted line indicates the threshold used to assign a positive fitness score. Known essential genes are colored red, known non-essential genes are blue, and genes of unknown essentiality are purple. Transcript boundaries and gene essentiality were determined using previously published data[25].

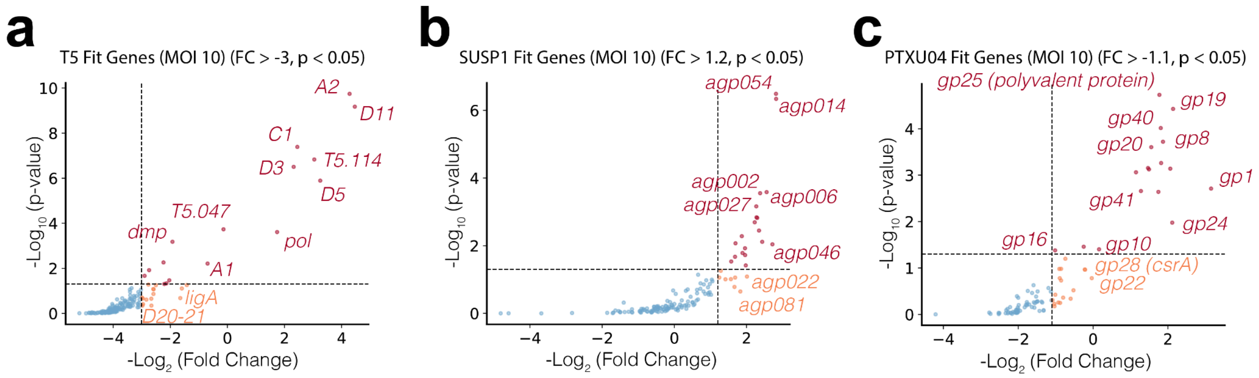

Supplementary Fig. 29 | T5, SUSP1, and PTXU04 Transcriptome-Wide Gene Fitness Summaries. Volcano plots with selection of Fit (red) and Semi-Fit (orange) genes indicated for phages **a,** T5 **b,** SUSP1 **c,** PTXU04. Thresholds for Fit, Semi-Fit, and Not Fit (blue) gene classifications are described in Methods. For **a-c**, gene fitness is shown as the mean of 3 biological replicates with log10-transformed unidirectional K-S p-value

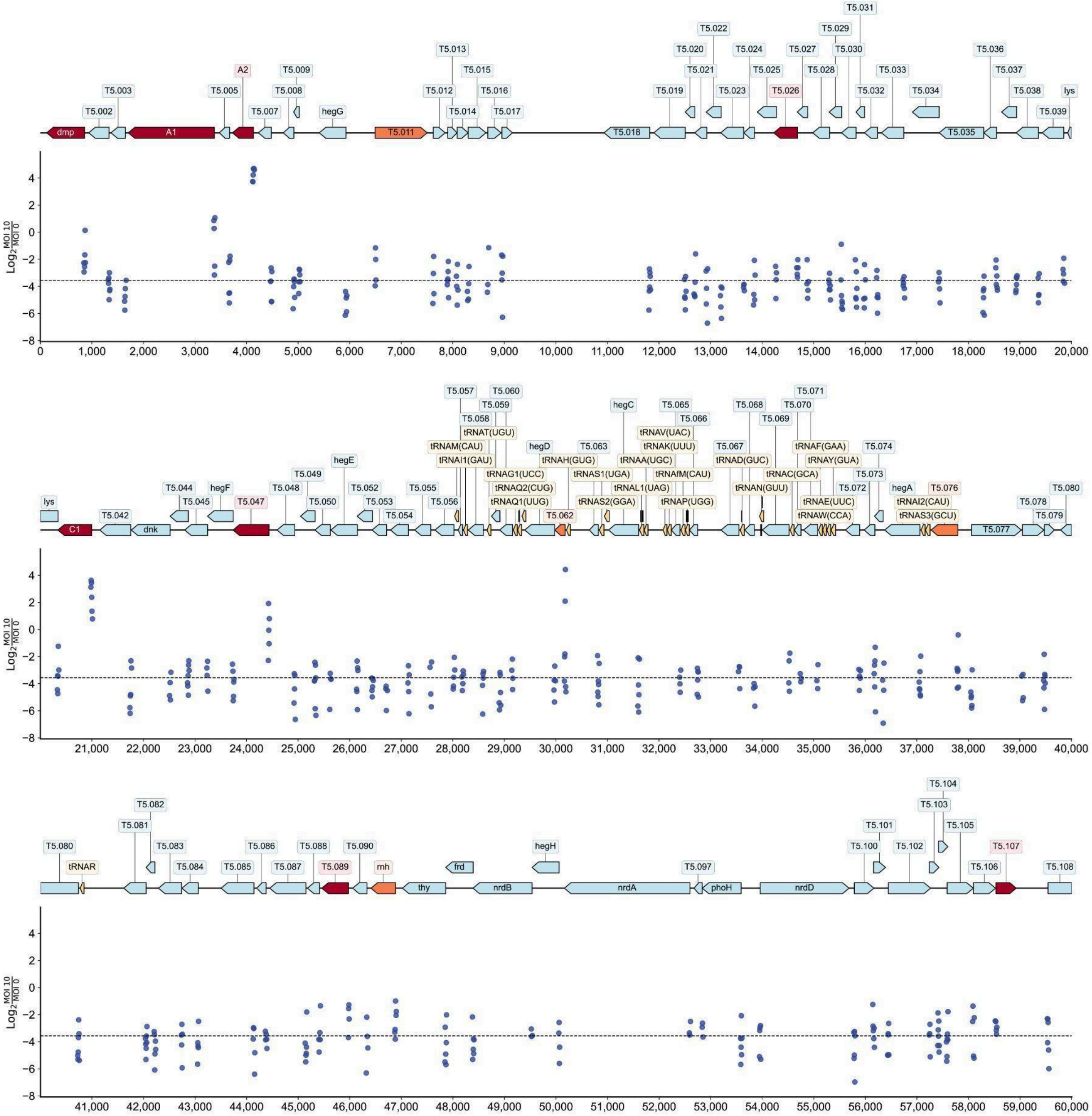

Supplementary Fig. 30 | Transcriptome-wide CRISPRi-ART Fitness for Phage T5 (continued below)

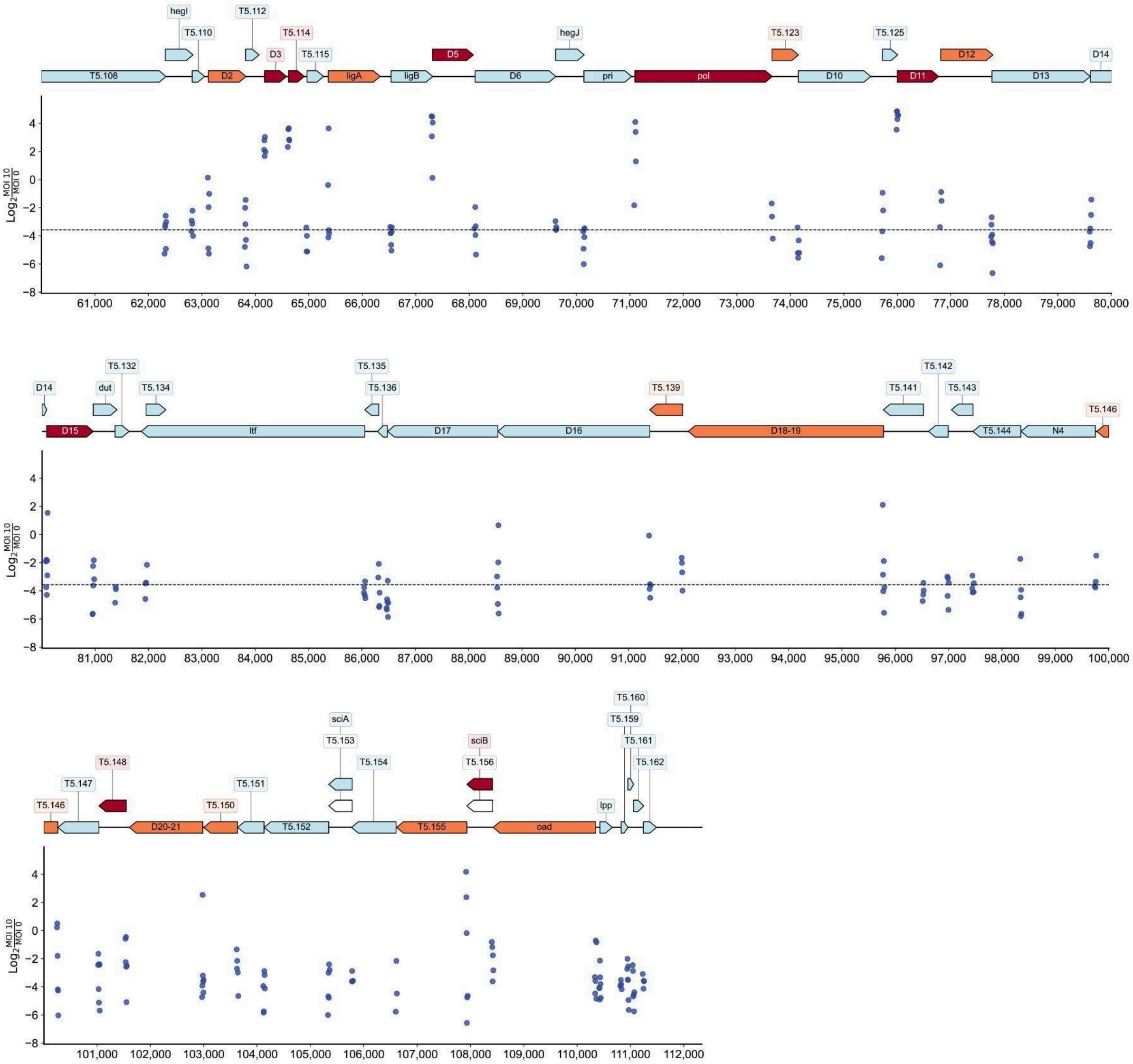

Supplementary Fig. 30 | Transcriptome-wide CRISPRi-ART Fitness for Phage T5. (Top track) Gene overview of phage T5 (NC_005859). Coding genes are shown in light blue and highlighted in red if they met significance thresholds (Methods). tRNAs are shown in orange. Other noncoding genetic elements are shown in white. (Middle track) dCas13d gene knockdown fitness when targeting T5 genes during infection at 10 MOI. Median guide fitness is shown with a dashed line. (Bottom track) dCas13d gene knockdown fitness when targeting T5 genes during infection at 100 MOI. Median guide fitness is shown with a dashed line.

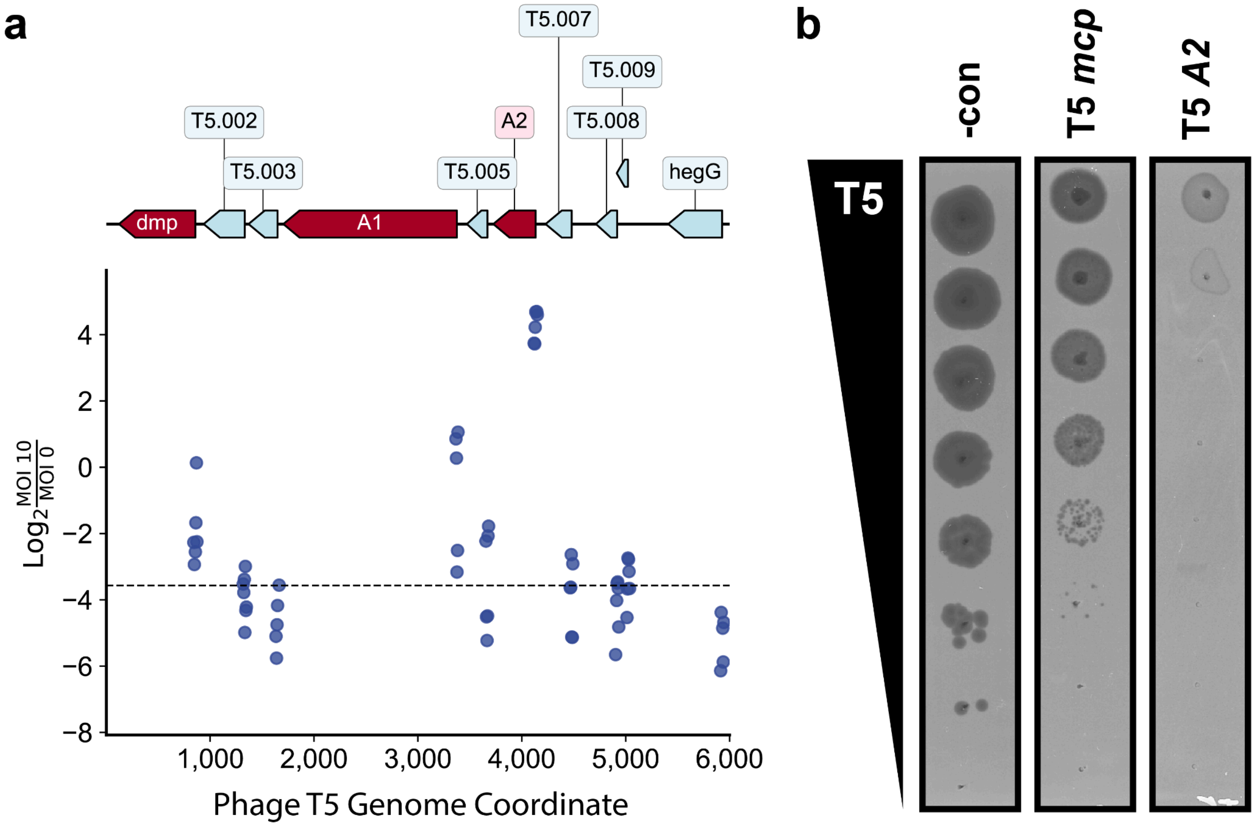

Supplementary Fig. 31 | Phage T5 Gene Fitness in Pre-Early Genes. a, Gene fitness measured for each crRNA in the pre-early region. **b,** Plaque assays targeting mcp and A2, highlighting the importance of the initial infection steps orchestrated by the genes expressed during FST.

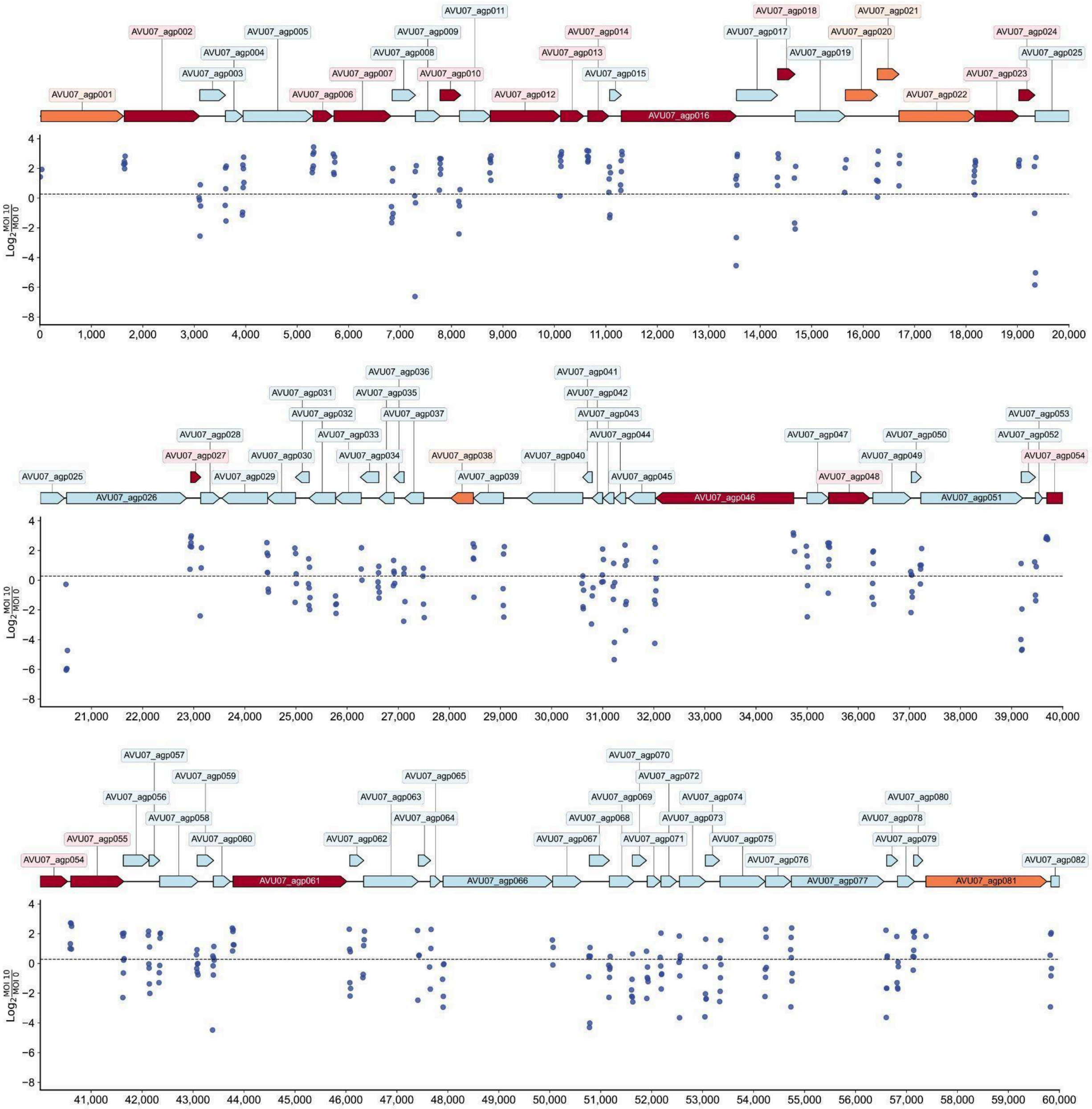

Supplementary Fig. 32 | Transcriptome-wide dCas13d-fitness for phage SUSP1 (continued below)

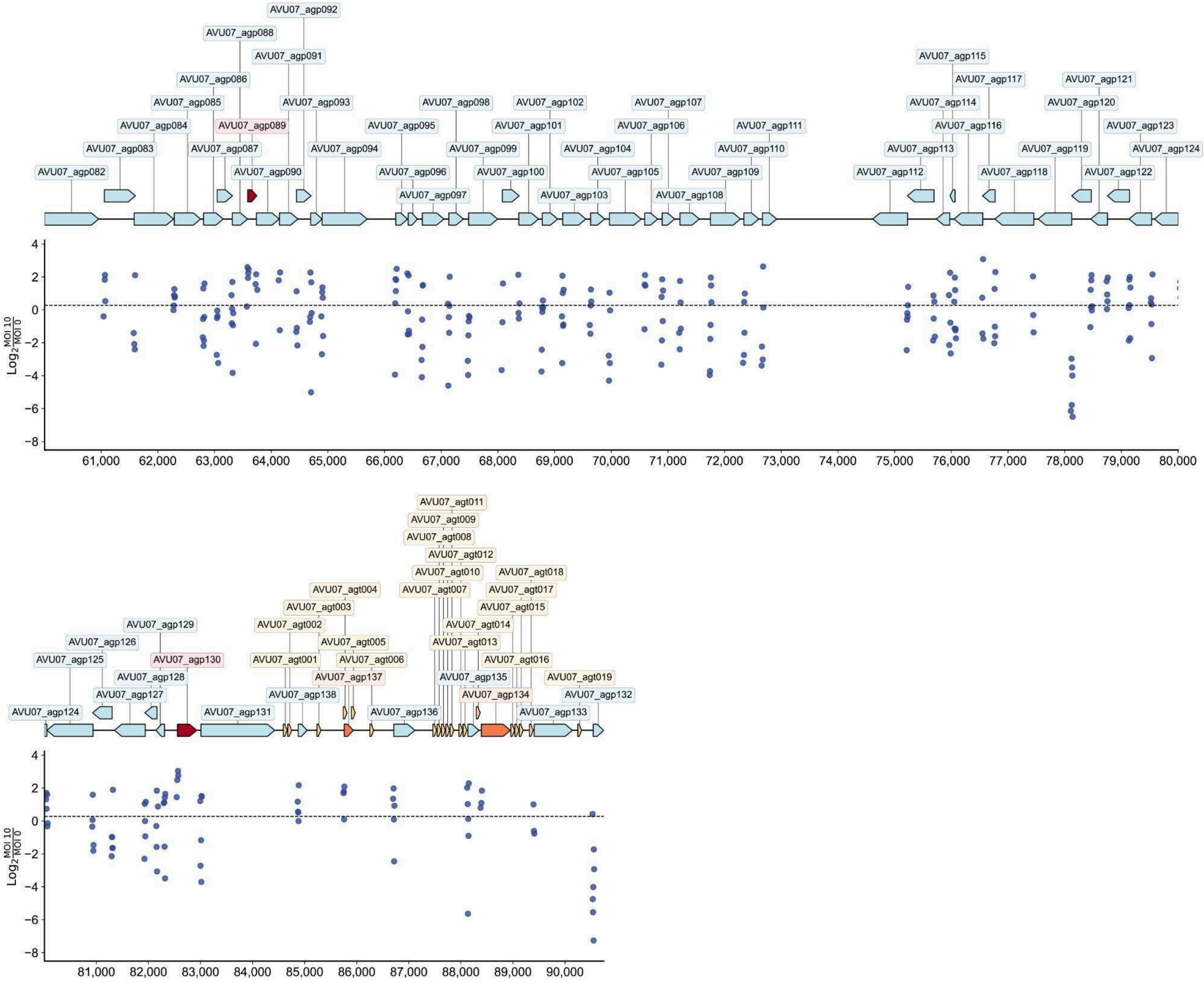

Supplementary Fig. 32 | Transcriptome-wide CRISPRi-ART Fitness for Phage SUSP1. (Top track) Gene overview of phage SUSP1 (NC_028808). Coding genes are shown in light blue and highlighted in red if they met significance thresholds (Methods). tRNAs are shown in orange. Other noncoding genetic elements are shown in white. (Middle track) dCas13d gene knockdown fitness when targeting SUSP1 genes during infection at 10 MOI. Median guide fitness is shown with a dashed line. (Bottom track) dCas13d gene knockdown fitness when targeting SUSP1 genes during infection at 100 MOI. Median guide fitness is shown with a dashed line.

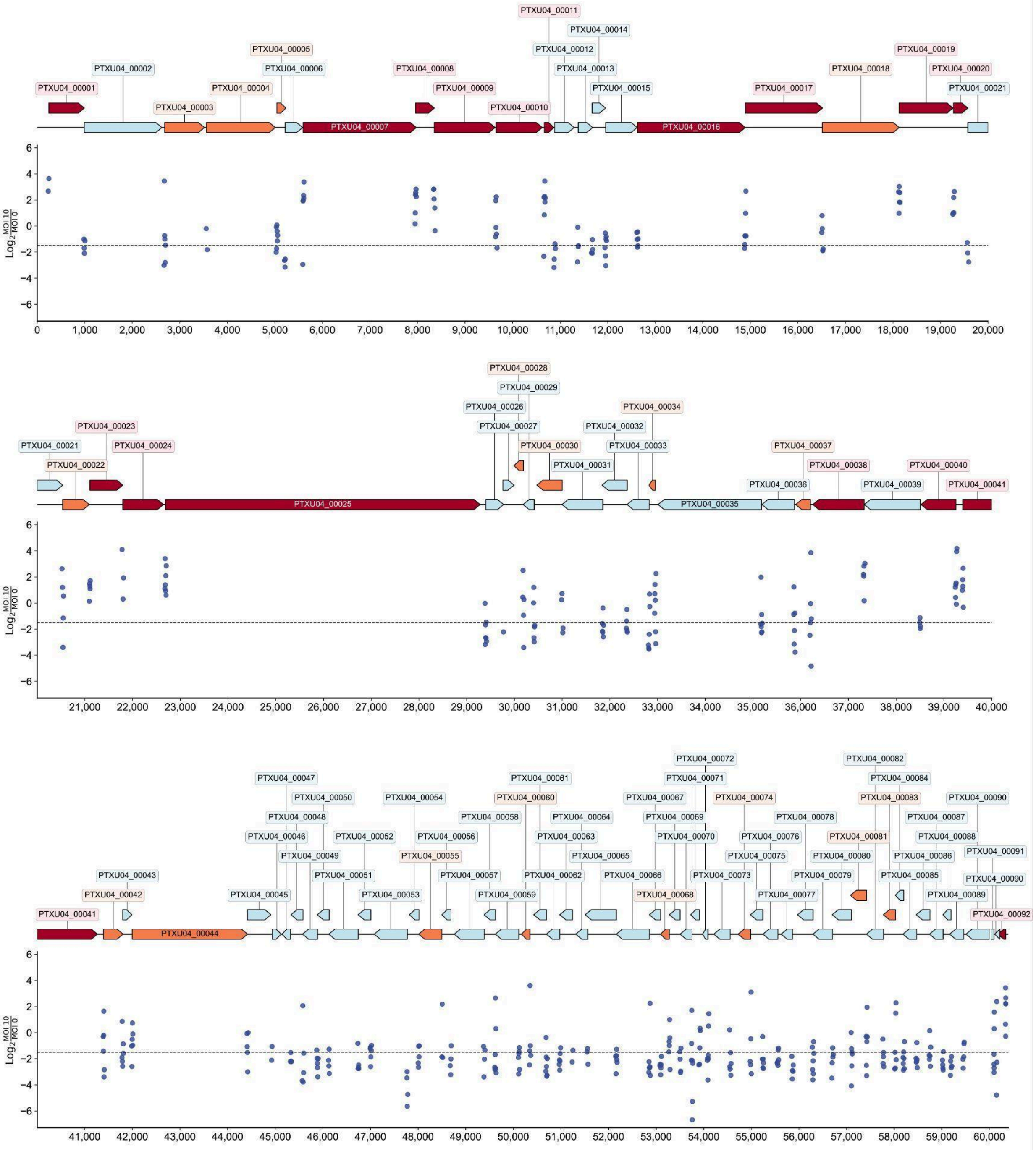

Supplementary Fig. 33 | Transcriptome-wide CRISPRi-ART Fitness for Phage PTXU04. (Top track) Gene overview of phage PTXU04 (MK373772). Coding genes are shown in light blue and highlighted in red if they met significance thresholds (Methods). tRNAs are shown in orange. Other noncoding genetic elements are shown in white. (Middle track) dCas13d gene knockdown fitness when targeting PTXU04 genes during infection at 10 MOI. Median guide fitness is shown with a dashed line. (Bottom track) dCas13d gene knockdown fitness when targeting PTXU04 genes during infection at 100

MOI. Median guide fitness is shown with a dashed line.

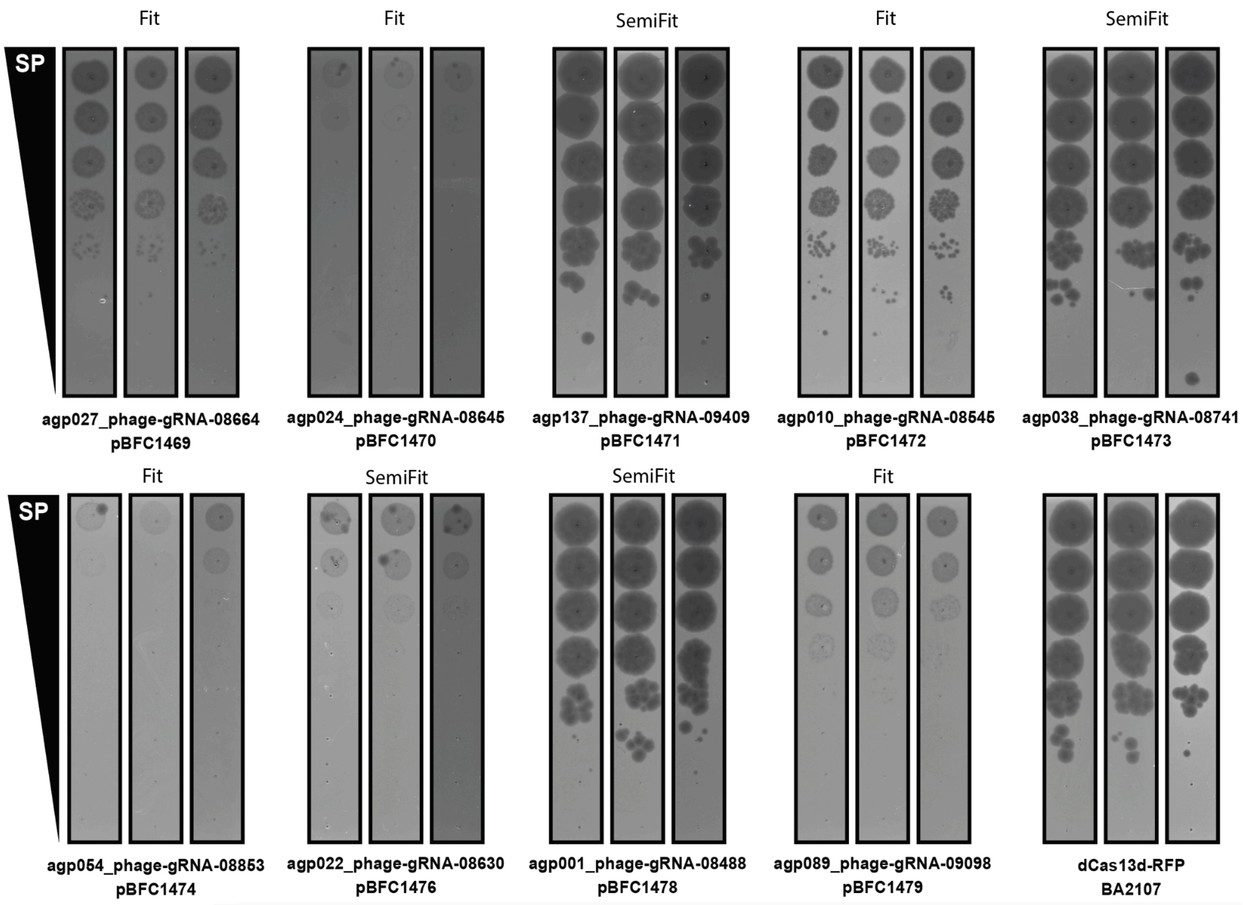

Supplementary Fig. 34 | SUSP1 CRISPRi-ART Single crRNA Validation Plaque Assays. Plaque assays to validated pooled CRISPRi-ART results using single crRNA to target phage SUSP1 RBS with dRfxCas13d. Guides were selected for their performance in the high-throughput screen. 9 genes of both known and unknown function of Semi-Fit and Fit classification were selected for plaque assay validation. A guide targeting RFP is provided as a negative control. dRfxCas13d was expressed using +100nM aTc. Data shown are for 3 biological replicates.

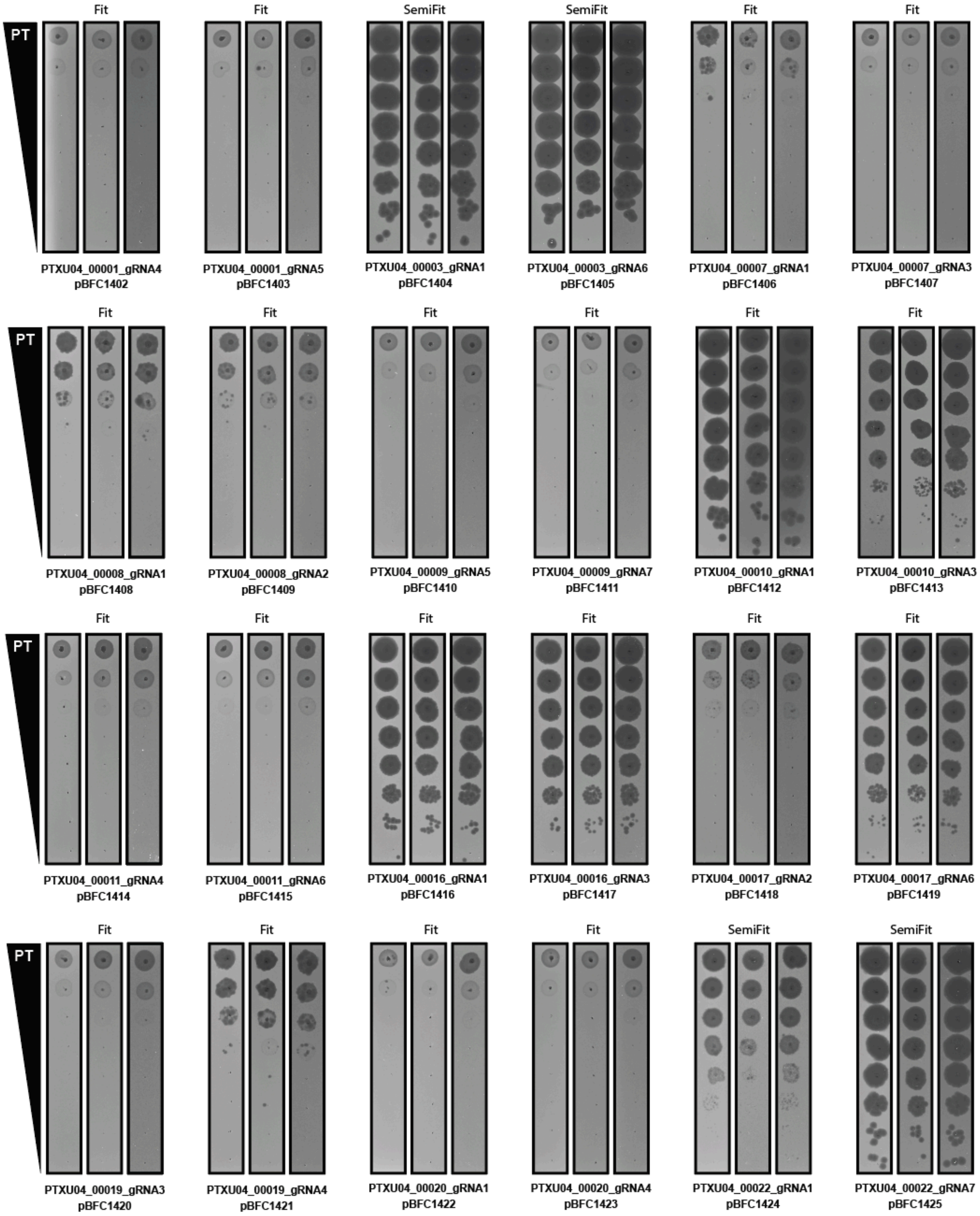

Supplementary Fig. 35 | PTXU04 CRISPRi-ART Validation Plaque Assays. (Continued Below)

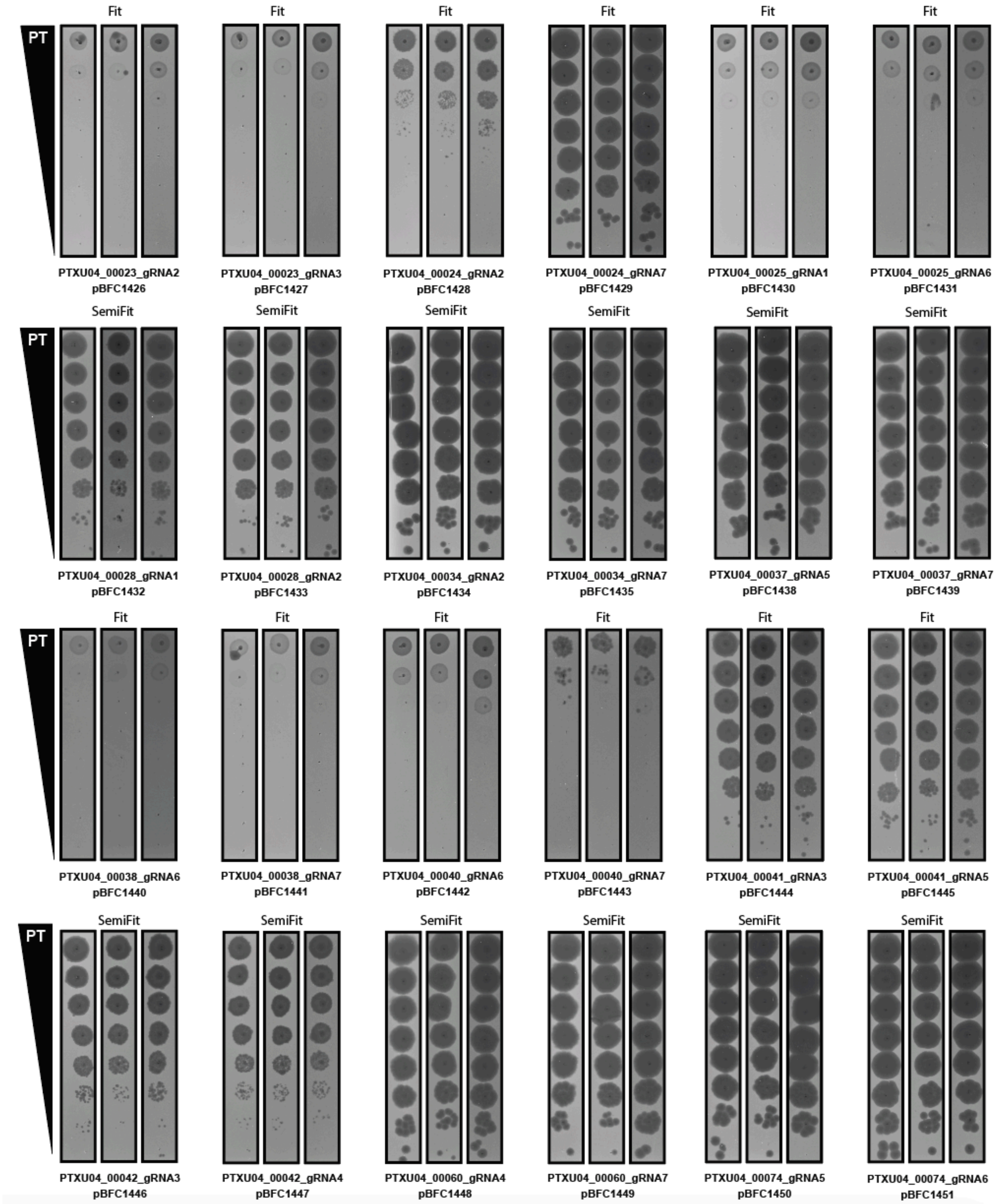

Supplementary Fig. 35 | PTXU04 CRISPRi-ART Validation Plaque Assays. (Continued Below)

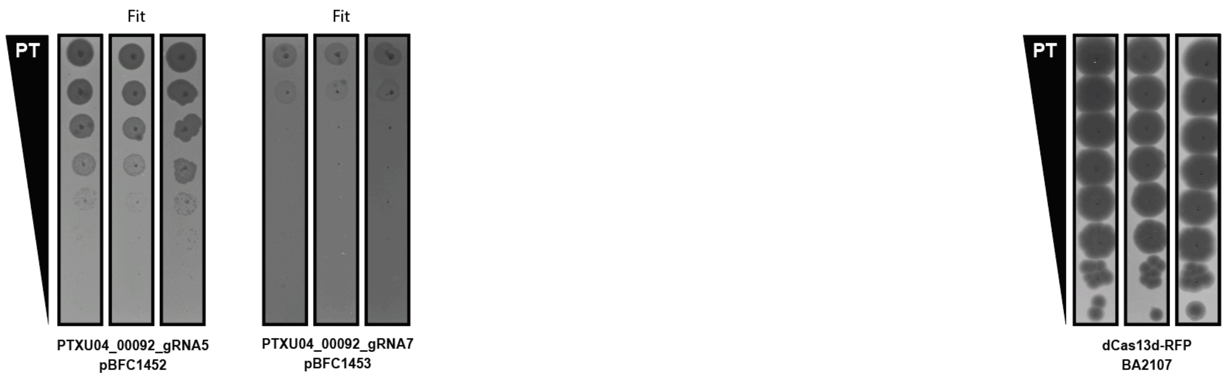

Supplementary Fig. 35 | PTXU04 CRISPRi-ART Validation Plaque Assays. Plaque assays for CRISPRi-ART-mediated phage defense when targeting phage PTXU04 RBS with dRfxCas13d. The two best performing guides for all genes labeled Fit or Semi Fit are shown here, based on their performance in the transcriptome-wide pooled fitness assay. A guide targeting RFP is provided as a negative control. dRfxCas13d experiments were expressed using +100nM aTc. Data shown are for 3 biological replicates.

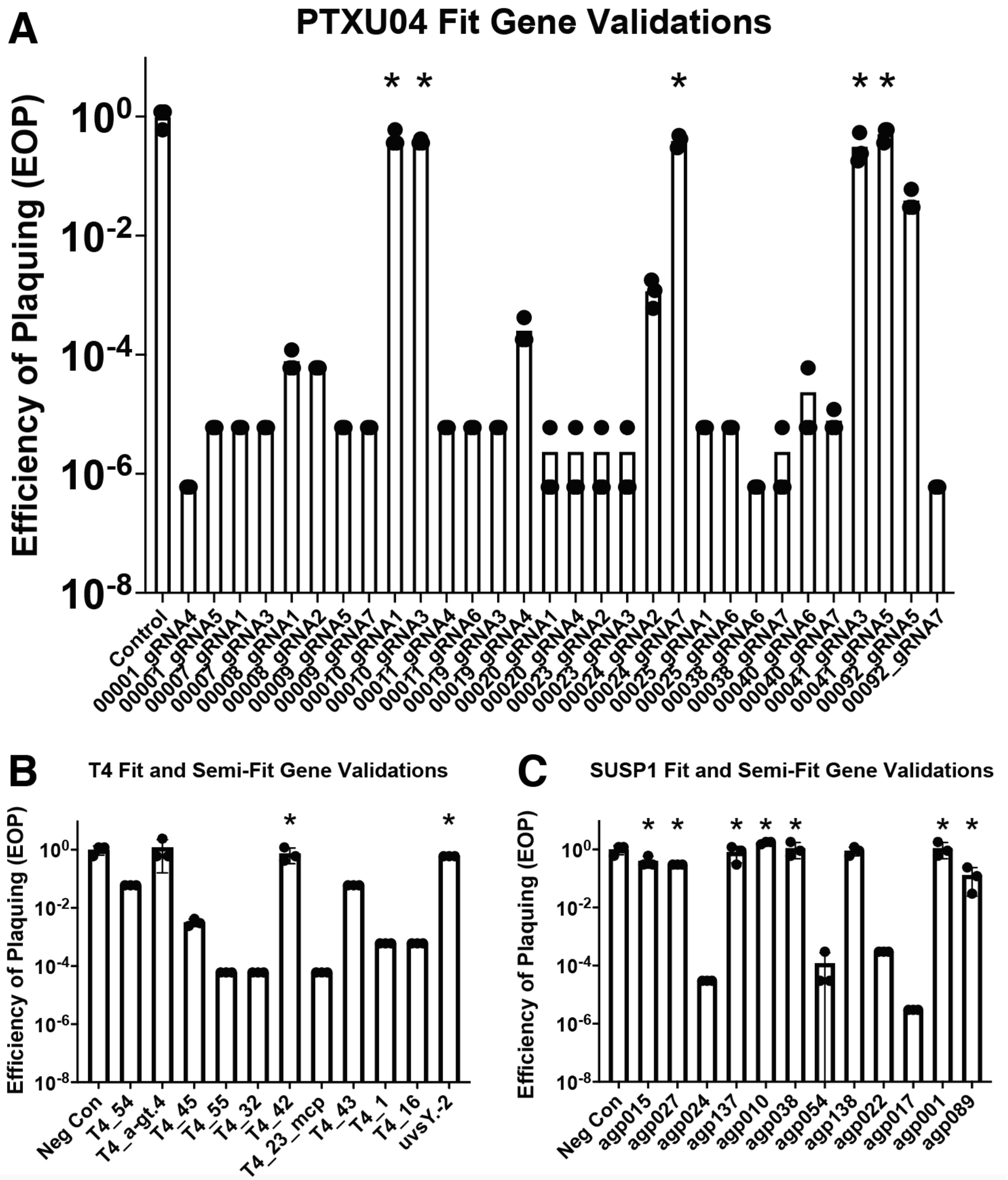

Supplementary Fig. 36 | CRISPRi-ART Validation Plaque Assays EOPs. EOP measurements obtained from CRISPRi-ART validation plaque assays for three different phage sets scaled to their respective non-targeting control. **a,** EOP measurements for PTXU04 Fit genes. **b,** T4 validation set. **c,** SUSP1 validation set. Guides that led to a significant reduction in plaque size compared to the non-targeting crRNA (Control, Neg Con) as measured by a two-way ANOVA and are indicated with an asterisk (adjusted

p-value < 0.0001) reported only when there is not a significant reduction in EOP. For **a-c**, data shown are for 3 biological replicates. Error bars are presented as mean +/- SD.

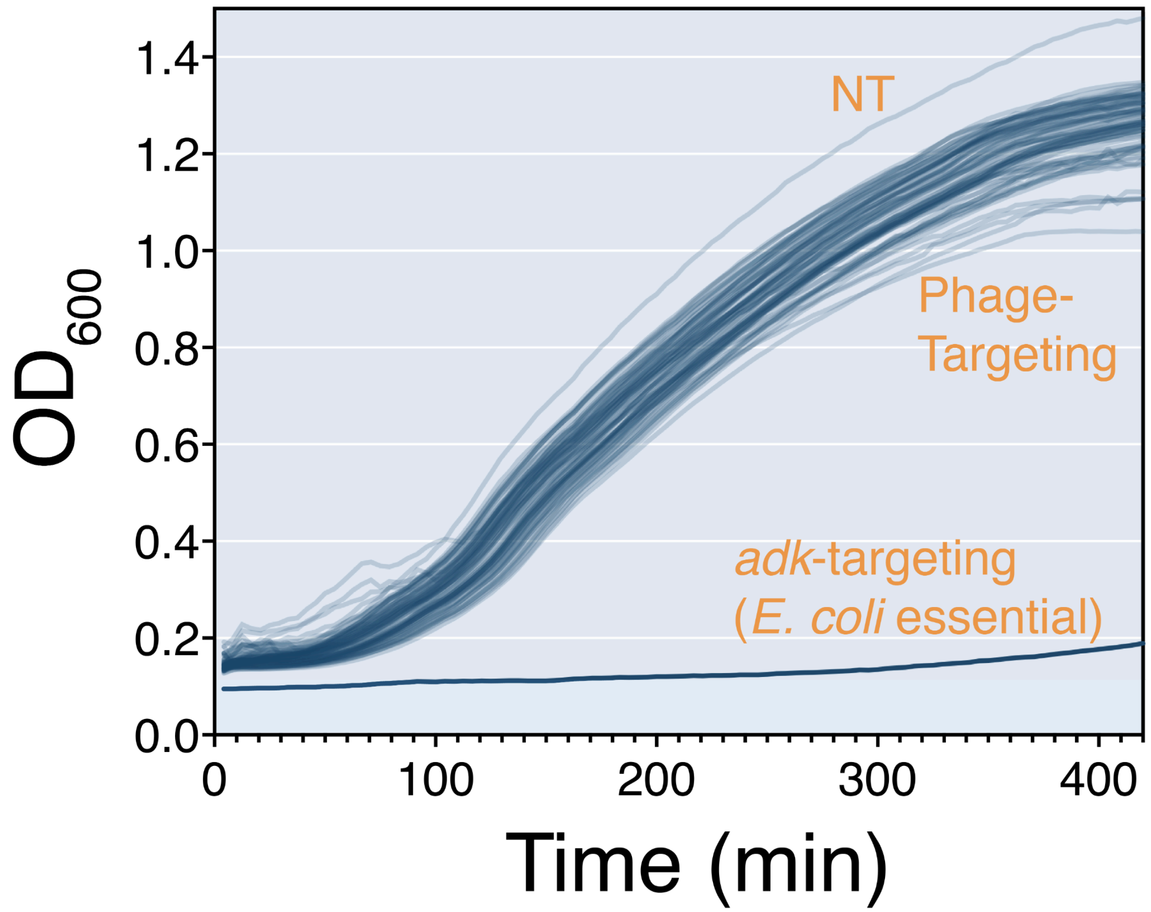

Supplementary Fig. 37 | Assessment of Phage-Targeting Fit crRNAs on *E. coli* Growth. Growth curves of *E. coli* expressing CRISPRi-ART targeting phage Fit and Semi-Fit genes. All crRNAs used for single crRNA plaque assays validating pooled fitness screens are tested here for *E. coli* growth inhibition. A crRNA targeting *E. coli* essential gene *adk* is provided as a positive control for CRISPRi-ART-mediated growth repression. Cultures were grown in the presence of 200 nM aTc. Optical density at 600 nm (OD_{600}) was measured at 5 min intervals to monitor bacterial growth over time. Experiments were performed in triplicate, and data are presented as mean of triplicates.

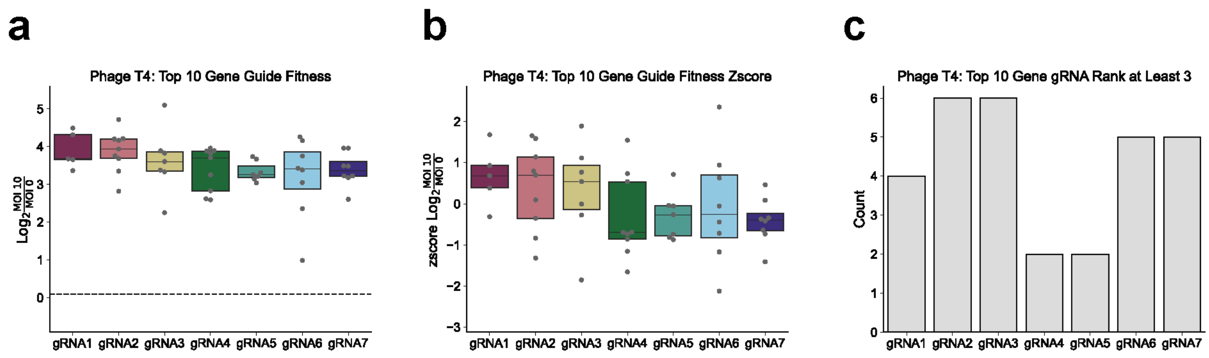

Supplementary Fig. 38 | crRNA Fitness Distributions for Top 10 Fit Phage T4 Genes. All analyses in Supplementary Fig. 25 refer to guides that target the Top 10 Fit Genes in T4 and pass quality control criteria (Methods). Genes with under 5 quality-control-passing guides were excluded. **a,** Guide fitness distribution separated by relative crRNA position. Dashed line reflects median guide fitness observed in the experiments. **b,** Z-score normalized guide fitness distribution (by targeted gene) separated by relative crRNA position. **c,** Number of guides performing in the Top 3 guides for a given gene. Central line in boxplots in **a** and **b** represent the median and edges of the boxplot represents the 25 and 75 confidence intervals. For **a-c**, data shown are for 3 biological replicates.

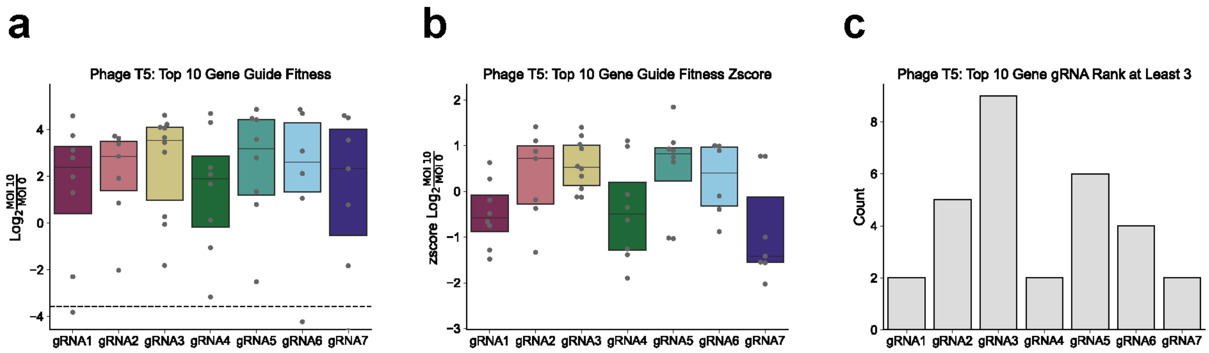

Supplementary Fig. 39 | crRNA Fitness Distributions for Top 10 Fit Phage T5 Genes. All analyses in Supplementary Fig. 26 refer to guides that target the Top 10 Fit Genes in phage T5 and pass quality control criteria (Methods). Genes with under 5 quality-control-passing guides were excluded. **a,** Guide fitness distribution separated by relative crRNA position. Dashed line reflects median guide fitness observed in the experiments. **b,** Z-score normalized guide fitness distribution (by targeted gene) separated by relative crRNA position. **c,** Number of guides performing in the Top 3 guides for a given gene. Central line in boxplots in **a** and **b** represent the median and edges of the boxplot represents the 25 and 75 confidence intervals. For **a-c**, data shown are for 3 biological replicates.

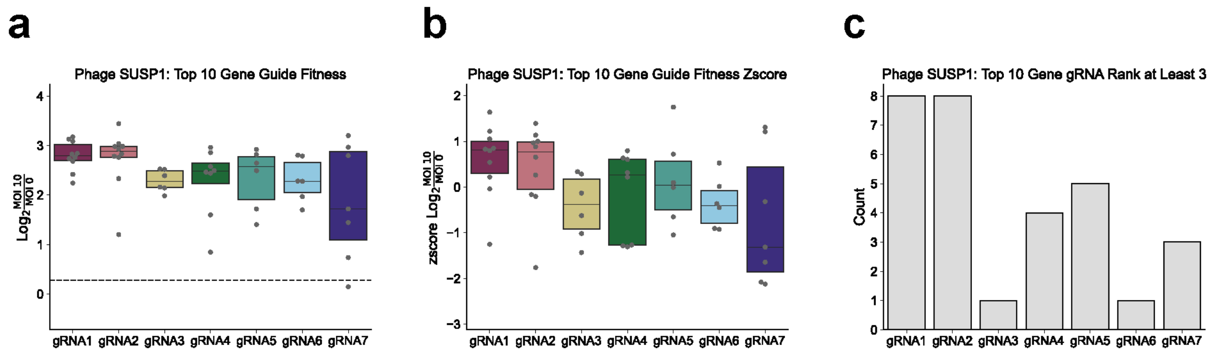

Supplementary Fig. 40 | crRNA Fitness Distributions for Top 10 Fit Phage SUSP1 Genes. All analyses in Supplementary Fig. 27 refer to guides that target the Top 10 Fit Genes in phage SUSP1 and pass quality control criteria (Methods). Genes with under 5 quality-control-passing guides were excluded. **a,** Guide fitness distribution separated by relative crRNA position. Dashed line reflects median guide fitness observed in the experiments. **b,** Z-score normalized guide fitness distribution (by targeted gene) separated by relative crRNA position. **c,** Number of guides performing in the Top 3 guides for a given gene. Central line in boxplots in **a** and **b** represent the median and edges of the boxplot represents the 25 and 75 confidence intervals. For **a-c**, data shown are for 3 biological replicates.

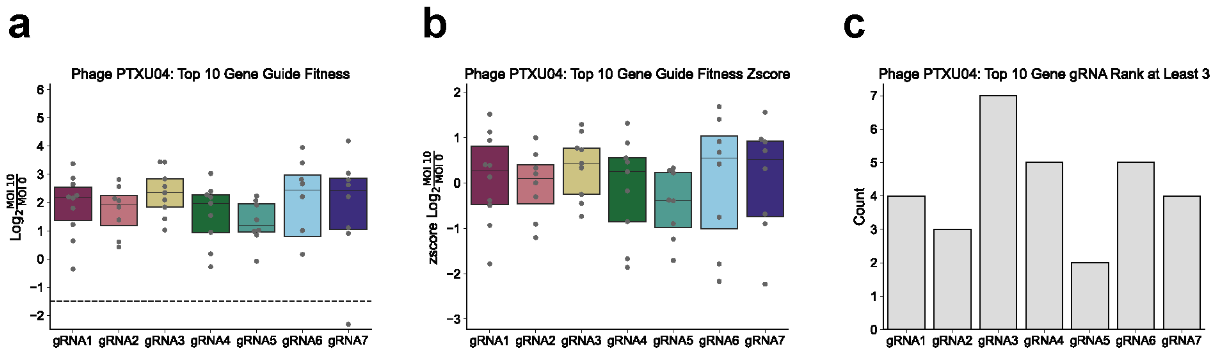

Supplementary Fig. 41 | crRNA Fitness Distributions for Top 10 Fit Phage PTXU04 Genes. All analyses in Supplementary Fig. 28 refer to guides that target the Top 10 Fit Genes in phage PTXU04 and pass quality control criteria (Methods). Genes with under 5 quality-control-passing guides were excluded. **a,** Guide fitness distribution separated by relative crRNA position. Dashed line reflects median guide fitness observed in the experiments. **b,** Z-score normalized guide fitness distribution (by targeted gene) separated by relative crRNA position. **c,** Number of guides performing in the Top 3 guides for a given gene. Central line in boxplots in **a** and **b** represent the median and edges of the boxplot represents the 25 and 75 confidence intervals. For **a-c**, data shown are for 3 biological replicates.

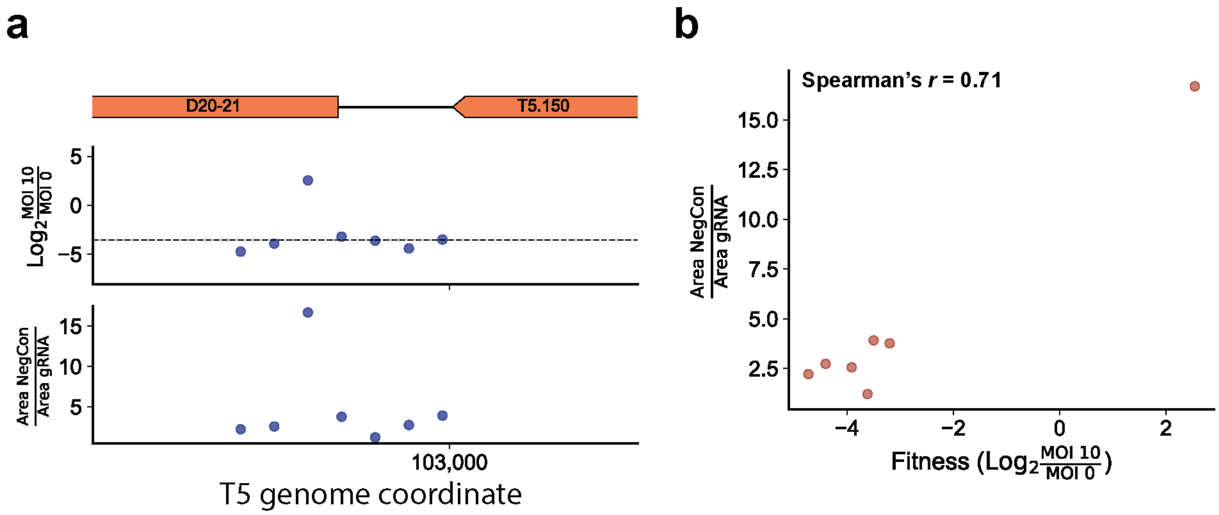

Supplementary Fig. 42 | Guide fitness versus plaque size for T5 *D20-21* (*mcp*). a, Fitness and fold plaque size reduction for CRISPRi-ART targeting of T5 *D20-21* (*mcp*) by position. Plots show gene organization at the *D20-21* locus (top), fitness values from pooled library screens (middle), and fold plaque size reduction from individually tested crRNAs (bottom). **b,** Correlation between fitness values from pooled library screens and fold plaque size reduction. Correlation is reported with Spearman's coefficient (*r*).

Supplementary Tables:

Supplementary Table S1.
Plasmids used in this study.

Supplementary Table S2.
Bacterial strains used in this study.

Supplementary Table S3.
Bacteriophages used in this study.

Supplementary Table S4.
Oligonucleotides used in this study, not including oligo pools.

Supplementary Table S5.
Data table describing metadata, raw and normalized read counts, crRNA fitness values.

Supplementary Table S6.
Curated phage genome annotations for phages T4, T5, SUSP1, and PTXU04.

Supplementary Table S7.
Plaque size measurements for all plaque assays in this study.

Supplementary Table S8.
Properties of investigated phages

References

1. Hintermann, E. & Kuhn, A. Bacteriophage T4 gene 21 encodes two proteins essential for phage maturation. *Virology* **189**, 474–482 (1992).
2. Miller Eric S. *et al.* Bacteriophage T4 Genome. *Microbiol. Mol. Biol. Rev.* **67**, 86–156 (2003).
3. Franklin, J. L. & Mosig, G. Expression of the bacteriophage T4 DNA terminase genes 16 and 17 yields multiple proteins. *Gene* **177**, 179–189 (1996).
4. Otsuka, Y. & Yonesaki, T. Dmd of bacteriophage T4 functions as an antitoxin against Escherichia coli LsoA and RnlA toxins. *Mol. Microbiol.* **83**, 669–681 (2012).
5. Hobbs, S. J. *et al.* Phage anti-CBASS and anti-Pycsar nucleases subvert bacterial immunity. *Nature* **605**, 522–526 (2022).
6. Huiting, E. *et al.* Bacteriophages inhibit and evade cGAS-like immune function in bacteria. *Cell* **0**, (2023).
7. Jenson, J. M., Li, T., Du, F., Ea, C.-K. & Chen, Z. J. Ubiquitin-like conjugation by bacterial cGAS enhances anti-phage defence. *Nature* **616**, 326–331 (2023).
8. Calendar, R. *The Bacteriophages*. (Springer US).
9. Davison, J. Pre-early functions of bacteriophage T5 and its relatives. *Bacteriophage* **5**, e1086500 (2015).
10. Heller, K. & Braun, V. Polymannose O-antigens of Escherichia coli, the binding sites for the reversible adsorption of bacteriophage T5+ via the L-shaped tail fibers. *J. Virol.* **41**, 222–227 (1982).
11. Wang, J. *et al.* Complete genome sequence of bacteriophage T5. *Virology* **332**, 45–65 (2005).

12. Zivanovic, Y. *et al.* Insights into bacteriophage T5 structure from analysis of its morphogenesis genes and protein components. *J. Virol.* **88**, 1162–1174 (2014).

13. Glukhov, A. S., Krutilina, A. I., Kaliman, A. V., Shlyapnikov, M. G. & Ksenzenko, V. N. Bacteriophage T5 Mutants Carrying Deletions in tRNA Gene Region. *Mol. Biol.* **52**, 1–6 (2018).

14. Keen, E. C. *et al.* Novel ‘Superspreader’ Bacteriophages Promote Horizontal Gene Transfer by Transformation. *MBio* **8**, (2017).

15. Whichard, J. M. *et al.* Complete genomic sequence of bacteriophage felix o1. *Viruses* **2**, 710–730 (2010).

16. Fong, K. *et al.* Diversity and Host Specificity Revealed by Biological Characterization and Whole Genome Sequencing of Bacteriophages Infecting Salmonella enterica. *Viruses* **11**, (2019).

17. Adler, B. A. *et al.* The genetic basis of phage susceptibility, cross-resistance and host-range in Salmonella. *Microbiology* **167**, (2021).

18. Darling, A. E., Mau, B. & Perna, N. T. progressiveMauve: multiple genome alignment with gene gain, loss and rearrangement. *PLoS One* **5**, e11147 (2010).

19. Young, R. Phage lysis: three steps, three choices, one outcome. *J. Microbiol.* **52**, 243–258 (2014).

20. Dwivedi, B., Xue, B., Lundin, D., Edwards, R. A. & Breitbart, M. A bioinformatic analysis of ribonucleotide reductase genes in phage genomes and metagenomes. *BMC Evol. Biol.* **13**, 33 (2013).

21. Adler, B. A. *et al.* Broad-spectrum CRISPR-Cas13a enables efficient phage genome editing. *Nat Microbiol* **7**, 1967–1979 (2022).

22. Iyer, L. M., Burroughs, A. M., Anand, S., de Souza, R. F. & Aravind, L. Polyvalent Proteins, a Pervasive Theme in the Intergenomic Biological Conflicts of Bacteriophages and Conjugative Elements. *J. Bacteriol.* **199**, (2017).

23. Zimmermann, L. *et al.* A Completely Reimplemented MPI Bioinformatics Toolkit with a New HHpred Server at its Core. *J. Mol. Biol.* **430**, 2237–2243 (2018).

24. Piya, D. *et al.* Genome-wide CRISPRi knockdown to map gene essentiality landscape in coliphages λ and P1. *bioRxiv* 2023.05.14.540688 (2023) doi:10.1101/2023.05.14.540688.

25. Miller, E. S. *et al.* Bacteriophage T4 Genome. *Microbiol. Mol. Biol. Rev.* (2003) doi:10.1128/mmbr.67.1.86-156.2003.
